# Selective mRNA translation determines adaptative mutability of melanoma cells to anti-BRAF/MEK combination therapy

**DOI:** 10.1038/s44321-026-00479-5

**Published:** 2026-07-20

**Authors:** Lucilla Fabbri, Lucie Lagadec, Eva Guérin, Hélène Lecourt, Dorothée Baille, Laetitia Besse, Cédric Messaoudi, Laurent Désaubry, Hussein Abou-Hamdan, Sévérine Roy, Bérangère Lombard, Damarys Loew, Jean-Yves Scoazec, Caroline Robert, Stéphan Vagner

**Affiliations:** 1 Institut Curie, PSL Research University, CNRS UMR3348, INSERM U1368, Orsay, France; 2 Université Paris-Saclay, CNRS UMR3348, INSERM U1368, Orsay, France; 3 Equipe labellisée Ligue Nationale contre le Cancer, Orsay, France; 4 INSERM U981, Gustave Roussy Cancer Campus, Villejuif, France; 5 Université Paris-Sud, Université Paris-Saclay, Kremlin-Bicêtre, France; 6 Dermato-Oncology, Gustave Roussy Cancer Campus, Villejuif, France; 7 Institut Curie, PSL University, CNRS UAR2016, Inserm US43, Université Paris-Saclay, Multimodal Imaging Center, Orsay, France; 8 Center of Research in Biomedicine of Strasbourg, Regenerative Nanomedicine (UMR 1260), INSERM, University of Strasbourg, Strasbourg, France; 9 Institut Curie, PSL University, CurieCoreTech Mass Spectrometry Proteomics, Paris, France; 10 Institut Gustave Roussy, INSERM U1015, Bâtiment de Médecine Moléculaire 114 rue Edouard Vaillant, 94800 Villejuif, France

**Keywords:** Cancer, Skin, Translation & Protein Quality

## Abstract

During their inevitable evolution towards acquired resistance to anti-cancer targeted therapies, cancer cells adopt distinct gene expression profiles that allow them to transiently adapt to and tolerate the treatment. Cancer cells surviving therapy can increase their mutation rate, enhancing the likelihood of acquiring resistance-conferring mutations and evolving into resistant cells. Here we show that translational control mediates the adaptive mutability of melanoma drug-tolerant cells by regulating the translation of the error-prone non-homologous end joining (NHEJ) component *53BP1*. The specific inhibition of 5’UTR-driven *53BP1* mRNA translation was sufficient to impair NHEJ and mutability. We found that the eIF4A RNA helicase, regulates *53BP1* mRNA translation. Consequently, targeting the eIF4A with two small molecule inhibitors significantly delays the acquisition of resistance to combination of BRAF and MEK inhibitors in BRAF^V600^-mutant melanoma xenograft models and cell lines by reducing the mutability of drug-tolerant cells. Our results demonstrate that a standard-of-care therapy for melanoma, by engaging non-genetic adaptation driven at the translational level, contributes to the evolution of drug-tolerant melanoma cells toward acquired resistance.

## Synopsis

Targeted BRAFi/MEKi therapy in BRAFV600-mutant melanoma induces translational reprogramming that promotes adaptive mutability and acquired resistance, creating a therapeutic vulnerability to eIF4A inhibition.

- 5′UTR-dependent translation of the mRNA encoding the NHEJ factor 53BP1 was enhanced in drug-tolerant cells.
- This translational reprogramming was associated with a shift toward error-prone NHEJ and increased mutability.
- eIF4A inhibition reduced 53BP1 translation and delayed the emergence of resistance in xenograft models.

## Abstract (web summary)

Targeted BRAFi/MEKi therapy in BRAFV600-mutant melanoma induces translational reprogramming that promotes adaptive mutability and acquired resistance, creating a therapeutic vulnerability to eIF4A inhibition.

## Graphical abstract

**Figure:**
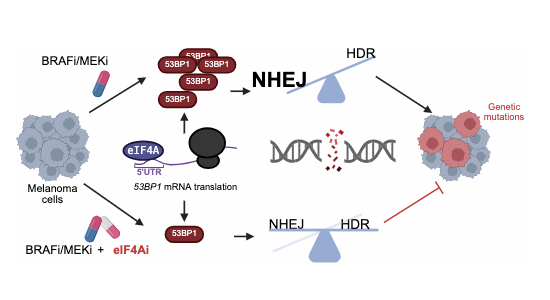

**Figure:**
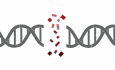

The paper explained **Problem**

Targeted therapy combining BRAF and MEK inhibitors (BRAFi/MEKi) has revolutionized the treatment of metastatic melanoma harboring the BRAFV600 mutation. Despite the initial clinical benefits, which achieve response rates of up to 70%, most patients eventually experience disease progression within several months due to the development of acquired resistance. The evolution towards resistance has been ascribed to multiple non-genetic mechanisms underpinning cells initially tolerant to the treatment. However, effective therapeutic strategies to prevent or limit resistance remain lacking.

**Results**

We found that selective mRNA translation leads to reprogramming of DNA repair mechanisms. BRAFi/MEKi treatment induces 5’UTR-mediated translation regulation of the mRNA encoding the non-homologous end-joining (NHEJ) factor 53BP1, which was associated with enhanced reliance on the error-prone NHEJ pathway in melanoma cells that survived BRAFi/MEKi therapy. This was associated with mutation frequency at the HPRT locus, indicating increased mutability in drug-tolerant cells. We identified the RNA helicase eIF4A as a key regulator of *53BP1* mRNA translation. Pharmacological inhibition of eIF4A using two independent small-molecule inhibitors reduced *53BP1* translation, impaired NHEJ activity, and decreased mutability in BRAFi/MEKi-tolerant melanoma cells. Accordingly, combining BRAF, MEK and eIF4A inhibitors delayed the emergence of resistance in xenograft models.

**Impact**

This study identifies translational control as a driver of acquired resistance to targeted therapy in melanoma. Targeting 53BP1 mRNA translation by inhibiting eIF4A may constitute a promising therapeutic strategy to limit the evolution of resistance to BRAFi/MEKi therapy in BRAFV600-mutant melanoma.

## Introduction

Targeted therapy combining BRAF and MEK inhibitors (BRAFi/MEKi) has revolutionized the treatment of metastatic melanoma harboring the BRAF^V600^ mutation, which is present in ~50% of patients (Davies et al, 2002; Menzies et al, 2012). Despite the initial clinical benefits, which achieve response rates of up to 70%, most patients eventually experience disease progression within several months due to the development of acquired resistance (Eroglu and Ribas, 2016; Robert et al, 2015).

In addition to genetic mutations, recent findings have revealed the existence of non-genetic mechanisms that underpin drug tolerance in a subset of cancer cells and drive evolution towards resistance (Marine et al, 2020; Shaffer et al, 2017; Sharma et al, 2010). Although the drug-tolerant state may initially be reversible, prolonged drug exposure can lead to increased mutation rates in drug-tolerant cells, ultimately promoting the emergence of clones characterized by stable, genetic resistance and cancer relapse (Cipponi et al, 2020; Hata et al, 2016; Russo et al, 2019). Such adaptive mutability has been shown to be threatened by accumulating DNA double-strand breaks (DSBs), even upon exposure to nongenotoxic therapies (Ali et al, 2022; Cipponi et al, 2020; Russo et al, 2024; Russo et al, 2019). In addition to the error-free homology-directed repair (HDR) pathway, which is restricted to the S and G2 phases of the cell cycle, DSB repair is also mediated by the non-homologous end-joining (NHEJ) pathway (Lieber et al, 2003). Unlike HDR, NHEJ operates throughout all phases of the cell cycle but is error-prone, potentially leading to deleterious mutations or deletions that may contribute to adaptive mutability. In melanoma, BRAFi/MEKi therapy has been proposed to induce transcriptional rewiring of DNA repair genes, leading to the suppression of error-free pathways, such as HDR, and the emergence of a BRCAness-like state (Lord and Ashworth, 2016; Maertens et al, 2019). Additionally, recent findings implicated the NHEJ pathway in the acquisition of genetic instability of melanoma following targeted therapy, promoting the evolution of acquired resistance (Dharanipragada et al, 2023).

Dynamic and transient changes in gene expression, through epigenetic, transcriptional or translational reprogramming, have been implicated in fostering cellular plasticity and enabling melanoma cells to adapt to therapy (Chauvistré et al, 2022; Rambow et al, 2018; Roesch et al, 2013; Shen et al, 2019). Adaptive translational reprogramming has emerged as a critical regulator of melanoma tolerance and acquisition of resistance to targeted therapies (Boussemart et al, 2014; Fabbri et al, 2021; Falletta et al, 2017; Rapino et al, 2018; Shen et al, 2019; Smith et al, 2022). We demonstrated that the emergence of non-genetic drug-tolerant melanoma cells is linked to eIF4F-mediated mRNA translation (Shen et al, 2019). This complex, consisting of the eIF4E cap-binding protein, the eIF4G scaffolding protein, and the eIF4A RNA helicase, binds to the cap structure at the 5’-end of mRNAs to mediate cap-dependent translation (Pelletier and Sonenberg, 2019). Although the persistent formation of the eIF4F translation initiation complex has been associated with stable genetic resistance to BRAFi/MEKi therapy in BRAF^V600^-mutant melanoma (Boussemart et al, 2014), its potential role in driving the evolution of drug-tolerant cells toward acquired resistance through adaptive mutability remains unexplored.

Here we show that, in melanoma drug-tolerant cells, the adaptive mRNA translation reprogramming occurring following targeted therapy enhances the translation of *53BP1* mRNA, which encodes a crucial NHEJ promoter (Panier and Boulton, 2014). This correlates with enhanced 53BP1-dependent mutagenesis and a shift toward NHEJ-directed repair. Notably, CRISPR/Cas9-mediated partial deletion of the *53BP1* 5′ UTR in drug-tolerant cells reduces NHEJ activity and impairs mutability, underscoring the importance of translational control.

We identify eIF4A as a key regulator of *53BP1* mRNA translation. Treatment with the clinically advanced eIF4A inhibitor eFT226 significantly impairs *53BP1* mRNA translation, NHEJ activity, and the mutability of drug-tolerant cells. Furthermore, co-treatment with BRAFi/MEKi and either eFT226 or a newly developed, selective eIF4A inhibitor, RBX0901, delays the onset of acquired resistance in mouse xenograft models. Collectively, our findings reveal that translational regulation of *53BP1* mRNA is associated with the transition from a reversible drug-tolerant state to permanent genetic resistance by promoting NHEJ and mutability. Targeting this regulatory axis by inhibiting eIF4A may offer a potential therapeutic strategy to limit tumor evolution and recurrence.

## Results

### Targeted therapy increases the expression of the NHEJ protein 53BP1

**Figure 1:**
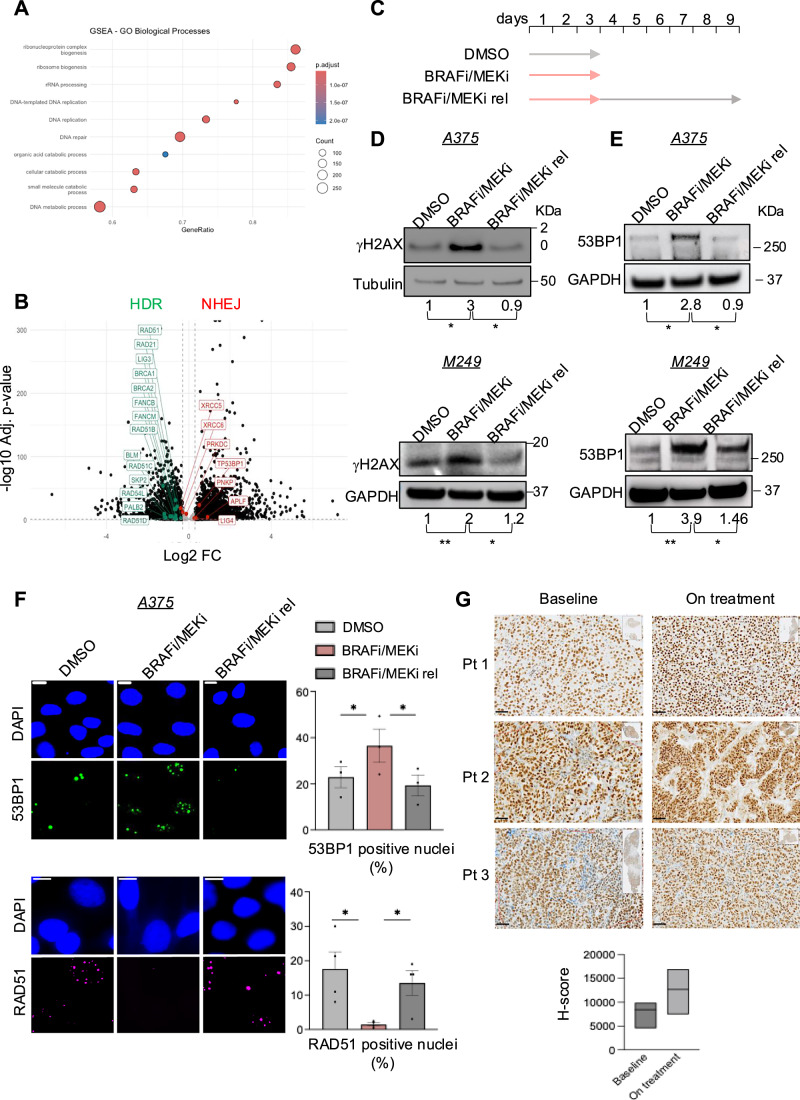
Targeted therapy increases the expression of the NHEJ protein 53BP1. (**A**) Dot plot showing significantly enriched gene sets of differentially expressed proteins in BRAF^V600E^-mutated A375 melanoma cells surviving BRAFi/MEKi therapy (BRAFi/MEKi) versus A375 cells treated with DMSO using GSEA. The color of the bubbles represents the *p* value adjusted, and the size of the bubbles represents the number of enriched genes from each pathway. *P* values were adjusted for multiple comparisons using the Benjamini–Hochberg false discovery rate (FDR) method. (**B**) Volcano plots showing *P* values adjusted (−log_10_) versus the log_2_ fold change (FC) of the proteomics analysis of BRAFi/MEKi-treated A375 cells versus DMSO-treated cells (*n* = 3); non-significant proteins are shown in gray, and proteins involved in HDR and NHEJ pathways are highlighted in green and red, respectively, and key proteins are labeled. Statistical significance was assessed using a linear model followed by a two-sided t-test on the model-estimated fold change; *p* values were corrected using the Benjamini–Hochberg FDR method. (**C**) Schematic representation of cell treatments used for subsequent analysis. Cells were treated with DMSO (control) or with targeted therapy (BRAFi/MEKi) for 3 days. Cells that survived therapy were recultured in drug-free media for an additional 9 days (BRAFi/MEKi rel). (**D**) Western blot illustrating γH2AX levels in A375 and M249 cells treated with DMSO, in cells surviving targeted therapy (BRAFi/MEKi) or in cells released from the drugs for 9 days (BRAFi/MEKi rel). GAPDH or Tubulin serves as a loading control, and the relative quantification from three independent experiments is indicated (unpaired Student’s *t*-test, A375: DMSO vs BRAFi/MEKi *p = 0.0256, BRAFi/MEKi vs BRAFi/MEKi rel *p = 0.0261, M249: DMSO vs BRAFi/MEKi **p = 0.0027, BRAFi/MEKi vs BRAFi/MEKi rel *p = 0,0120). (**E**) Western blot illustrating 53BP1 levels in A375 and M249 cells treated with DMSO, in cells surviving targeted therapy (BRAFi/MEKi) or in cells released from the drugs for 9 days (BRAFi/MEKi rel). GAPDH serves as a loading control, and the relative quantification from three independent experiments is indicated (unpaired Student’s *t*-test, A375: DMSO vs BRAFi/MEKi *p = 0.0292, BRAFi/MEKi vs BRAFi/MEKi rel *p = 0.0268, M249: DMSO vs BRAFi/MEKi **p = 0.0092, BRAFi/MEKi vs BRAFi/MEKi rel *p = 0.0237). (**F**) (top) Representative images of immunofluorescence in A375 control (DMSO), drug-tolerant cells (BRAFi/MEKi) and drug-tolerant cells recultured and released from targeted therapy for 9 days (BRAFi/MEKi rel), stained for 53BP1 (green) and nuclei (DAPI, blue). (bottom) Representative images of immunofluorescence in cells stained for RAD51 (magenta) and nuclei (DAPI, blue). Scale bar, 10 μm. The quantification of the signal analyzed by percentage of cells with ≥10 foci/nucleus is reported, and the data shown represent the mean ± SEM from three independent experiments. *p* values were calculated by paired, two-tailed Student’s *t*-test (53BP1: DMSO vs BRAFi/MEKi *p = 0.0486, BRAFi/MEKi vs BRAFi/MEKi rel *p = 0.0268, RAD51: DMSO vs BRAFi/MEKi *p = 0.0410, BRAFi/MEKi vs BRAFi/MEKi rel *p = 0.0422). (**G**) (top) Representative images of immunohistochemistry for 53BP1 in melanoma patient biopsies collected at baseline or ON treatment. Analysis of the H-score of 53BP1 staining in matched baseline and ON treatment biopsies is shown (bottom). H-score values are shown as the range from minimum to maximum. The horizontal line indicates the median. *n* = 3 matched biopsy pairs. Source data are available online for this figure.

**Figure EV1:**
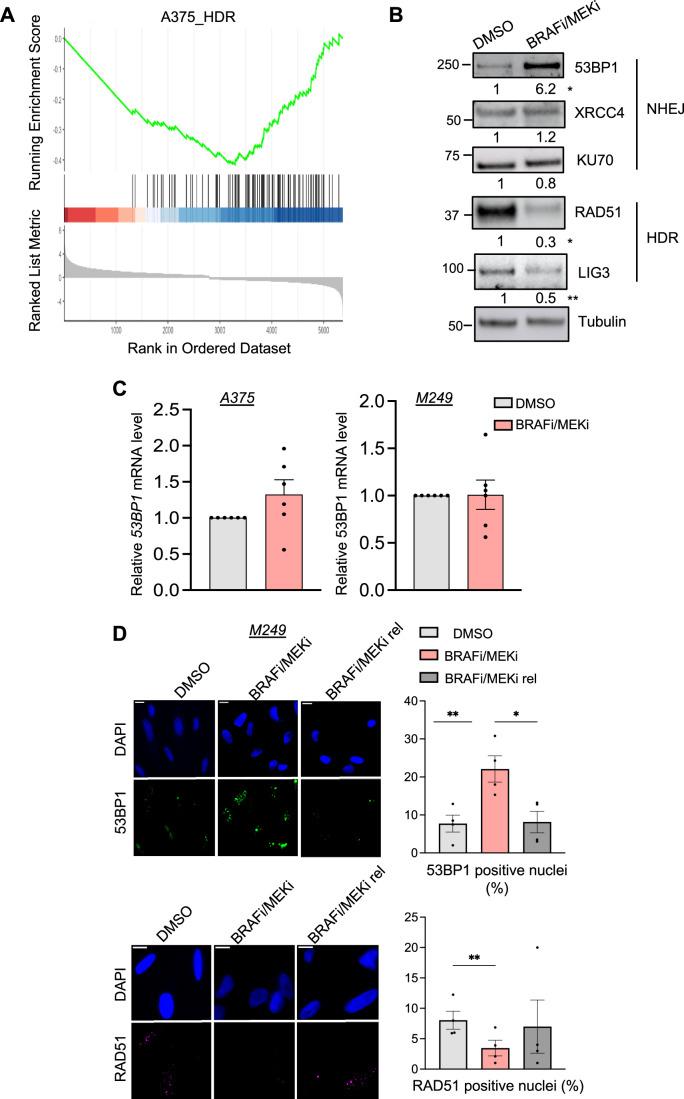
Monitoring DNA repair upon targeted therapy. (**A**) GSEA enrichment of the differentially expressed proreins. NES = −2.321, *q* value = 2.81e^−05^. (**B**) Western blot illustrating 53BP1, XRCC4, KU70, RAD51, and LIG3 protein levels in A375 cells after 3 days of treatment with DMSO (DMSO) or BRAFi/MEKi treatment (BRAFi/MEKi). GAPDH serves as a loading control, and the relative quantification from three independent experiments is indicated. *p* values were calculated by unpaired, two-tailed Student’s *t*-test (53BP1: *p = 0.0373, XRCC4: *p* = 0.3820, KU70: *p* = 0.3948, RAD51: *p = 0.0133, LIG3: **p = 0.0012). (**C**) RT-qPCR quantification of *53BP1* mRNA level in control cells (DMSO) or in BRAFi/MEKi A375 and M249 drug-tolerant cells. Data represent the mean ± SEM of six independent experiments. Data were normalized using the geometric mean of TBP, Actin and 18S housekeeping genes (A375: *p* = 0.1445, M249: *p* = 0.9540). (**D**) Representative images of immunofluorescence in M249 control (DMSO), drug-tolerant cells (BRAFi/MEKi) and drug-tolerant cells released from targeted therapy for 9 days (BRAFi/MEKi rel) stained for 53BP1 (green) and nuclei (DAPI, blue). (bottom) Representative images of immunofluorescence in cells stained for RAD51 (magenta) and nuclei (DAPI, blue). Scale bar, 10 μm. The quantification of the signal analyzed by percentage of cells with ≥10 foci/nucleus is reported, and data represent the mean ± SEM from four independent experiments. *p* values were calculated by paired, two-tailed Student’s *t*-test (53BP1: DMSO vs BRAFi/MEKi **p = 0.0077, BRAFi/MEKi vs BRAFi/MEKi rel *p = 0.0430, RAD51: DMSO vs BRAFi/MEKi **p = 0.0012, BRAFi/MEKi vs BRAFi/MEKi rel *p* = 0.4765). Source data are available online for this figure.

Quantitative proteomic analysis performed on BRAF^V600E^-mutated A375 melanoma cells that survived 3 days of exposure to high-dose BRAF/MEK inhibitors (BRAFi/MEKi) revealed a profound reprogramming of the proteome compared to untreated cells (DMSO), with DNA repair–related pathways ranking among the top 10 most dysregulated ones (Fig. 1A,B). The HDR pathway was markedly downregulated in BRAFi/MEKi-surviving A375 cells, supporting the hypothesis that targeted therapy promotes a BRCAness-like phenotype (Fig. EV1A). By employing another BRAF^V600E^ mutated cell line sensitive to MAPK pathway inhibition (Nazarian et al, 2010), we observed an increase in γH2AX levels in A375 and M249 cells that survived BRAFi/MEKi (Fig. 1C,D), suggesting potential DNA damage induced by the treatment. Such an increase was specifically associated with drug exposure, as re-culturing therapy-surviving cells in drug-free media for 9 days decreased the quantity of the protein to levels observed in untreated cells (Fig. 1C,D), thereby suggesting that therapy-induced DNA damage is reversible upon drug withdrawal.

It is known that NHEJ can compensate for defects in HDR (Dietlein et al, 2014; Maertens et al, 2019). Our proteomic analysis showed that protein levels of NHEJ-related factors were either maintained or increased following BRAFi/MEKi exposure (Figs. 1B and EV1B). To identify potential mediators of NHEJ with increased mRNA translation following BRAFi/MEKi-targeted therapy, we reanalyzed our previous genome-wide polysome profiling data (Shen et al, 2019). Among the translationally upregulated mRNAs, we selected the one encoding the key NHEJ mediator 53BP1, which appeared as being the only translationally regulated mRNA encoding an NHEJ component. 53BP1 is a chromatin-binding protein that regulates the DSB repair pathway choice by promoting canonical NHEJ-mediated DSB repair (Panier and Boulton, 2014). BRAFi/MEKi treatment led to a reversible increase in 53BP1 protein levels, as analyzed by western blot (Fig. 1E), without significant changes in *53BP1* mRNA levels despite an observed increase (less than twofold) in two out of six replicates in A375 cells (Fig. EV1C).

Analysis of the nuclear recruitment of 53BP1 and RAD51, a key effector of HDR, revealed that BRAFi/MEKi treatment increased the number of nuclei positive for 53BP1, while decreasing the ones positive for RAD51 in both A375 and M249 cells (>10 foci/nucleus) (Figs. 1F and EV1D). Upon drug withdrawal, nuclear recruitment patterns of these proteins returned to levels similar to those observed in untreated cells (Figs. 1F and EV1D), indicating that, in the two tested BRAF^V600E^ mutated melanoma cell lines, targeted therapy reprograms DNA repair by reversibly shifting the balance from HDR to NHEJ.

We next investigated 53BP1 protein levels in a pilot analysis of matched clinical biopsy specimens from 3 melanoma patients collected at baseline and upon BRAFi/MEKi exposure. Samples collected on treatment showed a trend towards increased 53BP1 protein levels compared to baseline (Fig. 1G). While this observation is based on a limited sample set, it suggests that the induction of 53BP1 upon targeted therapy could extend as well to clinical specimens. Altogether, these results show that 53BP1 expression is modulated upon targeted therapy.

### 5’UTR-dependent *53BP1* mRNA translation is induced upon targeted therapy

**Figure 2:**
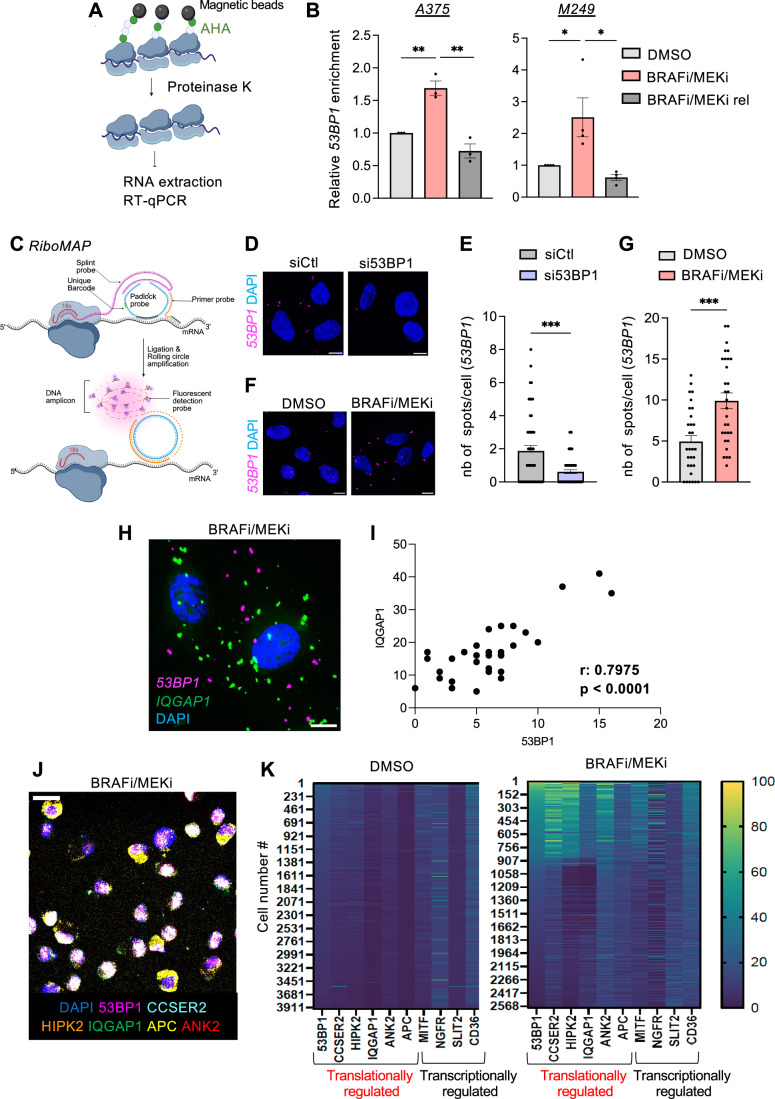
5’UTR-dependent *53BP1* mRNA translation is induced upon targeted therapy. (**A**) Schematic representation of the protocol used for the isolation of actively translating ribosomes and their associated transcripts. After a short L-azidohomoalanine (AHA) pulse, newly synthesized AHA-labeled peptides are used to isolate active ribosome complexes through chemical interactions with magnetic beads. Bead-bound complexes are then treated with Proteinase K, and the purified RNA is used for RT-qPCR. (**B**) Quantification of *53BP1* mRNA enrichment in active ribosomes was performed using RT-qPCR in A375 and M249 control cells (DMSO), in cells surviving targeted therapy (BRAFi/MEKi) and in cells released from the drugs for 9 days (BRAFi/MEKi rel). Data represent the mean ± SEM from 3 (A375, unpaired Student’s *t*-test **p = 0.0032 for the comparison BRAFi/MEKi vs DMSO and *p* = 0.0033 for the comparison BRAFi/MEKi vs BRAFi/MEKi rel) or 4 (M249, unpaired Student’s *t*-test *p = 0.0486 for the comparison BRAFi/MEKi vs DMSO and *p* = 0.0223 for the comparison BRAFi/MEKi vs BRAFi/MEKi rel) independent experiments. (**C**) Schematic of the ribosome-bound mRNA mapping (RIBOmap) used to detect translating mRNAs. RIBOmap relies on the use of a tri-probe set: (1) a primer probe that hybridizes to the target mRNAs, (2) a splint DNA probe that hybridizes with the ribosomal 18S RNA, and (3) a padlock probe. When in proximity, the tri-probes produce DNA amplification signals corresponding to active mRNA translation. (**D**) Representative confocal images of translating 53*BP1* mRNAs (magenta) in A375 cells transfected with control siRNA (siCtl, 25 nM) or with siRNA targeting *53BP1* (si53BP1, 25 nM) for 48 h. Nuclei are stained with DAPI (blue). Scale bar: 10 µm. (**E**) Quantification of translating *53BP1* mRNAs (spots) in the condition described in (**D**). Data shown represent the mean ± SEM of the number of spots/cells quantified in one independent experiment (see also Fig. EV2B for the other biological replicates) (unpaired Student’s *t*-test ***p = 0.0002). (**F**) Representative confocal images of translating 53*BP1* mRNAs (magenta) assessed by RiboMap assay in A375 control cells (DMSO) or in cells surviving targeted therapy (BRAFi/MEKi). Nuclei are stained with DAPI (blue). Scale bar: 10 µm. (**G**) Quantification of translating *53BP1* mRNAs (spots) in the condition described in (**F**). Data shown represent the mean ± SEM of the number of spots/cells quantified in one independent experiment (see also Fig. EV2C for the other biological replicates) (unpaired Student’s *t*-test, ***p = 0.0001). (**H**) Representative confocal images of translating *53BP1* (magenta) and IQGAP*1* mRNAs (green) assessed by RiboMap assay in A375 cells surviving targeted therapy. Nuclei are stained with DAPI (blue). Scale bar: 10 µm. (**I**) Scatterplot illustrating the correlation between translating *53BP1* mRNAs and *IQGAP1* mRNAs in A375 drug-tolerant cells. Pearson correlation coefficient (r) and *p* value are reported. The data shown are the results of one biological replicate (see Fig. EV2G for the results obtained in other biological replicates). (**J**) Multiplex immunostaining (CODEX) showing the co-expression of 53BP1, CCSER2, HIPK2, IQGAP1, APC, and ANK2 in A375 cells. Scale bar 20 µm. (**K**) Heatmap generated from multiplexed imaging analysis, displaying, for each cell, single-cell mean intensity values (rows) for protein expression of selected differentially expressed markers (columns), regulated at either the translational or transcriptional level in control cells (DMSO) and drug-tolerant cells (BRAFi/MEKi). Source data are available online for this figure.

**Figure EV2:**
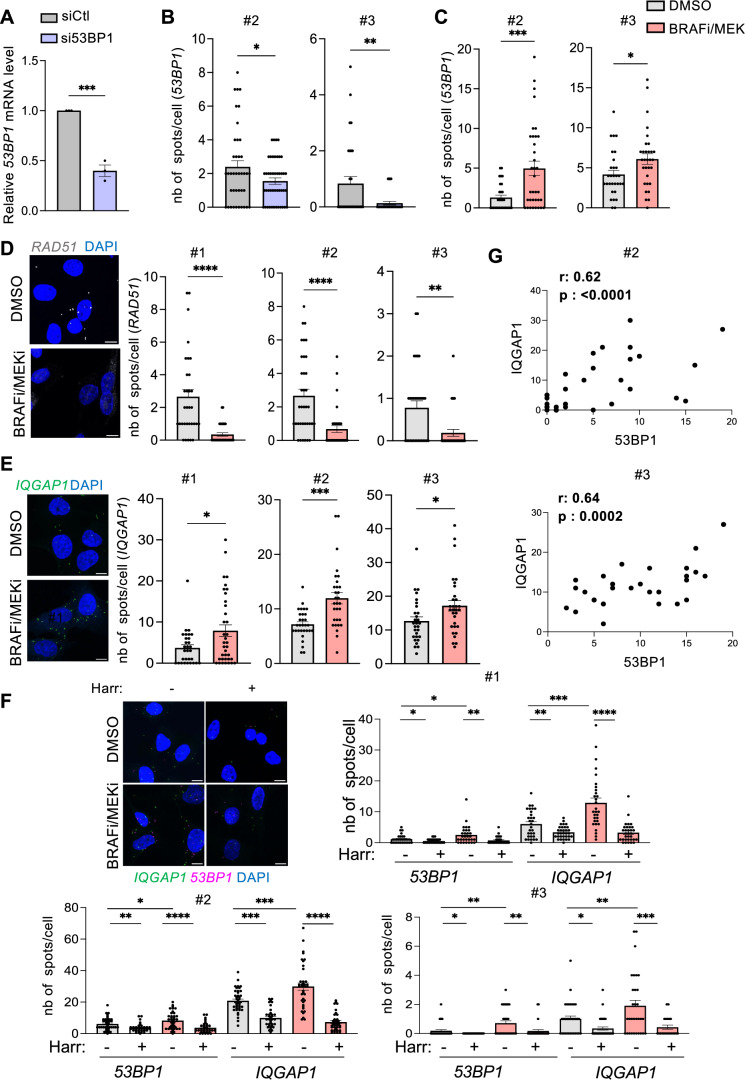
Single-cell analysis of translating mRNAs. (**A**) RT-qPCR quantification of *53BP1* mRNA level in A375 cells transfected with control siRNA (siCtl, 25 nM) or with siRNA targeting *53BP1* (si53BP1, 25 nM) corresponding to Fig. 2D,E. Data represent the mean ± SEM from three independent experiments (unpaired Student’s *t*-test, ***p = 0.0005). (**B**) Quantification of translating *53BP1* mRNAs (spots) in A375 cells transfected with siCtl or si53BP1. Data shown represent the mean ± SEM of the number of spots/cells quantified in the other two independent experiments (corresponding to Fig. 2E) (unpaired Student’s *t*-test,*p = 0.036, **p = 0.0097). (**C**) Quantification of translating *53BP1* mRNAs (spots) in A375 control cells (DMSO) or in cells surviving targeted therapy (BRAFi/MEKi). Data shown represent the mean ± SEM of the number of spots/cells quantified in other two independent experiments (corresponsing to Fig. 2G) (unpaired Student’s *t*-test, *p = 0.0265, ***p = 0.0006). (**D**) (left): Representative confocal images of translating *RAD51* mRNAs (gray) assessed by RiboMap assay in A375 control cells (DMSO) or in cells surviving targeted therapy (BRAFi/MEKi). Nuclei are stained with DAPI (blue). Scale bar: 10 µm. (right): Quantification of translating *RAD51* mRNAs (spots). Data represent the mean ± SEM of the number of spots/cells quantified in three independent experiments (unpaired Student’s *t*-test, #1, #2 ****p ≤ 0.0001, #3: **p = 0.0017). (**E**) (left): Representative confocal images of translating *IQGAP1* mRNAs (green) assessed by RiboMap assay in A375 control cells (DMSO) or in cells surviving targeted therapy (BRAFi/MEKi). Nuclei are stained with DAPI (blue). Scale bar: 10 µm. (right): Quantification of translating *IQGAP1* mRNAs (spots). Data represent the mean ± SEM of the number of spots/cells quantified in three independent experiments (unpaired Student’s *t*-test, #1: *p = 0.0175, #2: ***p = 0.0001, #3: *p = 0.0285). (**F**) (top): Representative confocal images of translating *53BP1* (magenta) and *IQGAP1* (green) mRNAs in DMSO and BRAFi/MEKi-treated cells in the presence or absence of Harringtonine (Harr) treatment (5 µM). Nuclei are stained with DAPI (blue). Scale bar: 10 µm. (bottom): Quantification of translating *53BP1* and *IQGAP1* mRNAs (spots). Data represent the mean ± SEM of the number of spots/cells quantified in three independent experiments (unpaired Student’s *t*-test, *p ≤ 0.05, **p ≤ 0.01,***p ≤ 0.001, ****p ≤ 0.0001). (**G**) Scatterplot illustrating the correlation between translating *53BP1* mRNAs and *IQGAP1* mRNAs found in the other two biological replicates in A375 drug-tolerant cells (corresponding to Fig. 2I). Pearson correlation coefficient (*r*) and *p* value are reported. Source data are available online for this figure.

**Figure EV3:**
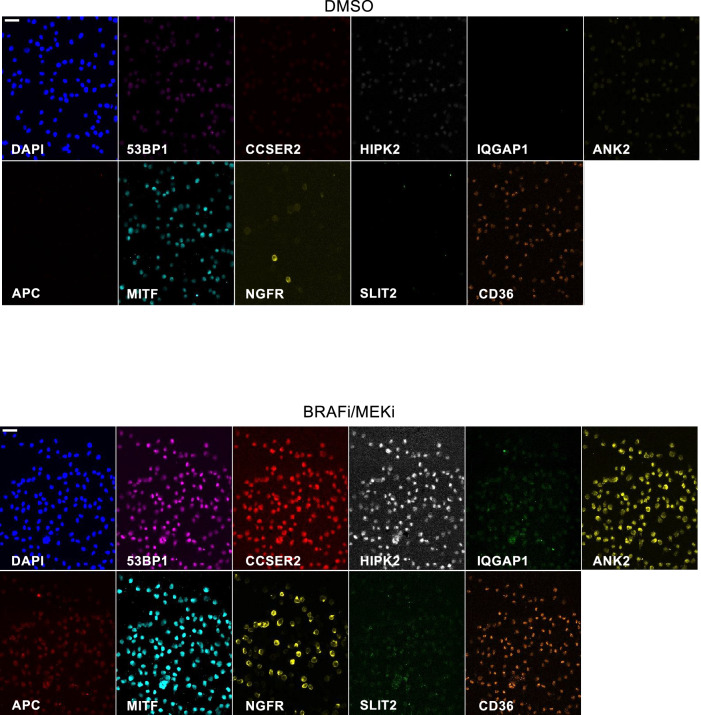
Single-cell multiplexed immunostaining of translationally and transcriptionally regulated proteins upon targeted therapy. Representative images of translationally regulated (53BP1, CCSER2, HIPK2, IQGAP1, ANK2, and APC) or transcriptionally regulated (MITF, NGFR, SLIT2, and CD36) protein markers analyzed by multiplex immunostaining (CODEX) in A375 control cells (DMSO) or in cells surviving 3 days of treatment with targeted therapy (BRAFi/MEKi). Scale bar: 50 µm. Source data are available online for this figure.

To next evaluate the effect of BRAFi/MEKi-targeted therapy on *53BP1* mRNA translation, we isolated actively translating ribosomes following a short L-azidohomoalanine (AHA) pulse and click chemistry, and their associated transcripts (Fig. 2A). A375 and M249 cells that survived 3 days of BRAFi/MEKi-targeted therapy exhibited increased association of *53BP1* mRNA with active ribosomes compared to untreated cells (Fig. 2B). The increase in *53BP1* mRNA translation mirrored the kinetic of protein expression observed in Fig. 1E, returning to levels observed in untreated cells after 9 days of drug withdrawal (Fig. 2B). Given the observed increase in 53BP1 protein levels in a subset of drug-tolerant cells in Fig. 1F, we intended to measure protein synthesis at a single cell level by using the recently described ribosome-bound mRNA mapping (RIBOmap) assay (Zeng et al, 2023). This approach detects ribosome-bound mRNAs through a proximity-based probe system (Fig. 2C). When in proximity, the three probes generate a DNA amplicon through rolling-circle amplification, indicative of active translation. To detect actively translated mRNAs, we used a fluorescent probe complementary to the DNA amplicon. Translating *53BP1* mRNAs decreased upon *53BP1* downregulation (Figs. 2D,E and EV2A,B), validating the specificity of the assay. We found that the increase of *53BP1* mRNAs association with active ribosomes occurred preferentially in a subset of drug-tolerant cells (Figs. 2F,G and EV2C). In contrast, the mRNA encoding the HRD factor RAD51 showed reduced association with active ribosomes (Fig. EV2D). Moreover, a subpopulation of drug-tolerant cells exhibited increased translation of *IQGAP1* mRNA, which we previously demonstrated to be another translationally regulated mRNA in drug-tolerant cells (Fig. EV2E) (Shen et al, 2019). The specificity of active translation detected by the RiboMap assay was further validated for both *53BP1* and *IQGAP1* mRNAs using the translation initiation inhibitor Harringtonine, which arrests ribosomes at the translation initiation sites (Ingolia et al, 2011). Harringtonine treatment led to a reduction in the number of actively translating *53BP1* and *IQGAP1* mRNAs in both control (DMSO-treated) cells and cells that survived BRAFi/MEKi treatment (Fig. EV2F), confirming that the RiboMap signal reflects actively translating mRNAs. Combined analysis of translating *53BP1* and *IQGAP1* mRNAs in A375 drug-tolerant cells using the RiboMap assay (Fig. 2H) revealed a significant correlation between the translation of *53BP1* and *IQGAP1* mRNAs (Figs. 2I and EV2G), with a Pearson correlation coefficient ranging from 0.62 to 0.8, suggesting a mechanism of translational co-regulation. Consistently, a single cell multiplexed imaging approach by co-detection by indexing (CODEX) indicated that a subpopulation of drug-tolerant cells exhibited the highest staining intensity for 53BP1, IQGAP1 and all four other selected markers (CCSER2, HIPK2, ANK2, and APC), whose expression was previously characterized as translationally regulated in drug-tolerant cells (Shen et al, 2019). These results suggest that BRAFi/MEKi induced selective translational regulation promotes their coordinated expression in this subset of cells. In contrast, such co-expression was not observed for other markers whose expression was shown to be induced at the transcriptional level (e.g., SLIT2, CD36) (Rambow et al, 2018) (Figs. 2J,K and EV3).

**Figure 3:**
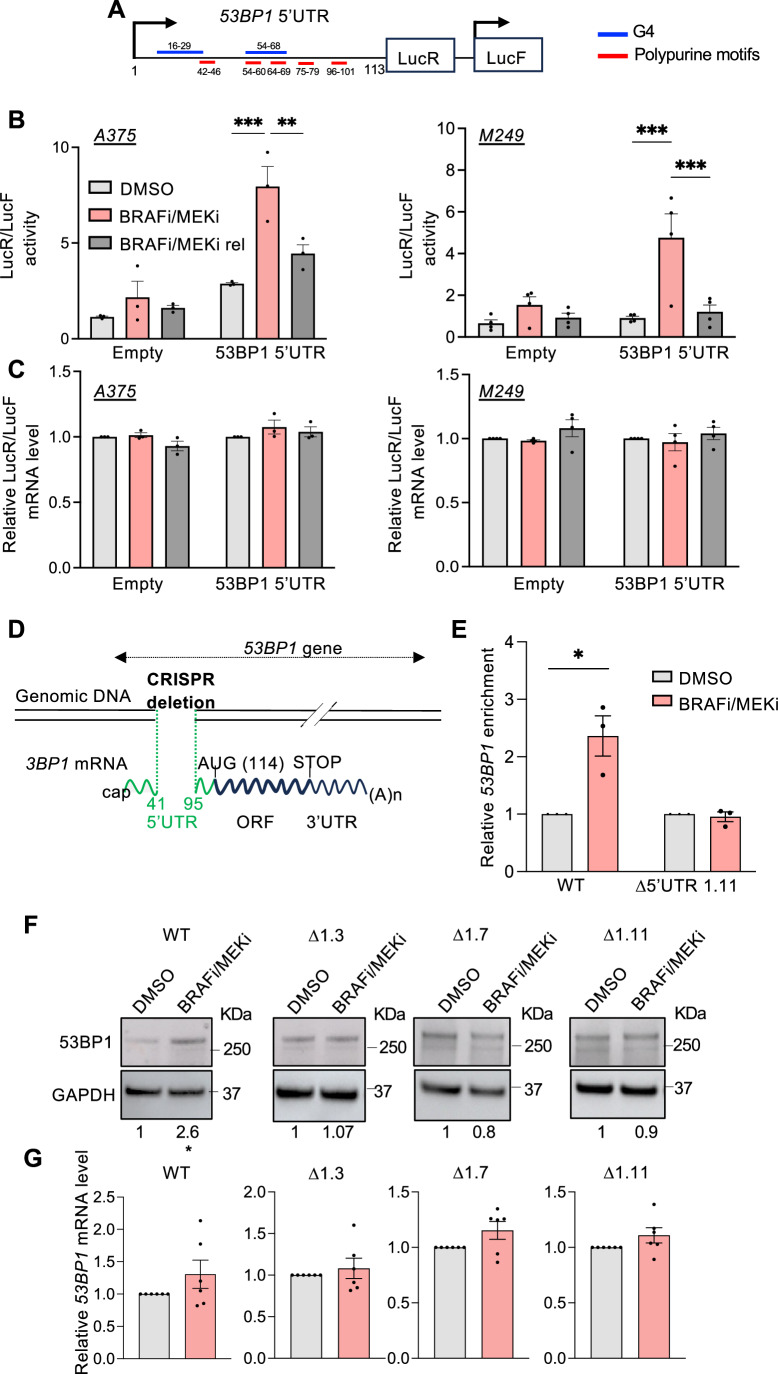
5’UTR-dependent *53BP1* mRNA translation is induced upon targeted therapy. (**A**) Schematic of the 53BP1 5′ UTR-containing luciferase reporter. (**B**) Quantification of the luciferase assay performed in A375 and M249 cells treated with DMSO or BRAFi/MEKi for 3 days, and in cells released from BRAFi/MEKi treatment (BRAFi/MEKi rel). Renilla (LucR) activity was measured 48 h after transfection, and the activity of the Firefly luciferase (LucF) was used as a control of transfection. The data shown represent the mean ± SEM from 3 (A375, two-way ANOVA ***p = 0.0001 for the comparaison BRAFi/MEKi vs DMSO and **p = 0.003 for the comparaison BRAFi/MEKi vs BRAFi/MEKi rel) or 4 (M249, two-way ANOVA ***p = 0.0002 for the comparaison BRAFi/MEKi vs DMSO and ***p = 0.0004 for the comparaison BRAFi/MEKi vs BRAFi/MEKi rel) independent experiments. (**C**) RT-qPCR quantification of the LucR/LucF mRNA level from the experiment in (**B**). (**D**) Schematic representation of genomic deletion by CRISPR/Cas9 genome editing leading to the deletion of 53BP1 5’UTR (from nucleotide 41 to 95) in A375 cells. (**E**) Quantification of *53BP1* mRNA enrichment in active ribosomes was performed using RT-qPCR in A375 WT cells and in A375 cells deleted for 53BP1 5’UTR (Δ5’UTR, clone 1.11). Control cells (DMSO) or drug-tolerant cells that survived targeted therapy (BRAFi/MEKi) were analyzed for *53BP1* mRNA enrichment, and data represent the mean ± SEM from three independent experiments (unpaired Student’s *t*-test, *p = 0.0180). (**F**) Western blot illustrating 53BP1 protein levels in A375 WT cells and A375 Δ5’UTR clones. For each cell line, control cells (DMSO) or drug-tolerant cells that survived 3 days of targeted therapy (BRAFi/MEKi) were analyzed for 53BP1 protein levels. GAPDH serves as a loading control, and the relative quantification from 4 independent experiments is indicated (unpaired Student’s *t*-test, WT *p = 0.0326; Δ1.3 *p* = 0.6292; Δ1.7 *p* = 0.1491; Δ1.11 *p* = 0.7708). (**G**) RT-qPCR quantification of *53BP1* mRNA level in A375 WT cells or in A375 Δ 5’UTR clones. For each cell line, control cells (DMSO) or drug-tolerant cells that survived 3 days of targeted therapy (BRAFi/MEKi) were analyzed for *53BP1* mRNA level. Data represent the mean ± SEM from six independent experiments (unpaired Student’s *t*-test, WT *p* = 0.1897; Δ1.3 *p* = 0.5183; Δ1.7 *p* = 0.0869; Δ1.11 *p* = 0.1430). Data were normalized using the geometric mean of TBP, Actin, and 18S housekeeping genes. Source data are available online for this figure.

Given the essential role of the 5’UTR in regulating translation of mRNAs (Hinnebusch et al, 2016), we next investigated the role of *53BP1* 5’UTR in regulating its translation. To this purpose, we used a luciferase expression reporter construct in which the *53BP1* 5’UTR was cloned upstream of the Renilla (LucR) open reading frame (Fig. 3A). Firefly expression—LucF—from the same plasmid served as a control for transfection/expression. We monitored the luciferase activities in transfected A375 and M249 cells upon BRAFi/MEKi treatment. We observed that BRAFi/MEKi induced an increase in the LucR/LucF activity ratio in cells transfected with the *53BP1* 5′ UTR-containing reporter upon BRAFi/MEKi treatment, which returned to the levels observed in untreated cells after 9 days of drug withdrawal (Fig. 3B). This increase in LucR activity upon BRAFi/MEKi reflected enhanced translation, as the LucR mRNA levels remained unchanged relative to the LucF mRNA (Fig. 3C). These data indicate that *53BP1* 5’UTR is sufficient to mediate the increase in *53BP1* mRNA translation upon BRAFi/MEKi treatment.

**Figure EV4:**
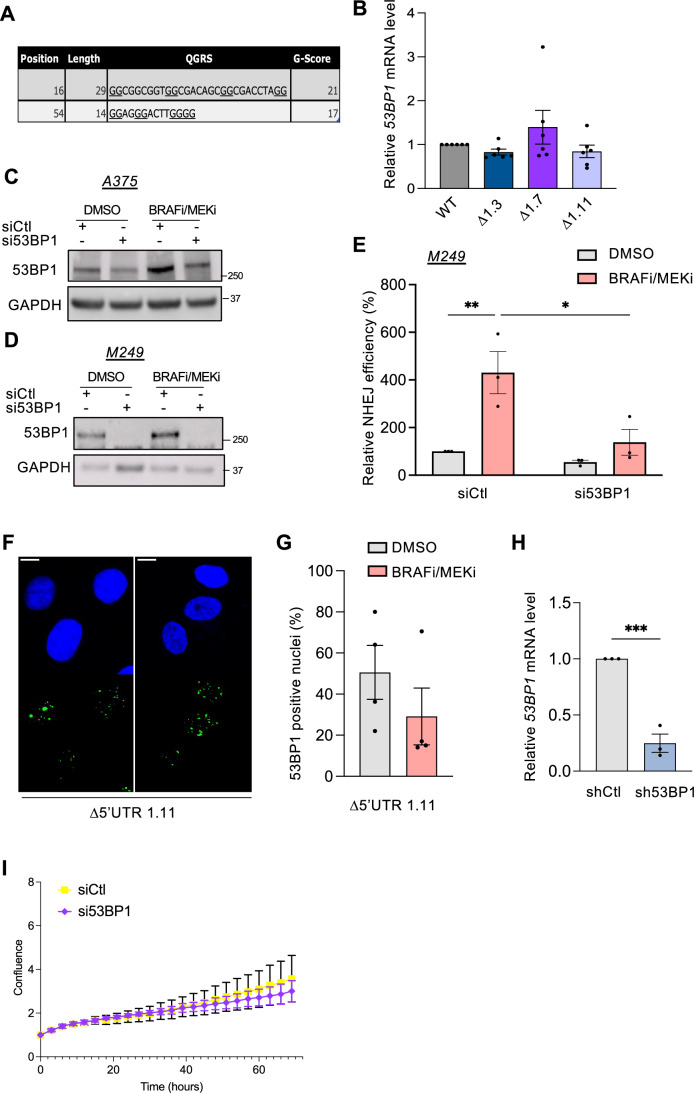
Characterization of *53BP1* 5'UTR and 53BP1-dependent phenotypes in drug-tolerant cells. (**A**) G-quadruplex in the 5’untranslated region of 53BP1 mRNA using QGRS Mapper. (**B**) RT-qPCR quantification of *53BP1* mRNA level in A375 WT cells or in A375 Δ 5’UTR clones. Data represent the mean ± SEM from six independent experiments. Data were normalized using the geometric mean of TBP, Actin, and 18S housekeeping genes. (**C**, **D**) Western blot illustrating 53BP1 protein levels in A375 and M249 control (DMSO) or drug-tolerant cells (BRAFi/MEKi) transfected with control siRNA (siCtl, 25 nM) or with siRNA targeting *53BP1* (si53BP1, 25 nM) corresponding to Figs. 4A and EV4E. GAPDH serves as a loading control. (**E**) Quantification of plasmid integration efficiencies of M249 control (DMSO) and BRAFi/MEKi drug-tolerant cells (BRAFi/MEKi) transfected with control siRNA (siCtl, 25 nM) or siRNA targeting *53BP1* (si53BP1, 25 nM). The mean ± SEM from three independent experiments is shown (two-way ANOVA, **p = 0.0088,*p = 0.0173). Data were normalized to M249 control cells transfected with siCtl, which were set to 100%. (**F**) Representative images of immunofluorescence in A375 Δ5’UTR cells (clone 1.11) treated with DMSO (control) or in A375 Δ5’UTR cells that survived BRAFi/MEKi-targeted therapy (BRAFi/MEKi), stained for 53BP1 (green) and nuclei (DAPI, blue). Scale bar, 10 μm. (**G**) Quantification of 53BP1 signal in the conditions described in F, analyzed by percentage of cells with ≥10 foci/nucleus. Data represent the mean ± SEM from four independent experiments. (**H**) RT-qPCR quantification of *53BP1* mRNA level in A375 cells stably expressing control shRNA, or shRNA targeting *53BP1*. Data represent the mean ± SEM from three independent experiments (unpaired Student’s *t*-test, ***p = 0.0008). (**I**) Proliferation of A375-R cells following siRNA-mediated depletion of 53BP1, assessed by IncuCyte live-cell imaging. Source data are available online for this figure.

The *53BP1* 5’UTR contains two predicted G-quadruplex (G4) secondary structures (Fig. EV4A). We next endogenously deleted a genomic region within the first exon of 53BP1 corresponding to nucleotides 41–95 of the 53BP1 5′ UTR in A375 cells (Δ5’UTR) (Fig. 3D). This region encompasses one of the predicted G4, thus potentially altering secondary structure within 53BP1 5′UTR. The 5’UTR deletion prevented the increase in *53BP1* mRNA translation in response to BRAFi/MEKi treatment, without affecting its translation in untreated cells (Fig. 3E). Analysis of *53BP1* transcript levels in three different clones indicated that the 5’UTR deletion did not affect promoter functions (Fig. EV4B) but abrogated the increase in 53BP1 protein levels observed upon BRAFi/MEKi (Fig. 3F) without changing *53BP1* mRNA levels (Fig. 3G). Altogether, these data show that the *53BP1* 5′ UTR is necessary to mediate the translational upregulation in drug-tolerant cells.

### *53BP1* mRNA translation promotes NHEJ upon targeted therapy

**Figure 4:**
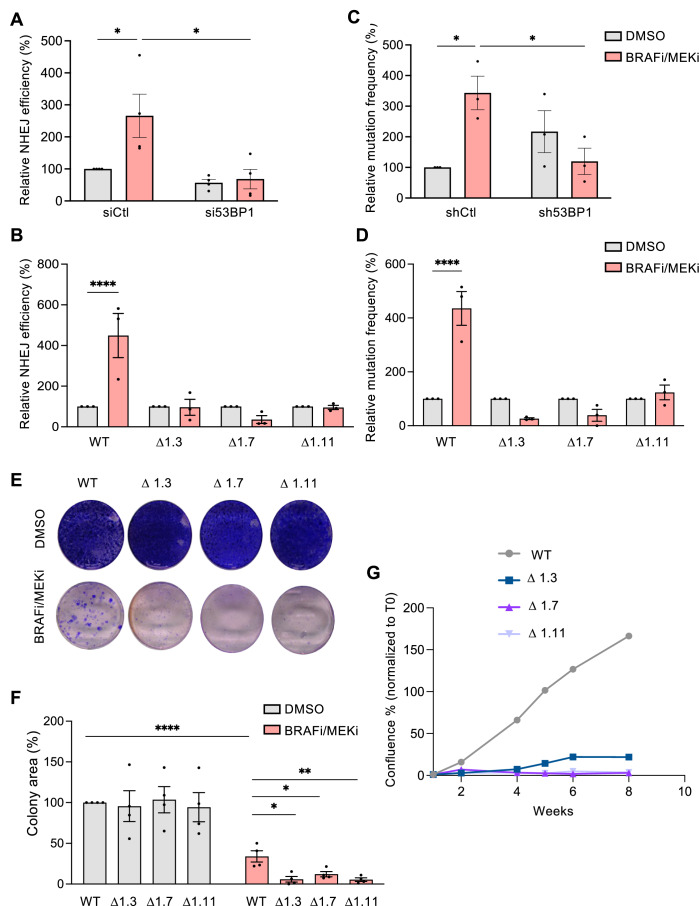
*53BP1* mRNA translation promotes NHEJ and mutability upon targeted therapy. (**A**) Quantification of plasmid integration efficiencies of A375 control (DMSO) and BRAFi/MEKi drug-tolerant cells (BRAFi/MEKi) transfected with control siRNA (siCtl, 25 nM) or siRNA targeting *53BP1* (si53BP1, 25 nM). The mean ±  SEM from four independent experiments is shown (two-way ANOVA, *p = 0.0381 for the comparison siCtl BRAFi/MEKi vs siCtl DMSO and *p = 0.0134 for the comparison siCtl BRAFi/MEKi vs si53BP1 BRAFi/MEKi). Data were normalized to A375 control cells transfected with siCtl, which were set to 100%. (**B**) Quantification of plasmid integration efficiencies of A375 WT cells and of 3 different A375 Δ 5’UTR clones following DMSO treatment or BRAFi/MEKi-targeted therapy. The mean ± SEM from three independent experiments is shown (two-way ANOVA, WT ****p ≤ 0.0001, Δ1.3 *p* = 0.9308; Δ1.7 *p* = 0.8640; Δ1.11 *p* = 0.9154). Data were normalized to cells treated with DMSO, which were set to 100%. (**C**) Relative HPRT mutation frequency for A375 cells stably expressing shCtl or sh53BP1, analysed in cells treated with DMSO or in BRAFi/MEKi drug-tolerant cells (BRAFi/MEKi). Data represent the mean ± SEM from three independent experiments (two-way ANOVA, *p = 0.0317 for the comparison shCtl BRAFi/MEKi vs shCtl DMSO and *p = 0.0472 for the comparison shCtl BRAFi/MEKi vs sh53BP1 BRAFi/MEKi) and are expressed as a fraction of the mutation frequency (%) observed in A375 cells expressing shCtl treated with DMSO. (**D**) Relative HPRT mutation frequency analyzed in control (DMSO) or drug-tolerant (BRAFi/MEKi) A375 WT cells or in three different A375 Δ 5’UTR clones. Data represent the mean ± SEM from three independent experiments (two-way ANOVA, WT **** *p* ≤ 0.0001, Δ1.3 *p* = 0.1844; Δ1.7 *p* = 0.5878; Δ1.11 *p* = 0.9399) and are expressed as a fraction of the mutation frequency (%) observed in control cells treated with DMSO. (**E**) Colony assay performed 3 weeks after continuous treatment of A375 WT or Δ5’UTR drug-tolerant cells (10,000 cells) with DMSO or BRAFi/MEKi (100 nM/ 10 nM). (**F**) Quantifications of the colony assay (mean ± SEM) from four independent experiments. *p* values were calculated by unpaired, two-tailed Student’s *t*-test WT DMSO vs WT BRAFi/MEKi ****p ≤ 0.0001, WT BRAFi/MEKi vs Δ1.3 BRAFi/MEKi *p = 0.0104, WT BRAFi/MEKi vs Δ1.7 BRAFi/MEKi *p = 0.0276, WT BRAFi/MEKi vs Δ1.11 BRAFi/MEKi **p = 0.0072). (**G**) Cell proliferation assay performed in A375 WT and Δ5’UTR drug-tolerant cells. Cells surviving BRAFi/MEKi treatment were plated, and cell proliferation was monitored under continuous BRAFi/MEKi (100 nM/10 nM) treatment for 8 weeks (*n* = 1 biological experiment). Source data are available online for this figure.

Since 53BP1 is a key promoter of NHEJ, we next investigated whether the observed  increase in *53BP1* mRNA translation in drug-tolerant cells could correlate with enhanced NHEJ. We first evaluated NHEJ activity of A375 and M249 cells by measuring random DNA integration into genomic DNA (Iiizumi et al, 2008; Rother et al, 2020). This assay measures the NHEJ-dependent genomic integration of a GFP-expressing linearized plasmid containing a Neomycin resistance cassette, and integration events are scored as the number of colonies resistant to G418 (Rother et al, 2020). A375 and M249 cells tolerant to BRAFi/MEKi treatment exhibited increased NHEJ efficiency compared to untreated controls, an effect that was abolished by siRNA-mediated depletion of 53BP1 (Figs. 4A and EV4C–E). To assess whether the NHEJ bias induced by targeted therapy was associated with increased *53BP1* mRNA translation, we evaluated NHEJ efficiency in three 5’UTR-deleted clones, where the targeted therapy-induced translation regulation of *53BP1* mRNA was compromised, as shown in Fig. 3.

Similar to the effects of siRNA-mediated depletion of 53BP1, the enhanced NHEJ efficiency observed in A375 cells upon targeted therapy was abrogated in the three tested 5’UTR-deleted clones surviving BRAFi/MEKi-targeted therapy (Fig. 4B). Accordingly, these clones showed no targeted therapy-induced increase in 53BP1-positive cells (Fig. EV4F,G). Consistent results were obtained across multiple clones, limiting potential off-target effects from CRISPR/Cas9-mediated deletion. These results indicate that the translational upregulation of *53BP1* induced by targeted therapy promotes NHEJ.

### *53BP1* mRNA translation promotes targeted therapy-induced mutability and the evolution towards acquired resistance

Recent studies showed that NHEJ-mediated repair plays a critical role in promoting genomic instability and the development of melanoma acquired resistance to targeted therapy (Dharanipragada et al, 2023). NHEJ-induced genomic instability may therefore increase the mutation rate in drug-tolerant cells, enhancing the likelihood of their evolution into genetically resistant clones (Cipponi et al, 2020; Russo et al, 2024; Russo et al, 2019). We thus investigated whether the 53BP1-mediated increase in NHEJ contributes to the induction of adaptive mutability in drug-tolerant melanoma cells. To this purpose, we analyzed the occurrence of genomic mutations upon BRAFi/MEKi by measuring loss-of-function mutations at the hypoxanthine-guanine phosphoribosyltransferase (HPRT) gene and consequent cell resistance to 6-thioguanine (Glaab et al, 1998; Miles and Hawkins, 2018). In line with the role of 53BP1, shRNA-mediated knockdown of 53BP1, significantly reduced the enhanced mutation frequency observed in non-targeting shRNA control cells following BRAFi/MEKi-targeted therapy (Figs. 4C and EV4H). To directly assess whether this increased mutability was driven by elevated *53BP1* mRNA translation, we evaluated mutation frequency at the *HPRT* locus in three different Δ5’UTR clones. In these clones, targeted therapy did not lead to an increase in mutation frequency compared to untreated cells (Fig. 4D). We next assessed whether the reduced mutability of drug-tolerant cells observed in Δ5’UTR clones was responsible for limiting the clonal capacity of drug-tolerant cells upon BRAFi/MEKi treatment. Compared to WT cells, drug-tolerant cells derived from Δ5′UTR clones exhibited a significant reduction in clonal emergence following continuous BRAFi/MEKi exposure for 3 weeks (Fig. 4E,F). By contrast, no differences in clonogenic capacity were observed between WT and Δ5′UTR cells under DMSO-treated control conditions. In line with this, monitoring of drug-tolerant cells proliferation over a two-month period revealed that drug-tolerant cells from Δ5′UTR clones displayed markedly impaired proliferative capacity compared to WT cells (Fig. 4G). Of note, stably resistant A375 cells (Shen et al, 2019) are insensitive to 53BP1 depletion, as assessed by IncuCyte-based live-cell proliferation assay (Fig. EV4I).

Together, these findings demonstrate that impairing *53BP1* translational regulation in drug-tolerant cells limits NHEJ capacity and the emergence of drug-tolerant-derived resistant and proliferative clones, thereby potentially overcoming acquired resistance.

### The use of the eIF4A inhibitor eFT226 prevents *53BP1* mRNA translation and adaptive mutability of drug-tolerant cells

**Figure EV5:**
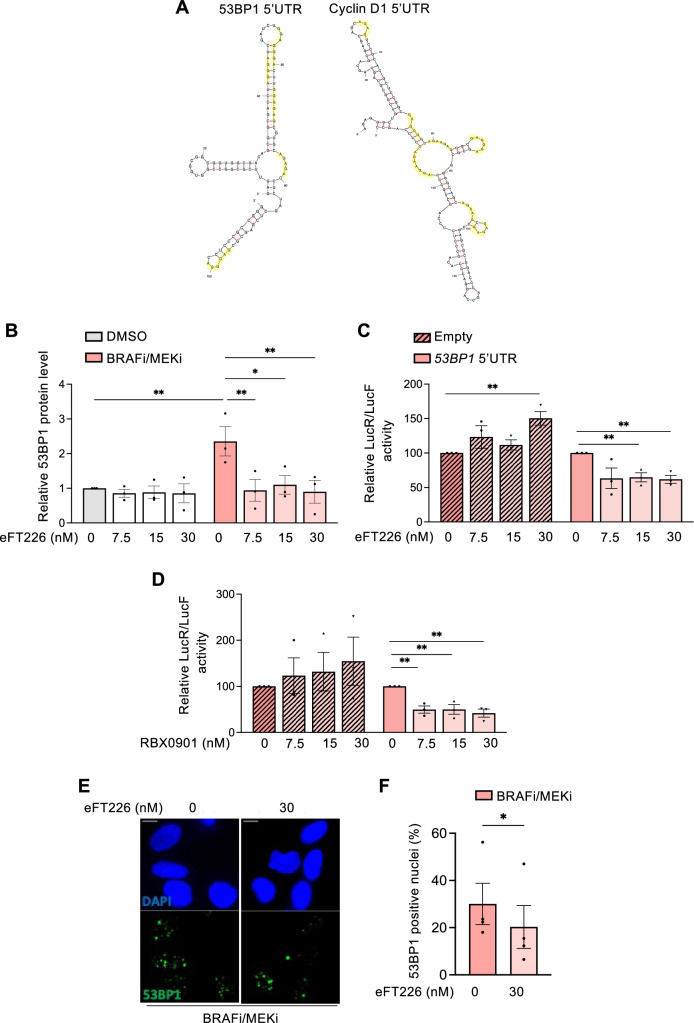
Pharmacological inhibition of 5'UTR-dependent *53BP1* translation following targeted therapy. (**A**) Representation of the 53BP1 and Cyclin D1 5’ UTRs using mfold software. Polypurine-rich sequences are highlighted in yellow. (**B**) Relative quantification of 53BP1 protein level in M249 control (DMSO) or drug-tolerant cells (BRAFi/MEKi), treated with the indicated concentration of eFT226 for 24 h. Data represent the mean ± SEM from three independent experiments (two-way ANOVA, BRAFi/MEKi: 0 vs 7.5 ** *p* = 0.0056, 0 vs 15 *p = 0.0135, 0 vs 30 **p = 0.0045). (**C**) Quantification of the luciferase assay performed in M249 drug-tolerant cells treated with the indicated concentrations of eFT226 for 12 h. Renilla (LucR) activity was measured at the end of the eFT226 treatment, and the activity of the Firefly luciferase (LucF) was used as a control of transfection. Data represent the effect of eFT226 on the luciferase activity of the reporter containing 53BP1 5’UTR or the Empty vector (without 53BP1 5’UTR) and are normalized to the eFT226 untreated conditions. The mean ± SEM from three independent experiments is reported (unpaired Student’s *t*-test, 53BP1: 0 vs 7.5 *p* = 0.0692, 0 vs 15 **p = 0.0060, 0 vs 30 **p = 0.0030). (**D**) Quantification of the luciferase assay performed in M249 drug-tolerant cells treated with the indicated concentrations of RBX0901 for 12 h. Data represent the effect of RBX0901 on the luciferase activity of the reporter containing 53BP1 5’UTR or the Empty vector (without 53BP1 5’UTR) and are normalized to the RBX0901 untreated conditions. The mean ± SEM from three independent experiments is reported (unpaired Student’s *t*-test, 53BP1: 0 vs 7.5 **p = 0.0029, 0 vs 15 **p = 0.0088, 0 vs 30 **p = 0.0027). (**E**) Representative images of immunofluorescence in A375 drug-tolerant cells that survived BRAFi/MEKi-targeted therapy (BRAFi/MEKi), treated with or without 30 nM eFT226 for 24 h and stained for 53BP1 (green) and nuclei (DAPI, blue). Scale bar, 10 μm. (**F**) Quantification of 53BP1 signal of the conditions in E, analyzed by percentage of cells with ≥10 foci/nucleus. Data represent the mean ± SEM from four independent experiments. *p* values were calculated by paired, two-tailed Student’s *t*-test (*p = 0.0387). Source data are available online for this figure.

Since we established a role for the *53BP1* 5′UTR in promoting increased mRNA translation in drug-tolerant cells, and given the previously demonstrated translational reprogramming in drug-tolerant cells mediated by the helicase eIF4A of the eIF4F complex (Shen et al, 2019), we sought to assess the clinical relevance of this translational regulation by targeting the eIF4A. To this end, we employed the clinically advanced, flavagline-derivative, small-molecule inhibitor eFT226 (Ernst et al, 2020). Similar to other flavaglines (Iwasaki et al, 2016; Iwasaki et al, 2019), eFT226 selectively represses the translation of mRNAs containing purine motifs in their 5′UTRs (Ernst et al, 2020). The 53BP1 5’UTR contains two predicted G-quadruplex (G4) secondary structures potentially sensitive to eIF4A inhibition (Wolfe et al, 2014), as well as several polypurine-rich sequences (Iwasaki et al, 2016) at a density comparable to those found in the 5’UTR of Cyclin D1, a well-established eIF4A-dependent mRNA (Figs. EV4A and EV5A).

**Figure 5:**
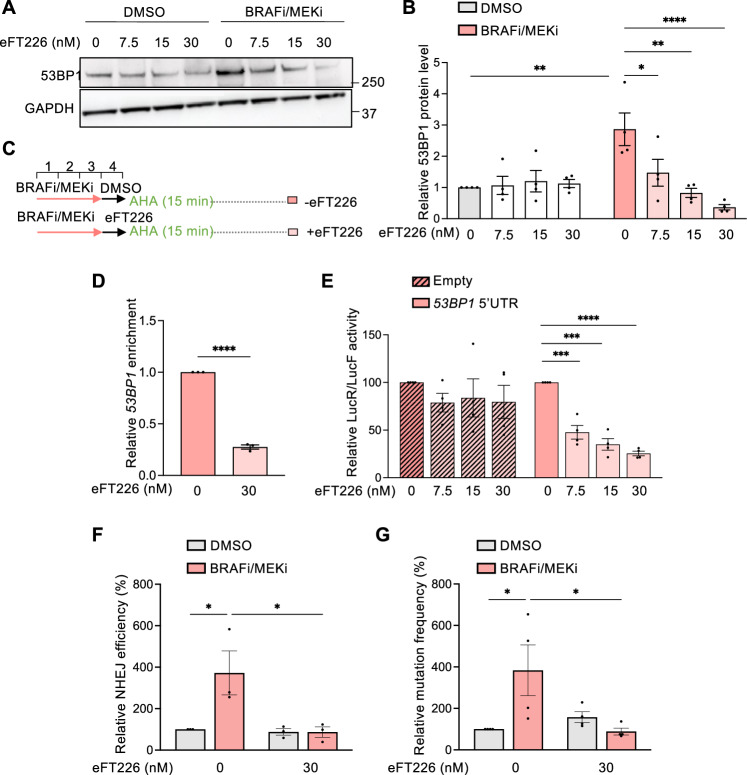
Targeting eIF4A with the use of eFT226 inhibits *53BP1* mRNA translation and decreases NHEJ and mutability upon targeted therapy. (**A**) Western blot illustrating 53BP1 protein levels in A375 control (DMSO) or drug-tolerant cells (BRAFi/MEKi), treated with the indicated concentration of eFT226 for 24 h. GAPDH serves as a loading control. (**B**) Relative quantification of 53BP1 protein level in A375 cells treated as in A. Data represent the mean ± SEM from four independent experiments (two-way ANOVA, 0 DMSO vs 0 BRAFi/MEKi **p = 0.0036, 0 BRAFi/MEKi vs 7.5 BRAFi/MEKi *p = 0.0490, 0 BRAFi/MEKi vs 15 BRAFi/MEKi **p = 0.0013, 0 BRAFi/MEKi vs 30 BRAFi/MEKi ****p < 0.0001). (**C**) Schematic representation of the protocol used for the isolation of actively translating ribosomes and their associated transcripts in A375 drug-tolerant cells (surviving 3 days BRAFi/MEKi therapy) treated with DMSO or 30 nM of eFT226 for 24 h. (**D**) Quantification of *53BP1* mRNA enrichment in active ribosomes was performed using RT-qPCR. Data represent the mean ± SEM from three independent experiments (unpaired Student’s *t*-test, ****p ≤ 0.0001). (**E**) Quantification of the luciferase assay performed in A375 drug-tolerant cells treated with the indicated concentrations of eFT226 for 12 h. Renilla (LucR) activity was measured at the end of the eFT226 treatment, and the activity of the Firefly luciferase (LucF) was used as a control of transfection. Data represent the effect of eFT226 on the luciferase activity of the reporter containing 53BP1 5’UTR or the Empty vector (without 53BP1 5’UTR) and are normalized to the eFT226 untreated conditions. The mean ± SEM from four independent experiments is reported (unpaired Student’s *t*-test, Empty: 0 vs 7.5 *p* = 0.0721, 0 vs 15 *p* = 0.4475, 0 vs 30 *p* = 0.2836, 53BP1 5’UTR: 0 vs 7.5 ***p = 0.0003, 0 vs 15 ***p = 0.0003, 0 vs 30 ****p ≤ 0.0001). (**F**) Quantification of plasmid integration efficiencies of A375 control (DMSO) and BRAFi/MEKi drug-tolerant cells treated with or without eFT226 (30 nM for 24 h). The mean ± SEM from three independent experiments is shown (two-way ANOVA, 0: DMSO vs BRAFi/MEKi *p = 0.0469, BRAFi/MEKi 0 vs 30 *p = 0.0369). Data were normalized to A375 control cells treated with DMSO, which were set to 100%. (**G**) Relative *HPRT* mutation frequency for A375 control cells treated with DMSO or BRAFi/MEKi drug-tolerant cells, treated with or without eFT226 (30 nM for 24 h). Data represent the mean ± SEM from four independent experiments (two-way ANOVA, 0: DMSO vs BRAFi/MEKi *p = 0.0347, BRAFi/MEKi 0 vs 30 *p = 0.0277) and are expressed as a fraction of the mutation frequency (%) observed in A375 control cells treated with DMSO. Source data are available online for this figure.

eFT226 specifically inhibited the increase in 53BP1 protein level observed in A375 and M249 drug-tolerant cells (Figs. 5A,B and EV5B). As assessed in A375 cells, this reduced 53BP1 protein expression in drug-tolerant cells corresponded to a reduced association of *53BP1* mRNA with active ribosomes following eFT226 treatment (Fig. 5C,D), indicating decreased translation. Consistently, in A375 and M249 drug-tolerant cells, eIF4A inhibition by eFT226 specifically decreased the translation of the *53BP1* 5′ UTR-containing reporter and had no such effects on the translation of a reporter mRNA containing no 5’UTR (“Empty”) (Figs. 5E and EV5C), indicating that the *53BP1* 5’UTR is sufficient to observe both the BRAFi/MEKi or the eFT226 effects on translation.

We next evaluated the NHEJ efficiency in A375 cells treated with eFT226 by measuring random DNA integration into genomic DNA (Iiizumi et al, 2008; Rother et al, 2020). eFT226 reduced the enhanced NHEJ efficiency observed in cells surviving BRAFi/MEKi-targeted therapy (Fig. 5F), which was associated with decreased 53BP1 nuclear recruitment (Fig. EV5E,F). The reduced NHEJ efficiency of drug-tolerant cells by eIF4A inhibition was accompanied by a decrease in the mutation frequency observed at the *HPRT* locus (Fig. 5G). These findings suggest that eIF4A inhibition reduces the BRAFi/MEKi-targeted therapy-induced adaptive mutability in A375 cells by impairing NHEJ, thereby potentially delaying the development of stable resistance to BRAFi/MEKi-targeted therapy.

### Combining BRAFi/MEKi-targeted therapy with small-molecule eIF4A inhibitors delays melanoma recurrence

**Figure EV6:**
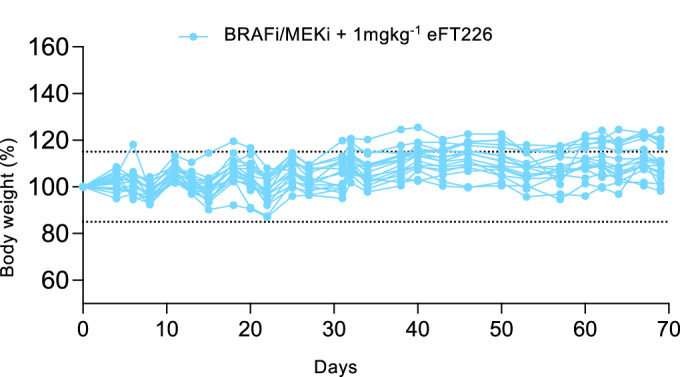
Tolerability of combined BRAFi/MEKi and eFT226 treatment in mice. Graph showing the percentage variation in body weight of mice after 70 days of treatment with PLX4720 (BRAFi) and PD0325901 (MEKi), combined with eFT226 (1 mg/kg). Source data are available online for this figure.

**Figure 6:**
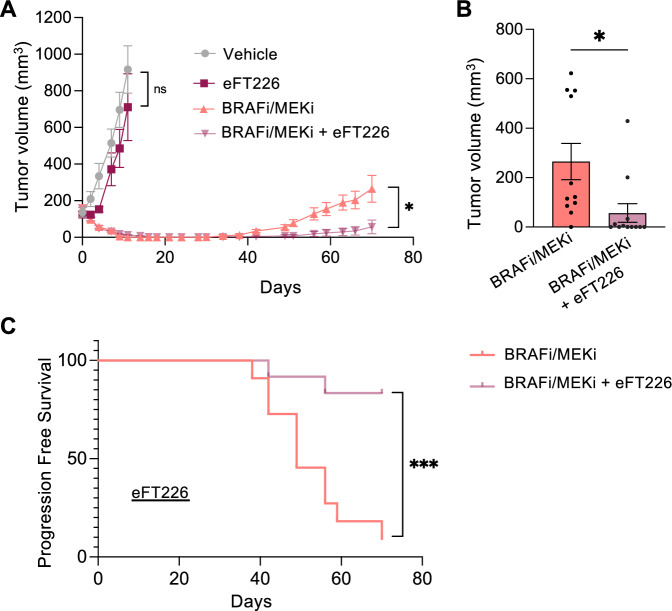
Targeting eIF4A with the use of eFT226 delays acquired resistance in vivo. (**A**) Growth curve illustrating the changes in tumor volume over 70 days in a human BRAF-mutant A375 melanoma xenograft model treated with single and combined agents as indicated (Vehicle, *n*= 5; eFT226, *n* = 5; BRAFi/MEKi, *n* = 11; BRAFi/MEKi + eFT226, *n* = 12). Graphs represent mean values ± SEM. *p* value was calculated by an unpaired, two-tailed Student’s *t*-test (*p = 0.0168). (**B**) Graphs representing the tumor volume (mean ± SEM) after 70 days of BRAFi/MEKi treatment (*n* = 11) or with BRAFi/MEKi +  eFT226 (*n* = 12). *p* value was calculated by an unpaired, two-tailed Student’s *t*-test (*p = 0.0168). (**C**) Kaplan–Meier curve for A375 melanoma xenograft treated with BRAFi/MEKi (*n* = 11), and BRAFi/MEKi + eFT226 (*n* = 12). Median time progression was 49 days for the BRAFi/MEKi group, and only 20% of mice belonging to the combination group relapsed after 70 days of treatment. Log rank (Mantel-Cox) for combination versus BRAFi/MEKi: *p* = 0.0004 (∗∗∗) and hazard ratio (log rank): 0.118 (95% CI, 0.03–0.3). Source data are available online for this figure.

To explore the therapeutic potential of targeting eIF4A-mediated *53BP1* mRNA translation in cancer cells transitioning from reversible non-genetic to irreversible genetic resistance to BRAFi/MEKi, we evaluated the efficacy of combining eFT226 with BRAFi/MEKi-targeted therapy. We used an A375 melanoma xenograft model in which tumors initially respond to BRAFi/MEKi therapy but eventually relapse (Hangauer et al, 2017). Nude-athymic mice with established A375 orthotopic tumors (>150 mm^3^) were fed with a control chow or a chow supplemented with PLX4720 (BRAFi) and PD0325901 (MEKi) and underwent intraperitoneal injection with or without 1 mg/kg of eFT226 twice a week. Notably, combinations were generally well tolerated for the duration of the study (Fig. EV6). While BRAFi/MEKi triggered potent tumor regression with a complete disappearance of the tumor after 10–11 days of treament, eFT226 was ineffective (Fig. 6A). The combination of eFT226 with BRAFi/MEKi-targeted therapy significantly delayed the development of resistance (Fig. 6A), with 10 out of 12 mice showing no relapse (tumor volume <50 mm³) at the end of the experiment (Fig. 6B). This was associated with a prolonged median progression-free survival (PFS) compared to BRAFi/MEKi treatment alone (Fig. 6C).

**Figure EV7:**
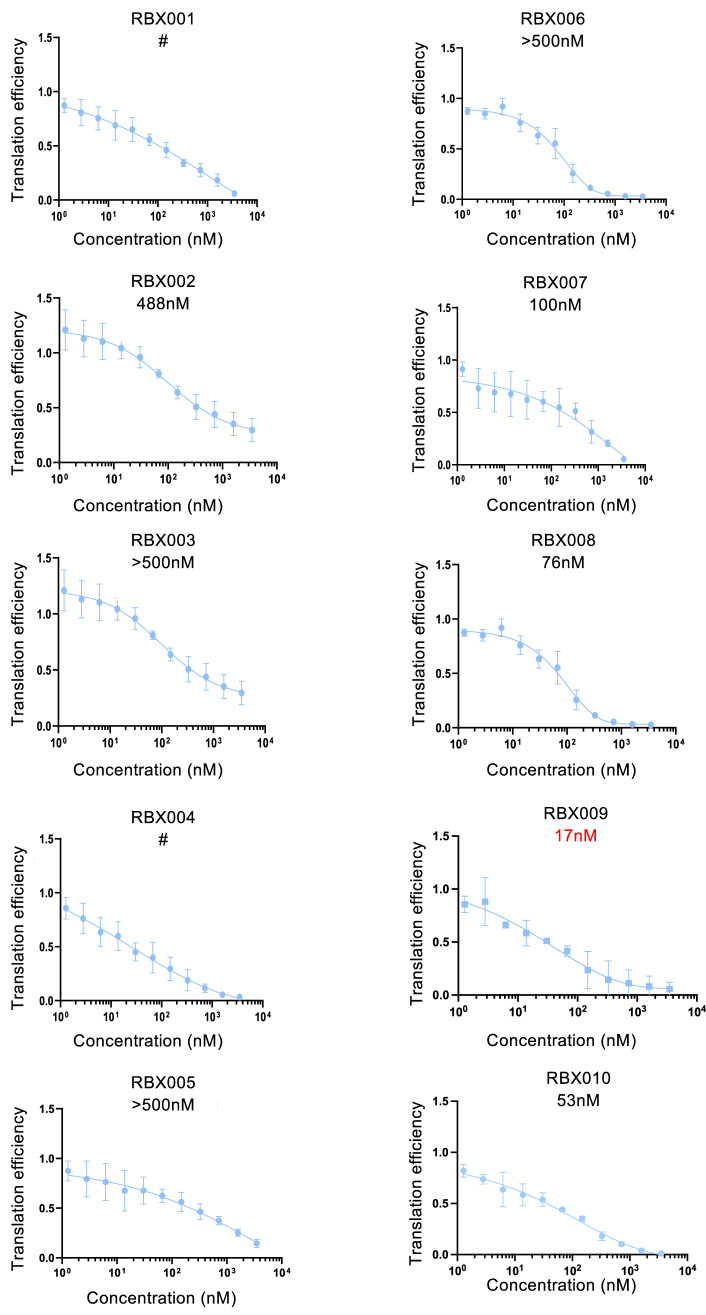

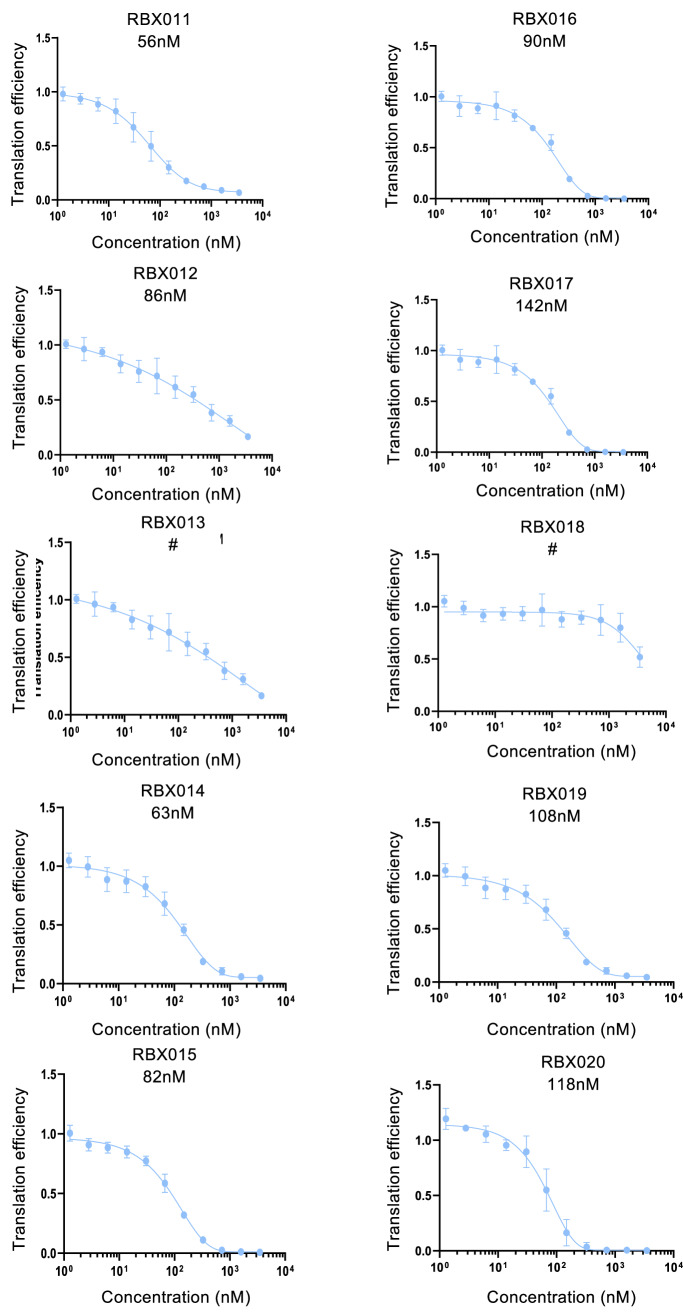

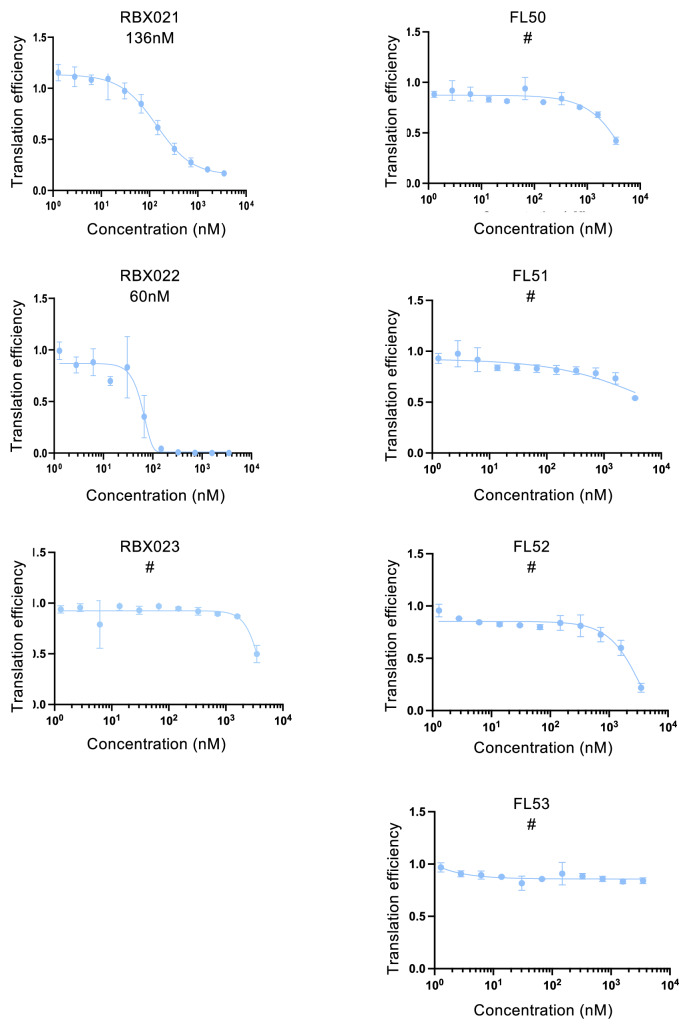
In vitro screening of flavagline derivatives. Screening of flavagline-derivative compounds was performed by assessing the in vitro translation of the (AG)₁₀ luciferase mRNA reporter in rabbit reticulocyte lysate treated with increasing concentrations of compounds for 1.5 h. Where applicable, the EC₅₀ (in nM) is indicated for each compound. Source data are available online for this figure.

**Figure 7:**
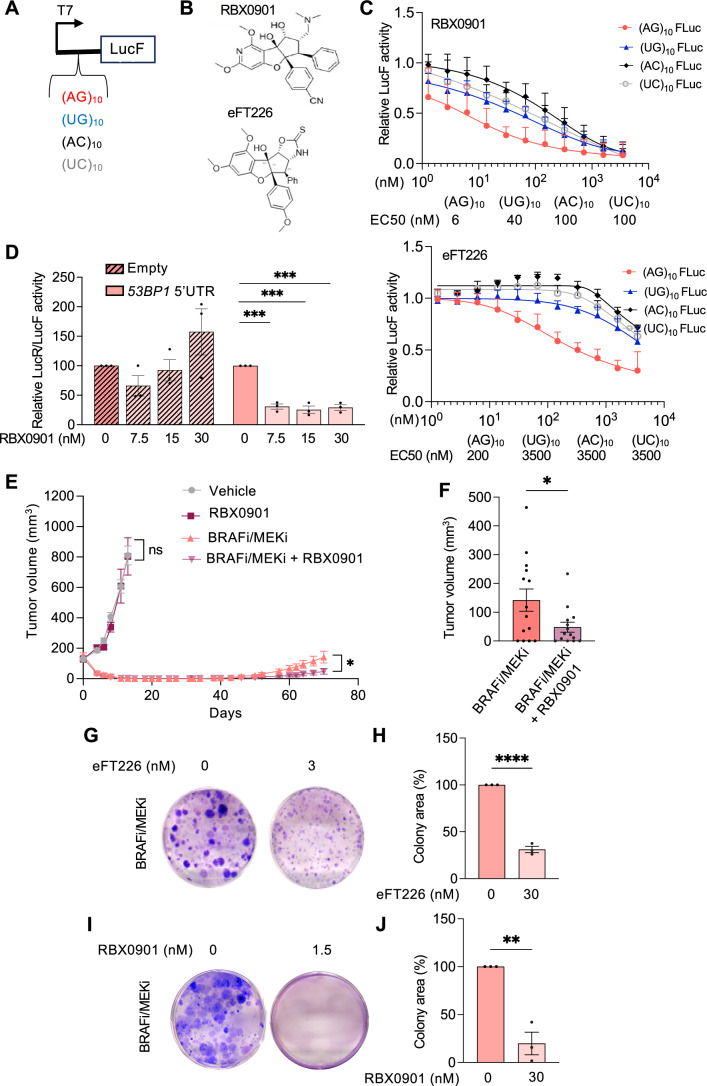
RBX0901, a novel flavagline-derivative, exhibits comparable efficacy to eFT226 in delaying acquired resistance*.* (**A**) Schematic of the luciferase reporters containing 5’UTRs with 10-mer sequence motif repeats used for in vitro translation. (**B**) Chemical structures of RBX0901 and eFT226. (**C**) Translation of luciferase mRNA reporter in rabbit reticulocyte lysate treated with increasing concentrations of eFT226 or RBX0901 for 1.5 h. EC50 (nM) for each reporter is indicated. Data represent the mean ±  SEM of four independent experiments. (**D**) Quantification of the luciferase assay performed with 53BP1 5’UTR or Empty (without 53BP1 5’UTR) reporters in A375 drug-tolerant cells treated with the indicated concentrations of RBX0901 for 12 h. Renilla (LucR) activity was measured at the end of drug treatment, and the activity of the Firefly luciferase (LucF) was used as a control of transfection. Data represent the effect of RBX0901 on the luciferase activity of the reporter containing 53BP1 5’UTR or the Empty vector and are normalized to the RBX0901 untreated conditions. The mean ± SEM from three independent experiments is reported (unpaired Student’s *t*-test, unpaired Student’s *t*-test, Empty: 0 vs 7.5 *p* = 0.1183, 0 vs 15 *p* = 0.6901, 0 vs 30 *p* = 0.2166, 53BP1 5’UTR: 0 vs 7.5 ***p = 0.0001, 0 vs 15 ***p = 0.0003, 0 vs 30 ***p = 0.0001). (**E**) Growth curve illustrating the changes in tumor volume over 70 days in a human BRAF-mutant A375 melanoma xenograft model treated with single and combined agents as indicated (Vehicle, *n* = 6; RBX0901, *n* = 6; BRAFi/MEKi, *n* = 14; BRAFi/MEKi + RBX0901, *n* = 14). Graphs represent mean values ± SEM. *p* value was calculated by unpaired, two-tailed Student’s *t*-test (*p = 0.0356). (**F**) Graphs representing the tumor volume (mean ± SEM) after 70 days of BRAFi/MEKi treatment (*n* = 14) or with BRAFi/MEKi + RBX0901 (*n* = 14). *p* value was calculated by unpaired, two-tailed Student’s *t*-test (*p = 0.0356). (**G**) Colony assay performed 3 weeks after incubating A375 cells, drug-tolerant cells (10,000 cells) with BRAFi/MEKi (100 nM/ 10 nM) in the presence or absence of eFT226 (3 nM). (**H**) Quantifications of the colony assay in (**G**) (mean ± SEM) from three independent experiments. *p* values were calculated by unpaired, two-tailed Student’s *t*-test (****p ≤ 0.0001). (**I**) Colony assay performed 3 weeks after incubating A375 cells, drug-tolerant cells (10,000 cells) with BRAFi/MEKi (100 nM/10 nM) in the presence or absence of RBX0901 (1.5 nM). (**J**) Quantifications of the colony assay in I (mean ± SEM) from three independent experiments. *p* values were calculated by unpaired, two-tailed Student’s *t*-test (**p = 0.0025). Source data are available online for this figure.

**Figure EV8:**
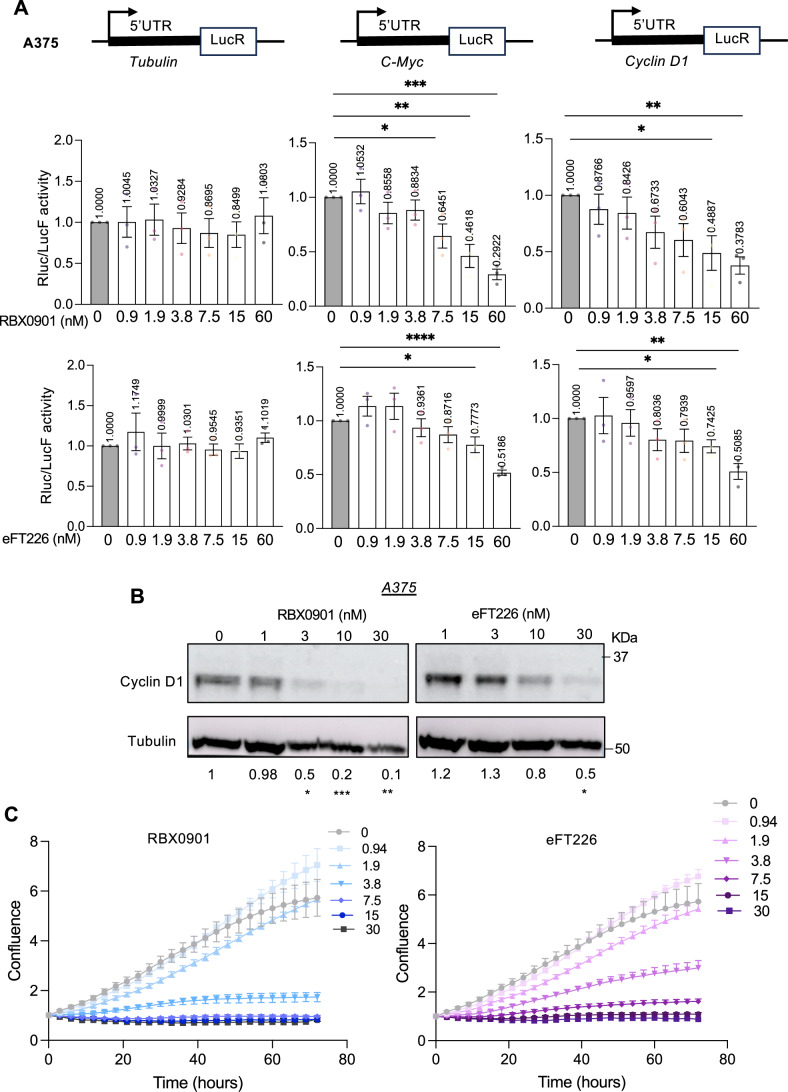
Comparison of eFT226 and RBX0901 effects on 5' UTR-dependent reporter translation inhibition and cell proliferation. (**A**) (top) Schematic representation of luciferase reporters containing the 5′UTRs of *Tubulin*, *c-MYC*, and *Cyclin D1*, used to monitor the effect of eIF4A inhibitors on reporter translation *in cellulo*. (bottom) Quantification of the luciferase assay performed in A375 cells treated with the indicated concentrations of RBX0901 or eFT226 for 24 h. Renilla (LucR) activity was measured 48 h after transfection, and the activity of the Firefly luciferase (LucF) was used as a control of transfection. Data were normalized to the untreated conditions and represent the mean ± SEM from three independent experiments (unpaired Student’s *t*-test, RBX0901: c-MYC: 0 vs 7.5 *p = 0.0324, 0 vs 15 **p = 0.0072, 0 vs 60 ***p = 0.000 1, Cyclin D1: 0 vs 15 *p = 0.0286, **p = 0.0025 eFT226: c-MYC: 0 vs 15 *p = 0.0383, ****p ≤ 0.0001, Cyclin D1: 0 vs 15 *p = 0.0133, **p = 0.0012). (**B**) Western blot illustrating Cyclin D1 protein levels in A375 cells treated with the indicated concentration of RBX0901 or eFT226 for 24 h. Tubulin serves as a loading control, and the quantification relative to untreated conditions from three independent experiments is indicated. *p* values were calculated by unpaired, two-tailed Student’s *t*-test (RBX0901: 0 vs 3 *p = 0.0498, 0 vs 10 ***p = 0.0001, 0 vs 30 **p = 0.0012; eFT226: 0 vs 30 *p = 0.0376). (**C**) Cell proliferation assay performed in A375 cells treated with the indicated concentrations of RBX0901 or eFT226 for 72 h. Data represent the mean ± SEM from three independent experiments. Source data are available online for this figure.

**Figure EV9:**
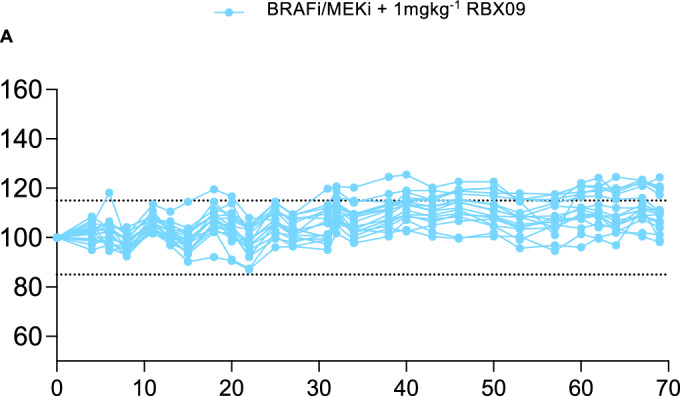
Tolerability of combined BRAFi/MEKi and RBX0901 treatment in mice. Graph showing the percentage variation in body weight of mice after 70 days of treatment with PLX4720 (BRAFi) and PD0325901 (MEKi), combined with RBX0901 (1 mg/kg). Source data are available online for this figure.

In addition to eFT226, we chose to evaluate a series of synthetic flavagline derivatives for their capacity to inhibit the translation of luciferase reporter mRNA containing polypurine (AG)_10_ motif repeats within the 5’UTR, using an in vitro rabbit reticulocyte lysate system (Fig. EV7, see Methods). Among the compounds screened, RBX09 exhibited the strongest inhibitory activity (Fig. EV7). RBX09 was generated by introducing an oxazolidinethione moiety fused to the flavagline scaffold (Methods). Given the promising activity of racemic RBX09, we prepared its pure enantiomer RBX0901 (Methods), which demonstrated greater effects than eFT226, with an ~100-fold improvement in EC₅₀, in inhibiting the translation of mRNA reporters containing AG-rich 5′UTRs compared to reporters with (UC)-rich or (AC)-rich or (UG)-rich 5′UTRs (Fig. 7A–C). As for eFT226, RBX0901 efficiently decreased the translation of the *53BP1* 5’UTR containing reporter in drug-tolerant cells, with no significant effect on the translation of the Renilla reporter containing no 5’UTR “Empty” (Figs. 7D and EV5D). Additionally, RBX0901 tended to be more effective than eFT226 in reducing the translation of reporters containing the 5′UTRs of c-Myc and Cyclin D1, whose translation have previously been shown to be eIF4F-dependent (Gerson-Gurwitz et al, 2021; Lin et al, 2008), while having no impact on the translation of the reporter containing the 5’UTR of the housekeeping gene Tubulin (Fig. EV8A). In line with this, RBX0901 had markedly reduced Cyclin D1 protein levels in A375 cells (Fig. EV8B). Assessment of cytostatic activity revealed that both compounds exhibited similar efficacy in inhibiting A375 cell proliferation, resulting in comparable IC₅₀ values (eFT226: 3.7 nM; RBX09: 2.6 nM) (Fig. EV8C). Combining BRAFi/MEKi with RBX0901, administrated at a non-toxic dose of 1 mg/Kg twice a week (as for eFT226) (Fig. EV9), significantly delayed tumor relapse, with 9 out of 14 mice remaining relapse-free (Fig. 7E,F). These findings demonstrate that co-treatment with BRAFi/MEKi and eIF4A inhibitors can effectively delay tumor relapse. Consistently, combining BRAFi/MEKi-targeted therapy with eFT226 or RBX0901 also markedly reduced the emergence of drug-tolerant-derived proliferative clones in vitro (Fig. 7G–J).

## Discussion

Here we report that eIF4A-mediated translation of the mRNA encoding the key error-prone NHEJ mediator 53BP1 mediates drug-induced mutability, driving melanoma cells' evolution towards acquired resistance. Our findings reveal how the dynamic translation regulation of gene expression in response to DNA-damaging targeted therapy may facilitate melanoma cells' adaptation to the DNA insult, while concomitantly and inadvertently enhancing the mutation rate of cancer cells through the promotion of the NHEJ pathway.

NHEJ pathway of DNA repair has been described as a survival strategy exploited by various cancers to withstand radio- or chemotherapy (Goodwin et al, 2013; Jun et al, 2016; Mi et al, 2025; Willoughby et al, 2020), with increased levels of key NHEJ components, including 53BP1, being correlated with therapeutic failure and cancer progression (Beskow et al, 2009; Bonanno et al, 2013; Bouchaert et al, 2012; Lai et al, 2010). NHEJ can compensate for defects in HDR (Lord and Ashworth, 2016), a vulnerability that underpins the clinical efficacy of poly(ADP-ribose) polymerase (PARP) inhibitors in the treatment of BRCA1/2-mutated cancers (Bouwman et al, 2010; Patel et al, 2011). Our findings, along with those of others, indicate that treatment with BRAFi/MEKi induces a BRCAness phenotype, characterized by the downregulation of key HDR components and a resulting impairment in HDR proficiency. Notably, in melanoma and other cancers subjected to targeted therapies, this repression has been shown to occur at the transcriptional level, at least for specific HDR factors such as RAD51 and BRCA1/2, and to be independent of cell cycle distribution (Cipponi et al, 2020; Maertens et al, 2019; Makino et al, 2020; Russo et al, 2019). Nonetheless, a recent study identified RNA-binding proteins (RBPs) as contributors to HDR deficiency across multiple cancers (McGrail et al, 2023), highlighting a possible role of post-transcriptional mechanisms in inhibiting the expression of HDR components, which remains yet to be tested.

In the context of HDR defects, our results demonstrate that drug-tolerant melanoma cells become increasingly reliant on the NHEJ pathway for DNA repair, a shift associated with elevated mutation frequency. While we used the *HPRT* locus as a proxy for global genomic mutability, our findings are consistent with recent results in colorectal cancer, where mathematical modeling and a modified Luria–Delbrück fluctuation assay indicated that targeted therapies are associated with an increased mutation rate (Russo et al, 2022). This shift away from high-fidelity DNA repair has been proposed to contribute to the genomic instability observed in cancer cells exposed to nongenotoxic treatments (Cipponi et al, 2020). While we cannot exclude the contribution of HDR defects to the observed mutation rate, our data support prior studies implicating NHEJ activity in genomic instability and acquired resistance of melanoma to targeted therapies (Dharanipragada et al, 2023).

Beyond transcriptional regulation, our study connects therapy-induced translational reprogramming to NHEJ efficiency and therapy-induced mutability. We identify eIF4A as a key regulator of *53BP1* mRNA translation, implicating the eIF4F complex in this process. Supporting this, *53BP1* mRNA translation was previously shown to be regulated by eIF4G1 in breast cancer cells following DNA damage (Badura et al, 2012), further confirming its reliance on eIF4F complex components. The translational control of 53BP1 by the eIF4F complex may thus be conserved across different cancer types and stress responses. Since the eIF4F complex is located at the convergence of several cell signaling pathway (Malka-Mahieu et al, 2017), including the PI(3)K/AKT/mTOR pathway and the RAS/RAF/MEK/ERK/MNK MAPK pathway, it is possible that the translation of the *53BP1* mRNA is regulated in cells tolerant to the various targeted therapies developed against these main signaling pathways in different cancer types. Nevertheless, eIF4F-independent functions of eIF4A have also been reported (Brito Querido et al, 2024), and the precise mechanism by which eIF4A regulates *53BP1* translation remains to be elucidated. Future studies aimed at identifying additional trans-acting factors that cooperate with eIF4A in driving 5′UTR-dependent translation of *53BP1* will help clarify this mechanism.

While eIF4A inhibition has pleiotropic effects, our use of 5’UTR deletion provides evidence that the 53BP1-NHEJ axis is a critical mediator of the adaptive mutability of drug-tolerant cells, as shown in Fig. 4 More specifically, experiments involving CRISPR/Cas9-mediated deletion of the *53BP1* 5’UTR directly show that the translational upregulation of *53BP1* is not just correlated with, but is necessary and sufficient for, the observed increase in NHEJ activity. This provides a direct causal link between a specific translational event and the subsequent shift in DNA repair pathway choice. Indeed, although NHEJ, together with 53BP1 nuclear recruitment, is predominant during the G0/G1 phase of the cell cycle (Hustedt and Durocher, 2016; Lieber et al, 2003; Panier and Boulton, 2014), and although BRAFi/MEKi-targeted therapy has been shown to promote G0/G1 cell-cycle arrest in the majority of the cell population (Fallahi-Sichani et al, 2017; Villanueva et al, 2010), our findings suggest that a direct mechanism of translational regulation, rather than an indirect effect of cell-cycle phase, is responsible for the observed shift in NHEJ. Moreover, this shift enhances genomic instability and the evolution towards acquired resistance. A detailed study will nevertheless be necessary to identify the *cis*-acting elements present in the 53BP1 5’UTR. We previously demonstrated that increased *N*6-methyladenosine modification in the 5’UTR of a subgroup of mRNAs promoted enhanced translation in melanoma drug-tolerant cells (Shen et al, 2019), suggesting a possible mechanism for 53BP1 translational regulation.

Previous work has shown that the selective translation of DNA repair factors in response to genotoxic stress can be driven by structures or sequence elements within UTRs, often through cap-independent mechanisms (Spriggs et al, 2010). Although our previous genome-wide analysis of differentially translated mRNAs in this context did not identify other NHEJ factors, it will be important to investigate whether additional NHEJ proteins upregulated during targeted therapy are subject to similar translational regulation. Notably, LIG4 has recently been reported to undergo translational regulation following genotoxic stress (Mohanan et al, 2025).

Importantly, our data show that increased translation of *53BP1* alone is sufficient to drive the observed increase in mutation frequency in drug-tolerant cells. We propose and validate a therapeutic strategy to counteract this resistance mechanism by inhibiting eIF4A. Using two different small-molecule inhibitors, including a newly developed one, we show that co-treatment with BRAF/MEK inhibitors significantly delays tumor relapse in xenograft models. The in vivo efficacy of eIF4A inhibition likely represents a composite effect derived from the translational suppression of numerous pro-survival pathways, and the precise contribution of the 53BP1-mutability axis relative to these other effects has not been quantitatively determined. However, DNA repair genes appear to exhibit eIF4A dependency in multiple cancer models (Lehman et al, 2022; Müller et al, 2019), and the effect of eIF4A inhibition on NHEJ was phenocopied by selective targeting of *53BP1* mRNA translation, suggesting that this axis is, at least in part, a key mediator of the response.

Finally, previous work demonstrated that coordinated inhibition of HDR and NHEJ factors in melanoma leads to synthetic lethality triggered by unresolved DNA damage (Maertens et al, 2019). In this work, we propose that inhibiting 53BP1-dependent NHEJ hinders the selection of new, fitter phenotypes driven by increased genetic instability that are drivers of acquired resistance to targeted therapy, pointing to a new role of mRNA translation in acquired mutability of drug-tolerant cells.

## Methods

**Table:** Reagents and tools table

Reagent/resource	Reference or source	Identifier or catalog number
**Experimental models**		
Hsd: Athymic Nude-Foxn1nu	Envigo	ENV:HSD-069
Human: A375	ATCC	RRID:CVCL_0132
Human: M249	Thomas Graeber’s laboratory, USA	RRID:CVCL_D755
Human: A375_R cell line	Shen et al, 2019	N/A
Human: A375_shCtl/sh53BP1	This study	N/A
Human: A375_Δ5’UTR	This study	N/A
Human: HeLa	ATCC	RRID:CVCL_0030
**Recombinant DNA**		
MISSION® pLKO.1-puro Non-Target shRNA Control Plasmid DNA	Sigma-Aldrich	SHC016-1EA
MISSION® pLKO.1-puro 53BP1 shRNA Plasmid DNA	Sigma-Aldrich	TRCN0000018866
psiCHECK™-2 Vector	Promega	Cat# C8021
pEGFP-C1 plasmid	Van Attikum’s lab, The Netherlands	N/A
VSVG	Addgene	#14888
psPax2	Addgene	#12260
**Antibodies**		
53BP1	Bethyl laboratories	A300-272A
53BP1	Cell Signaling	88439
53BP1	Novusbiologicals	NB100-304
gH2AX	Abcam	ab26350
RAD51	Abcam	ab176458
LIG3	Abcam	ab96576
XRCC4	Abcam	ab97351
KU70	Abcam	ab3114
GAPDH	Sigma-Aldrich	G9545
Cyclin D1	Sigma-Aldrich	C7464
Tubulin	GeneTex	GTX628802
CCSER2	Novusbiologicals	NBP2-84905
HIPK2	Sigma-Aldrich	AV32586
IQGAP1	Invitrogen™	33-8900
ANK2	CreativeDiagnostics	DCABH-2533
APC	Abcam	ab239828
MITF	Abcam	ab182842
NGFR	BioLegend	BLE345102
SLIT2	Abcam	ab242404
CD36	BioLegend	BLE336202
anti-Rabbit IgG Antibody, Alexa Fluor™ 488	Invitrogen™	A21206
Goat anti-Mouse IgG (H + L) Secondary Antibody, HRP	Invitrogen™	31430
Goat anti-Rabbit IgG (H + L) Secondary Antibody, HRP	Invitrogen™	31460
**Oligonucleotides and other sequence-based reagents**		
Listed in Table EV2		
**Chemicals, enzymes and other reagents**		
Dulbecco’s Modified Eagle Medium (DMEM) high glucose	Biowest	#L0101-500
RPMI-1640	Biowest	#L0500
Fetal Bovine Serum (FBS)	Thermo Fisher Scientific	A5256701
Penicillin-Streptomycin	Thermo Fisher Scientific	15140163
L-Glutamine (200 mM)	Thermo Fisher Scientific	A2916801
Trypsin-EDTA (0.25%), phenol red.	Thermo Fisher Scientific	25200056
Lipofectamine RNAiMAX (InvitrogenTM,	Invitrogen™	#13778150
Lipofectamine LTX Reagent	Invitrogen™	#15338100
Lipofectamine 2000	Invitrogen™	#11668019
DMSO	Sigma-Aldrich	34943-M
SybrSafe DNA Gel Stain	Invitrogen™	S33102
Crystal Violet Solution	Sigma-Aldrich	C0775
DAPI	MERCK LIFE SCIENCE SRL	5087410001
ProLong™ Gold Antifade Mountant	Invitrogen™	#P36930
ProLong™ Diamond with DAPI	Invitrogen™	#P36971
bovine serum albumin	Sigma-Aldrich	#A9576
Tween® 20	Sigma-Aldrich	#P6585
AHARIBO RNA	Immagina Biotechnology	#AHA-RM12,
mMESSAGE mMACHINE™ T7 Transcription Kit	Invitrogen™	#AM1344
Rabbit Reticulocyte Lysate System	Promega	Cat. No. L4960
Dual-Luciferase Reporter Assay System	Promega	#E1910
UltraView Universal DAB Detection Kit	Roche	Cat# 760-500
Vemurafenib	Selleckchem	S1267
Cobimetinib	MedChemExpress	HY-13064
eFT226	MedChemExpress	HY-112163
6-Thioguanine	Sigma-Aldrich	A4882
G418	Sigma-Aldrich	G8168
Bleomycin	Selleckchem	S1214
Harringtonine	MedChemExpress	HY-N0862
Cycloheximide solution	Merck	C4859
Master Mix PCR Power SYBR™ Green	Thermo Fisher Scientific	A25778
Kit TURBO DNA-free™	Thermo Fisher Scientific	AM1907
TRIzol™	Thermo Fisher Scientific	15596018
Random Hexamer Primer	Thermo Fisher Scientific	S0142
SuperScript III™	Thermo Fisher Scientific	18080085
RNaseOUT™	Thermo Fisher Scientific	10777019
BCA Protein Assay Kit	Thermo Fisher Scientific	23225
Quick Start Bovine Serum Albumin Standard Set	Thermo Fisher Scientific	5000207
**Software**		
ImageJ/Fiji	NIH	RRID:SCR_003070
GraphPad Prism	GraphPad Prism	RRID:SCR_002798
QuPath	Bankhead et al, 2017	RRID:SCR_018257
Cellpose	Stringer et al, 2021	RRID:SCR_021716
Incucyte® Cell-by-Cell Analysis Software Module	Sartorius	9600-0031
CFX Maestro™ Software	Bio-Rad	#12004110,
CODEX® Instrument Manager, CIM	Akoya Bioscience	
myProMS-Quant v3.10]		
**Other**		
Bio-Rad CFX96 instrument	Bio-Rad	RRID:SCR_018064
Keyence BZ-X800 All-in-One Fluorescence Microscope	Keyence	RRID:SCR_023617
Tristar 2 Multimode	Berthold	56550PR2
Vanquish Neo nanoLC system	Thermo Fisher Scientific	Cat# VN-S10-A-01
SP8X confocal microscope	Leica Microsystems	
Fusion FX imaging system	Vilber	

### Patient samples

Metastatic melanoma patients treated with dabrafenib or dabrafenib + trametinib at Gustave Roussy Cancer Campus (Villejuif, France) provided written informed consent for the collection of tissue samples for research, conformed to the principles set out in the WMA Declaration of Helsinki and the Department of Health and Human Services Belmont Report The transfer of clinical data from Gustave Roussy to Curie Institute was approved by Curie’s board: CRI Data [DATA230161]. Data from matched tumor samples from three patients at both baseline and after treatment were obtained, and the study was approved by the Gustave Roussy Scientific Committee (IRB No. 2023-249). Patient clinical data were described in Table EV1.

### Mice experiments

Six-week-old female athymic nude mice (Hsd: Athymic Nude-Foxn1nu, Mus Musculus, from Envigo) were subcutaneously inoculated with 2 million A375 cells. When tumors reached an average volume of 150 mm^3^, mice were fed with the control rodent diet or with a diet supplemented with 200 ppm PLX4720 (BRAF inhibitor, MedchemExpress) and 7 ppm PD0325901 (effective concentration of MEK inhibitor, MedchemExpress). eFT226 (1 mg/kg, MedchemExpress) or RBX0901 (1 mg/kg) were injected intraperitoneally two times per week for the entire duration of the experiment (70 days). EFT226 and RBX0901 were dissolved in DMSO to make a 0.1 mg/ml stock solution and then diluted in 20% 2-hydroxypropyl)-β-cyclodextrin (HPCD, #128446-35-5, BLDpharm) solution just before injection. Tumor growth was monitored three times/week in two dimensions using a digital caliper. Tumor volumes were calculated with the ellipsoid volume formula L × ω2 × 0.5, where L is the length and ω is the width. Animals were allocated to experimental groups so that the groups had similar mean tumor volumes before treatment initiation. The investigator was not blinded to the group allocation or when assessing the outcome. Animals were housed under pathogen-free conditions with food and water ad libitum. Cages were changed weekly, with food and water replenished as necessary. The animal holding room was maintained under controlled conditions: temperature 20–24 °C, humidity 45–65%, and a 12-h light/dark cycle.

Experiments were performed in accordance with the CCAC guidelines and approved by the ethical committee of the “Plateforme d’évaluation Préclinique” of Gustave Roussy (#33216202109241653613).

### Cell culture and generation of drug-tolerant cells

A375 melanoma cell lines were purchased from ATCC and were grown in high glucose (4.5 g/L) Dulbecco’s modified Eagle medium (Biowest, #L0101-500) supplemented with 10% FBS, 2 mM L-glutamine and 1% penicillin-streptomycin. M249 cell lines were obtained from Thomas Graeber’s laboratory at the Department of Molecular and Medical Pharmacology, University of California, Los Angeles, USA and were grown in Roswell Park Memorial Institute 1640 (Biowest, #L0500) medium supplemented with 10% FBS, 2 mM L-glutamine and 1% penicillin-streptomycin. All cells were grown in 5% CO_2_-humidified incubator at 37 °C and regularly tested for mycoplasma and authenticated by short tandem repeat (STR) profiling. Drug-tolerant cells were generated as previously described (Shen et al, 2019) through continuous treatment with BRAFi PLX4032 (500 nM) in combination with MEKi Cobimetinib (500 nM) for 72 h (A375) or PLX4032 (500 nM) in combination with 25 nM cobimetinib (M249). Approximately 70% of cells were killed and detached by the combination treatment. Surviving attached cells were trypsinized, washed once in PBS and replated in drug-free medium. The recovered surviving cells were maintained in drug-free conditions for a period of 9 days.

### Antibodies, siRNA, shRNA, RiboMap probes, and other reagents

The antibodies, compounds, small interfering RNAs (siRNAs), short hairpin RNA (shRNA) and antisense oligonucleotide sequences, as well as qPCR primers, used in this study are listed in Table EV2.

### Lentiviral infection

The pLKO.1 vector-based shRNA lentiviral constructs were purchased from Sigma TRC Mission shRNA library. HEK293T cells were transfected with 10 μg of the lentiviral construct, 5 μg of VSVG (Addgene #14888) and 10 μg of psPax2 (Addgene #12260) using Lipofectamine 2000 (Invitrogen™, #11668019). All the constructs are listed in Table EV2. After 48 h, the supernatant was collected, centrifuged to remove cell debris and filtered through a 0.45-μm filter, and polybrene was added to the medium before transduction to target cells. The same procedure was repeated on the second day. The cells were then selected with puromycin (2 μg ml^−1^). Knockdown efficiency was confirmed by qPCR.

### Quantitative proteomic analysis

#### Sample preparation

Untreated and drug-tolerant A375 cells were lysed in Urea buffer (8 M Urea, 50 mM Ammonium bicarbonate). Cell lysates were sonicated and then incubated at room temperature for 10 min. Afterward, the samples were centrifuged at 20,000×*g* for 10 min. Supernatants were collected, and protein extracts were quantified using the BCA Protein Assay Kit (Thermo FisherTM, #23225). 10 µg of total protein cell extract was reduced by incubation with 5 mM dithiothreitol (DTT) at 57 °C for 30 min and then alkylated with 10 mM iodoacetamide for 30 min at room temperature in the dark. Trypsin/LysC (Promega) was added at 1:100 (w/w) enzyme to substrate. Digestion was performed overnight at 37 °C. Samples were then loaded onto homemade C18 StageTips (AttractSPE Disk Bio C18-100.47.20, Affinisep) for desalting. Peptides were eluted using 40/60 CH3CN/H2O with 0.1% formic acid and vacuum concentrated to dryness. Peptides were reconstituted in 0.3% TFA before liquid chromatography–tandem mass spectrometry (LC–MS/MS) as described previously (Cañeque et al, 2025).

### LC–MS/MS analysis

Liquid chromatography (LC) was performed with a Vanquish Neo nanoLC system (Thermo Scientific) coupled to an Orbitrap Astral mass spectrometer (MS), interfaced by a Nanospray Flex ion source (Thermo Scientific). Peptides were injected onto a C18 column (inner diameter 75 µm × 50 cm double nanoViper PepMap Neo, 2 μm, 100 Å, Thermo Scientific) regulated at a temperature of 50 °C, and separated with a linear gradient from 100% buffer A (100% H2O in 0,1% formic acid) to 28% buffer B (100% CH3CN in 0,1% formic acid) at a flow rate of 300 nL/min over 104 min. The instrument was operated in data-independent acquisition (DIA) mode. MS full scans were recorded on the Orbitrap mass analyzer in centroid mode for ranges 380–980 m/z with a resolution of 240,000, a normalized automatic gain control (AGC) target set at 500% and a maximum injection time of 5 ms. The DIAs in the Astral analyzer were performed in centroid mode for a mass range of 380–980 m/z with a window width of 2 Da (without overlap), a maximum injection time of 3 ms and a normalized AGC target of 500% after fragmentation using higher-energy collisional dissociation (25% normalized collision energy).

### Data processing

For identification, the data were searched against the Homo sapiens (UP000005640) UniProt database using Spectronaut (v19.7; Biognosys) by directDIA+ analysis using default search settings. Enzyme specificity was set to trypsin, and a maximum of two missed cleavages was allowed. Carbamidomethyl was set as a fixed modification, and N-terminal acetylation and oxidation of methionine as variable modifications. The resulting files were further processed using myProMS (v3.10. https://github.com/bioinfo-pf-curie/myproms (Poullet et al, 2007))

For protein quantification, XICs from proteotypic peptides shared between compared conditions (TopN) with missed cleavages and carbamidomethylation were allowed. Median and scale normalization at the peptide level was applied on the total signal to correct the XICs for each biological replicate (*N* = 3). To evaluate the statistical significance of the change in protein abundance, a linear model (adjusted on peptides and biological replicates) was performed, and a two-sided *T*-test was applied on the fold change estimated by the model. The *p* values were then adjusted using the Benjamini–Hochberg FDR procedure. The list of proteins from the quantitative proteomic analyses are reported in Table EV3.

### GSEA analysis

Cluster profiler (Xu et al, 2024) and the fgsea library (Korotkevich et al, 2021) were used to perform gene set enrichment on the quantitative proteomic ratio values (Table EV3). Then, the gsea_go() function, using default parameters, was used to calculate the gene set enrichment. The enrichment plot was performed with the enrichplot package by using the enrichmentPlot function.

### Western blot

Cells were lysed in RIPA buffer (50 mM Tris-HCl, pH 7.4, 100 mM NaCl, 1% Nonidet P-40, 0.1% SDS, 0.5% Sodium deoxycholate with protease inhibitor). The protein quantification was measured using the BCA Protein Assay Kit (Thermo Fisher^TM^, #23225). For immunodetection, membranes were blocked in a buffer containing 0.1% Tween-20 (TBST) and supplemented with 5% powdered milk and exposed to appropriate antibodies.

### Quantitative real-time PCR analysis

Total RNA was extracted using TRIzol-chloroform and treated with DNase I (TURBO DNA-free; Invitrogen™, #AM1907) according to the manufacturer’s instructions. cDNA was synthesized using SuperScript III reverse transcriptase (Invitrogen^TM^, #18080044). Quantitative real-time PCR was performed using Power SYBR Green PCR Master Mix (Thermo Scientific™, #4367559). For normalization, we applied the geometric mean of multiple housekeeping genes (TBP, 18S, Actin).

### Colony assay

Drug-tolerant cells were seeded in six-well plates and treated with the indicated drug combinations for 3 weeks. Cells were washed once with PBS, stained with a 20% ethanol solution containing 0.5% crystal violet (Sigma-Aldrich, #C0775) for 10 min, washed with PBS and destained in tap water. Images were analysed by the ColonyArea plugin on Fiji software (Guzmán et al, 2014).

### CRISPR/Cas9 5’UTR deletion

CRISPR/Cas9 gene editing was performed using 2 fluorescent plasmid-based vectors (Vector builder) co-expressing hCas9 nuclease and sequence-specific sgRNA targeting *53BP1* 5’UTR (Table EV2). EGFP and mCherry were used as fluorescent markers for transfected cells.

A375 cells were transfected with 4 µg of each plasmid using Lipofectamine 2000 (Invitrogen™, #11668019) for 72 h. Cells co-expressing GFP and mCherry were sorted by FACS and seeded into 96-well plates at one cell per well. Single-cell-derived clones were expanded, and gene editing efficiency was assessed by Sanger sequencing.

### Random plasmid integration assay

Random plasmid integration assay was performed as previously described (Rother et al, 2020) with some modifications. Briefly, untreated and drug-tolerant cells were seeded in six-well plates, and the following day were treated with 30 nM eFT226 or DMSO for 24 h. At day 3, cells were transfected with 2–2.5 μg gel-purified BamHI-EcoRI-linearized pEGFP-C1 plasmid for 48 h. When siRNA treatment was included in the experiment, two rounds of siRNA transfection (30 nM) with Lipofectamine RNAiMAX (Invitrogen^TM^, #13778150) were performed on days 2 and 3, followed by transfection with the pEGFP-C1 plasmid. In experiments performed in A375 WT and Δ 5’UTR clones, untreated and drug-tolerant A375 cells from each cell line were seeded in six-well plates, and the following day were transfected with the pEGFP-C1 plasmid for 48 h.

On day 5, cells were collected, counted, seeded, and grown in medium lacking or containing 0.5 mg/mL G418. Transfection efficiency of the linearized pEGFP-C1 was determined by flow cytometry. The cells were incubated at 37 °C to allow colony formation for 2 weeks by refreshing media every 3-4 days. The cells were then stained with a 20% ethanol solution containing 0.5% crystal violet (Sigma-Aldrich, #C0775) and counted using ImageJ. Random plasmid integration events (number of G418-resistant colonies) were normalized by the plating efficiency (number of colonies without G418) and by transfection efficiency. The pEGFP-C1 plasmid was a kind gift from van Attikum’s lab (Leiden University Medical Center, Leiden, The Netherlands).

### HPRT mutagenesis assay

The HPRT assay was performed as previously described (Glaab et al, 1998; Miles and Hawkins, 2018) with some modifications. Briefly, untreated and drug-tolerant A375 cells were cultured in drug-free media for 6 days to ensure the removal of any remaining endogenous HPRT activity. When applicable, cells were then treated with 30 nM eFT226 or DMSO for 24 h and then plated onto a 150 mm dish and treated with 20 µM 6-TG. Media with fresh 6-TG was changed every 3–4 days for 15-20 days until visible colonies appeared. Cells were also seeded at low density in drug-free media to determine plating efficiency. The cells were then stained with a 20% ethanol solution containing 0.5% crystal violet (Sigma-Aldrich, #C0775), and colonies were counted using ImageJ.

The HPRT mutation frequency was calculated as the ratio of the number of HPRT−mutant colonies in 6-TG media to the number of surviving colonies plated in complete media to determine clonal efficiency.

### AHA-mediated RIBOsome isolation

mRNA enrichment in translating ribosomes was analyzed using the AHARIBO RNA System kit (#AHA-RM12, Immagina Biotechnology). The assay was performed according to the manufacturer’s protocol.

The extracted RNA was quantified using Qubit (Thermo Scientific), and 200–500 ng was used for subsequent RT-qPCR experiments.

### RiboMap assay

RiboMap assay was performed as previously described (Zeng et al, 2023). The Splints and *IQGAP1*-specific Padlocks and Primers probes were designed by the previous study (Zeng et al, 2023). The *53BP1*-specific hybridization regions in Padlocks and Primers probes were designed using Picky 2.2 software. To visualize translating mRNAs, fluorescent probes complementary to the DNA amplicons were used instead of in situ sequencing. Probes were purchased from IDT, and probe sequences are listed in Table EV2. Harringtonine treatment was performed by adding the drug in the medium to a final concentration of 5 µM and incubating at 37 °C for 5 min.

Images were acquired using a Leica SP8X inverted confocal laser scanning microscope (CLSM) equipped with a 63X oil immersion objective (NA = 1.4). Sequential excitation was performed using a laser diode at 405 nm and a white light laser (WLL) at 488, 594, and 650 nm. Emission was detected using GaAsP hybrid photon detectors and a photomultiplier tube (PMT), with detection windows set to 415–465 nm for 405 nm excitation, 500–550 nm for 488 nm excitation, 605–670 nm for 594 nm excitation, and 660–710 nm for 650 nm excitation. During acquisition, an additional channel was added to highlight cells and facilitate their detection for quantification. The BrightR mode on the Hybrid Detector was used, with the detection window set to 575–660 nm for excitation at 488 nm. Image acquisition and system control were performed using LAS X software (Leica Microsystems). At least 30 cells per sample were imaged, and the images were processed with Fiji (Schindelin et al, 2012) by using the MIC-MAQ plugin (https://github.com/MultimodalImagingCenter/MIC-MAQ). 3D Z-stack images are converted to 2D images by applying a Z maximum intensity projection. Nuclei and cells are segmented from the DAPI and the additional fluorescence images using the Cellpose deep-learning network (Stringer et al, 2021) with the Cyto3 model. For cell, images are first We created artificially an other channel were autofluorescence of the cytoplasm was used to highlight cells contour. Segmentation is performed using a diameter corresponding to the expected size of nuclei or cells, typically set to 100 pixels for nuclei and 300 pixels for cells. To facilitate spot detection, the background was removed using the subtract background function with a rolling ball radius of 30 pixels. During analysis with MIC-MAQ, nuclei and cells are exported as individual regions in ZIP files, with one ZIP file per image in the project. Subsequently, morphological and intensity parameters, including area, mean intensity, and spot counting using the Find Maxima function, are measured for each cell in Alexa Fluor 488, 594, and 647 channels.

### Immunofluorescence and multiplex immunostaining

For immunostaining, untreated and drug-tolerant cells were plated in six-well plates with coverslips and fixed with 4% PFA on day 1 after drug removal. When applicable, 24 h treatment with eFT226 (30 nM) or DMSO was performed before fixation. Cells were permeabilized in 0.5% PBS-Triton, washed with PBS, and then blocked for 1 h at room temperature in PBS containing 0.1% Tween® 20 Detergent (Sigma-Aldrich #P6585) and 5% bovine serum albumin (Sigma-Aldrich #A9576). Primary and secondary antibodies were diluted in blocking solution. Primary antibodies were incubated for 1 to 3 h at room temperature, depending on the antibody, and secondary antibodies were incubated for 1 h at room temperature. Cells were then washed using PBS with 0.1% Tween® 20 (Sigma-Aldrich #P6585), and coverslips were mounted on glass slides using ProLong™ Diamond with DAPI (Invitrogen™ #P36971) mounting medium. Fluorescent images were acquired on a Leica widefield DM6000B upright microscope equipped with a 63x oil objective (N.A = 1.4) and coupled with a Hamamatsu sCMOS ORCA Flash4.0 camera (pixel size: 6.5 µm). Alexa Fluor 488 DAPI were detected using bloc filters (DAPI: ex BP405/60 - em BP 470/50; FITC: ex BP470/40 - em BP525/50). The system is driven by MetaMorph® (Molecular Devices) software. Images were analyzed using a semi-automatic macro on Fiji software. Nuclei containing ≥10 distinct foci were defined as foci-positive, and the percentage of positive nuclei was calculated as [(number of foci-positive nuclei)/(number of nuclei scored)]*100. A minimum of 100 nuclei per sample were scored, and data were collected from three biological replicates.

For multiplex immunostaining, untreated and drug-tolerant cells at day 1 of drug removal were collected, pelleted, and embedded in OCT compound. The samples were then flash-frozen in liquid nitrogen. Frozen blocks were sectioned using a cryostat, and tissue sections were mounted onto poly-L-lysine–coated coverslips. Akoya Biosciences CODEX multiplex immunostaining was performed according to the manufacturer’s instructions.

In brief, coverslips were fixed with acetone, rehydrated and then stained with a combination of DNA-barcoded primary antibodies, including in-house conjugated antibodies, washed and post-fixed in ice-cold methanol. They were mounted on a CODEX system for multiple cycle immunostaining and imaged using a Keyence microscope with CODEX instrument manager and Keyence software (BZ-X800 viewer). Secondary antibodies were fed to the instrument in a pre-prepared 96-well plate. In total, five cycles of immunostaining (including two blanks with only nuclear staining) were run, consisting of DAPI nuclear staining, Atto550-, Cy5-, and Alexa Fluor 488 fluorophores. CODEX processor (v. 1.7.0.6 and 1.8.3.14) performed automated image registration, extended depth of focus, shading correction, and autofluorescence and background subtraction.

### Multiplex immunostaining data analysis

Images were automatically analyzed using QuPath software (Bankhead et al, 2017). All images (in qptiff format) were imported into a QuPath project, and nuclei were automatically segmented on DAPI-stained images using the Cellpose deep-learning algorithm (Stringer et al, 2021). The model employed was Cyto2 with a cell diameter set to 15 pixels and a fixed pixel size of 0.5.

Cell boundaries were estimated by expanding the detected nuclei by a maximum of 15 pixels and limiting cell size relative to the nucleus with a factor of 5. Mean intensity values of various markers were calculated for each cell and exported as comma-separated values (CSV) files for further analysis.

To visualize the data, heat maps were generated using GraphPad Prism. Each row in the data matrix represents a single cell, with color-coding reflecting the relative intensity of the corresponding marker signal. In the heat maps, cells were organized in ascending order based on 53BP1 expression levels.

### Immunohistochemistry (IHC)

IHC was performed using the BenchMark Ultra automated staining system (Roche Diagnostics). Antigen retrieval was carried out with the CC1 buffer (pH 8.0) for 64 min at 95 °C. Tissue sections were then incubated with the anti-53BP1 antibody (Cell Signaling Technology, #88439) at a 1:400 dilution for 1 h at room temperature. Detection was performed using the UltraView Universal DAB Detection Kit (Roche) according to the manufacturer’s instructions. Quantification of 53BP1 staining was performed by a pathologist (J-Y.S.) using an H-score–based approach. Cells were classified according to staining intensity (1 = weak, 2 = moderate, 3 = strong, 4 = very strong), and the number of cells at each intensity level was recorded. A composite score was calculated as the sum of the number of cells at each intensity multiplied by the corresponding intensity, normalized to the analyzed tissue area (µm²).

### In vitro transcription

T7-LucF sequences were amplified by PCR using the pCREL plasmid as a template (Cammas et al, 2016; Cammas et al, 2007). The primers used are listed in Table EV2. (AG)_10_-, (UC)_10_-, (AC)_10_-, and (UC)_10_ reporter transcripts were synthesized using the *mMESSAGE mMACHINE™* T7 Transcription Kit (Invitrogen™, Cat. #AM1344) according to the manufacturer’s instructions. 0.2 µg of the T7-LucF construct was used as the transcription template. The in vitro transcription reaction was carried out at 37 °C for 2 h. Following transcription, template DNA was removed by digestion with TURBO DNase (Invitrogen™, #AM2238), and RNA was purified using phenol–chloroform.

### Chemical synthesis of compounds

The synthesis of most of the compounds has been disclosed in the following patent: F. Marion, EB Kaloun, F Lieby-Muller, M. Perez, JP Annereau, L. Créancier.

**Scheme 1:**
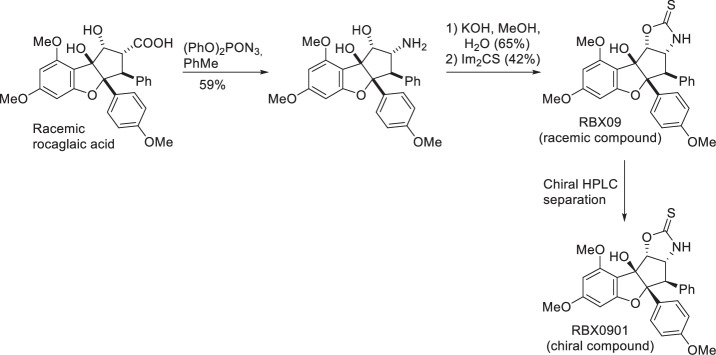
Synthesis of RBX09 and RBX091.

FR3023290 A1 2016-01-08. As a representative example, racemic RBX09 was prepared from rocaglaic acid by a Curtius reaction, followed by a saponification and a ring closure with thiocarbonyldimiimidazole (see Scheme 1). Chiral separation by HPLC afforded enantiomerically pure RBX091.

**Scheme 2:**
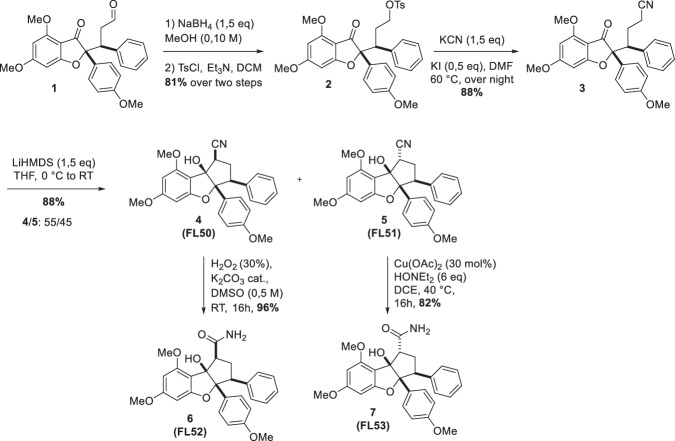
Synthesis of novel flavagline derivatives.

The synthesis of original compounds was performed as described in Scheme 2. Nitrile **3** was prepared from known aldehyde **1** by reduction, tosylation and SN2 substitution with potassium cyanide. Treatment of intermediate **3** with LiHMDS in THF led to flavaglines **4** and **5** (Fl 50 and FL51) in high yield (88%) and in a 55:45 ratio. In the next step, *cis*-nitrile **4** was converted to the desired *cis*-amide **6** (FL52) by using H_2_O_2_ with 96% yield. To hydrolyze *trans*-cyano **5**, we used *N*,*N*-diethylhydroxylamine and Cu(OAc)_2_ as a catalyst to afford the desired *trans*-amide **7** (FL53) in 82% yield.

#### (S)-3-((R)-4,6-dimethoxy-2-(4-methoxyphenyl)-3-oxo-2,3-dihydrobenzofuran-2-yl)-3-phenylpropyl 4-tosylate (2)

To a solution of aldehyde **1** (480 mg, 1.11 mmol, 1 eq) in MeOH (11 mL) under argon, NaBH_4_ (63 mg, 1.66 mmol, 1.5 eq) was added in one portion at 0 °C. The reaction was stirred, at the same temperature, for 30 min then concentrated under vacuum. The crude was dissolved with EtOAc, and the organic layer was washed with water, then with brine, dried over Na_2_SO_4_ and concentrated to give the primary alcohol as white foam. The obtained alcohol was then dissolved in DCM (6 mL), then Et_3_N (0.18 mL, 1.33 mmol, 1.2 eq), TsCl (212 mg, 1.11 mmol, 1 eq), and DMAP (13 mg, 0.11 mmol, 10 mol%) were added successively under argon. The mixture was stirred at room temperature overnight, then treated with HCl (1 M). The aqueous layer was extracted with DCM, then the organic layer was washed with brine, dried over Na_2_SO_4_, concentrated, and purified by column chromatography using pentane/EtOAc (6/4) as eluent to give tosylate **2** (529 mg, 81%) as a white foam. ^1^H NMR (500 MHz, CDCl_3_) δ 7.60 − 7.53 (4H, m), 7.21 (2H, d, *J* = 8.1 Hz), 7.13-7.11 (2H, m), 7.05-7.03 (3H, m), 6.88 (2H, d, *J* = 8.9 Hz), 6.19 (1H, d, *J* = 1.4 Hz), 5.77 (1H, d, *J* = 1.4 Hz), 3.86-3.80 (4H, m), 3.79 (3H, s), 3.60 (1H, dd, *J* = 12.2, 3.1 Hz), 3.53 (1H, m), 2.41 (3H, s), 2.14 (1H, m), 1.95 (1H, m); ^13^C{1H} NMR (125 MHz, CDCl_3_) δ 195.4, 174.4, 169.7159.5, 159.1, 144.6, 136.0, 132.8, 129.8, 129.7, 128.7, 128.3, 127.8, 127.2, 126.3, 114.1, 103.9, 94.1, 92.8, 88.4, 68.4, 56.0, 55.9, 55.4, 48.5, 28.9, 21.7; HRMS (ESI-TOF) m/z [M + H]^+^ calcd for C_33_H_33_O_8_S^+^ 589.1891, found 589.1903.

#### (S)-4-((R)-4,6-dimethoxy-2-(4-methoxyphenyl)-3-oxo-2,3-dihydrobenzofuran-2-yl)-4-phenylbutanenitrile (3)

To a solution of **2** (450 mg, 0.76 mmol, 1 eq) in DMF (2.1 mL) under argon, KCN (75 mg, 1.15 mmol, 1.5 eq) and KI (63 mg, 0.38 mmol, 0.5 eq) were added. The mixture was stirred at 60 °C overnight. After cooling to room temperature, the mixture was diluted with water and extracted three times with EtOAc. The combined organic layers were washed with brine, dried over Na_2_SO_4_, concentrated, and purified by column chromatography using pentane/EtOAc (6/4) as eluent to give nitrile **3** (300 mg, 88%) as a white foam. ^1^H NMR (500 MHz, CDCl_3_) δ 7.63 (2H, d, *J* = 8.5 Hz), 7.29 (2H, d, *J* = 7.3 Hz), 7.19-7.09 (3H, m), 6.90 (2H, d, *J* = 8.7 Hz), 6.22 (1H, d, *J* = 0.9 Hz), 5.79 (1H, d, *J* = 0.9 Hz), 3.84 (3H, s), 3.79 (3H, s), 3.67 (3H, s), 3.62 (1H, dd, *J* = 12.7, 2.6 Hz), 2.19 (1H, m), 2.07 (1H, m), 1.99-1.85 (2H, m); ^13^C{1H} NMR (125 MHz, CDCl_3_) δ 195.16, 174.4, 169.7, 159.6, 159.2, 135.6, 129.6, 128.6, 127.7, 126.2, 119.2, 114.2, 103.8, 93.8, 92.9, 88.5, 56.0, 55.9, 55.4, 51.5, 25.6, 15.4; HRMS (ESI-TOF) m/z [M + H]^+^ calcd for C_27_H_26_NO_5_^+^ 444.1805, found 444.1815.

#### (1R,3S,3aR,8bR)-8b-hydroxy-6,8-dimethoxy-3a-(4-methoxyphenyl)-3-phenyl-2,3,3a,8b-tetrahydro-1H-cyclopenta[b] benzofuran-1-carbonitrile (4, FL50) and (1S,3S,3aR,8bR)-8b-hydroxy-6,8-dimethoxy-3a-(4-methoxyphenyl)-3-phenyl-2,3,3a,8b-tetrahydro-1H-cyclopenta[b]benzofuran-1-carbonitrile (5, FL51)

To a solution of **3** (260 mg, 0.59 mmol, 1 eq) in anhydrous THF (5.7 mL) under argon at 0 °C, a solution 1 M of LiHMDS in THF (0.88 mL, 0.88 mmol, 1.5 eq) was added dropwise. The mixture was stirred at room temperature for 2 h. The mixture was diluted with water and extracted with EtOAc. The organic layer was washed with brine, dried over Na_2_SO_4_, concentrated, and purified by column chromatography using pentane/EtOAc (7/3) as eluent to give **1** (FL50, 126 mg, 48%) and **5** (FL51, 102 mg, 40%) as white foams.

##### *Cis* isomer 4 (FL50, less polar)

^1^H NMR (500 MHz, CDCl_3_) δ 7.17 (2H, d, *J* = 8.9 Hz), 7.14-7.07 (3H, m), 6.97-6.91 (2H, m), 6.70 (2H, d, *J* = 8.9 Hz), 6.22 (1H, d, *J* = 1.7 Hz), 6.10 (1H, d, *J* = 1.7 Hz), 3.90 (3H, s), 3.83 (3H, s), 3.78 (1H, dd, J = 11.6, 7.9 Hz), 3.71 (3H, s), 3.58 (1H, dd, *J* = 14.5, 6.0 Hz), 2.81 (1H, m), 2.59 (1H, m), 2.39 (1H, brs); ^13^C{1H} NMR (125 MHz, CDCl_3_) δ 164.3, 159.4, 158.9, 157.4, 137.0, 128.9, 128.0, 128.0, 127.0, 126.1, 119.1, 113.0, 110.3, 102.4, 92.4, 89.0, 87.1, 55.8, 55.8, 55.2, 54.3, 39.0, 33.1; HRMS (ESI-TOF) m/z [M + H]^+^ calcd for C_27_H_26_NO_5_^+^ 444.1805, found 444.1809.

##### *Trans* isomer 5 (FL51, more polar)

^1^H NMR (500 MHz, CDCl_3_) δ 7.18-7.05 (7H, m), 6.65 (2H, d, *J* = 8.9 Hz), 6.22 (1H, d, *J* = 1.8 Hz), 6.13 (1H, d, *J* = 1.8 Hz), 4.04 (1H, dd, *J* = 13.3, 6.0 Hz), 3.89 (3H, s), 3.83 (3H, s),3.82 (1H, m), 3.68 (3H, s), 2.89 (1H, m), 2.59 (1H, ddd, *J* = 13.0, 6.0, 2.1 Hz), 2.19 (1H, s); ^13^C{1H} NMR (125 MHz, CDCl_3_) δ 164.5, 160.1, 158.9, 158.0, 137.7,128.9, 128.1, 126.8, 120.4, 127.0, 126.1, 119.1, 113.0, 107.5, 102.8, 92.8, 92.1, 88.7, 55.7, 55.5, 55.2, 42.3, 33.6; HRMS (ESI-TOF) m/z [M + H]^+^ calcd for C_27_H_26_NO_5_^+^ 444.1805, found 444.1816.

#### (1S,3S,3aR,8bR)-8b-hydroxy-6,8-dimethoxy-3a-(4-methoxyphenyl)-3-phenyl-2,3,3a,8b-tetrahydro-1H-cyclopenta[b] benzofuran-1-carboxamide (6, FL52)

To a solution of nitrile **4** (50 mg, 0.11 mmol, 1 eq) in DMSO (0.3 mL), H_2_O_2_ (30%, 32 µL) and K_2_CO_3_ (3 mg, 20 mol%) were added at room temperature. The mixture was stirred at the same temperature overnight. The mixture was diluted with water and extracted with EtOAc. The organic layer was washed with brine, dried over Na_2_SO_4_, concentrated, and purified by column chromatography using DCM/MeOH (95/5) as eluent to give amide **6** (FL52) (50 mg, 96%) as a white solid. ^1^H NMR (500 MHz, CDCl_3_) δ 7.31 (2H, d, *J* = 8.1 Hz), 7.12-7.04 (3H, m), 7.03-6.97 (2H, m), 6.75 (1H, brs), 6.71 (2H, d, *J* = 8.6 Hz), 6.27 (1H, d, *J* = 1.4 Hz), 6.14 (1H, d, *J* = 1.4 Hz), 5.83 (1H, brs), 3.93 (3H, s), 3.82 (3H, s), 3.72-3.66 (4H, m), 3.51 (1H, dd, *J* = 14.9, 6.1 Hz), 3.14 (1H, m), 2.29 (1H, brs), 2.13 (1H, m); ^13^C{1H} NMR (125 MHz, CDCl_3_) δ 173.7, 163.6, 159.4, 158.7, 156.7, 138.3, 128.9, 128.2, 127.7, 126.7, 126.5, 113.0, 112.5, 103.2, 92.6, 89.6, 87.6, 55.8, 55.2, 53.0, 50.9, 30.1; HRMS (ESI-TOF) m/z [M + Na]^+^ calcd for C_27_H_27_NNaO_6_ 484.1731, found 484.1721.

#### (1R,3S,3aR,8bR)-8b-hydroxy-6,8-dimethoxy-3a-(4-methoxyphenyl)-3-phenyl-2,3,3a,8b-tetrahydro-1H-cyclopenta[b] benzofuran-1-carboxamide (7, FL53)

To a solution of nitrile **5** (60 mg, 0.15 mmol, 1 eq) in DCE (0.3 mL), Cu(OAc)_2_ (8 mg, 0.04 µmol, 30 mol%) and HONEt_2_ (78 mg, 0.88 mmol, 6 eq) were added at room temperature. The mixture was stirred at 40 °C overnight. After cooling to room temperature, the mixture was diluted with water and extracted with EtOAc. The organic layer was washed with brine, dried over Na_2_SO_4_, concentrated, and purified by column chromatography using DCM/MeOH (97/3) as eluent to give amide **7** (FL53) (55 mg, 82%) as a white solid.

### In vitro translation

In vitro translation assays were performed with Rabbit Reticulocyte Lysate System (Promega Cat. No. L4960), adding 0.5 ng of reporter mRNAs according to the manufacturer’s instructions. The drugs at the indicated concentrations were added to the reaction for 1 h 30 min at 30 °C prior to the measurement of luciferase activity using Dual-Luciferase Reporter Assay System (Promega, #E1910).

### Luciferase assay

Untreated, drug-tolerant cells and cells released from the drugs for 9 days were transiently transfected with psiCHECK ™-2 Vector (Promega, #C8021) using Lipofectamine LTX Reagent (Invitrogen™, #15338100). In psiCHECK ™-2 Vector, 317 bp downstream of the SV40 early enhancer/promoter transcription start site, corresponding to nt 372 to 684, were removed. 53BP1, Tubulin, c-Myc and Cyclin D1 5’UTR sequences were cloned at the level of the NheI site, upstream to the Renilla coding sequence. The *Firefly* luciferase signal served as an internal transfection normalization control. After 48 h, part of the transfected cells was harvested for RNA extraction to measure RNA expression by RT-qPCR, while the rest of the cells were used to measure the luciferase activity using the Dual-Luciferase Reporter Assay System (Promega, #E1910). Data were presented as the ratio between Renilla and Firefly luciferase RNA expression or luminescence activities, respectively.

For experiments performed in the presence of drugs, treatments at the indicated concentrations were performed 24 h after psiCHECK ™-2 vector transfection, for 12 (53BP1 5’UTR reporter) or 24 h (Tubulin, c-Myc and Cyclin D1 reporters). At the end of drug treatment, luciferase activity was analyzed.

### Proliferation assay

A375 cells were seeded in triplicate in 96-well plates. The day after, cells were treated with the indicated concentrations of drugs. Live-cell proliferation was monitored using the IncuCyte S3 System (Sartorius). Images of each well were captured every 3 h over a 72-h period. Proliferation data were quantified based on confluency measurements, and values were normalized to the confluency at time zero (T0) to account for initial seeding variability.

For long-term proliferation assay with Δ5’UTR clones, drug-tolerant cells were seeded into T25 flasks and treated the following day with 100 nM BRAFi and 10 nM MEKi. Images of each flask were acquired weekly for 8 weeks under continuous BRAFi/MEKi treatment using the IncuCyte® S3 Live-Cell Analysis System (Sartorius). Proliferation was quantified as percent confluence (plate coverage), and values were normalized to the confluence at time zero (T0) to account for variability in initial cell seeding. Confluence measurements were obtained using the image analysis module in the IncuCyte system.

### Quantification and statistical analysis

For cell-based assays, all experiments were performed in at least three independent biological replicates, unless otherwise reported in the figure legend. Statistical analyses were performed using Prism 10.3 (GraphPad). Statistical significance, statistical tests, and sample sizes were determined as indicated in the figure legends. Significant differences between experimental groups were stated as: *p < 0.05, **p < 0.01, ***p < 0.001, or ****p < 0.0001. The data were shown as mean ± SEM. All experiments were conducted non-blinded, and we included all samples in our analyses. No samples were excluded from the analyses unless a technical failure occurred. For in vivo xenograft studies, mice bearing tumors of ~150 mm³ were randomly assigned to treatment groups. Animals were excluded only if tumor engraftment failed prior to randomization. Investigators were not blinded to group allocation during treatment administration or data acquisition.

### Figure preparation

Schematics in Fig. 2A,C were created with BioRender.com under a BioRender publication license.

## Supplementary information

Table EV1

Table EV2

Table EV3

Peer Review File

Source data Fig. 1

Source data Fig. 2

Source data Fig. 3

Source data Fig. 4

Source data Fig. 5

Source data Fig. 6

Source data Fig. 7

Figure EV1 Source Data

Figure EV2 Source Data

Figure EV3 Source Data

Figure EV4 Source Data

Figure EV5 Source Data

Figure EV6 Source Data

Figure EV7 Source Data

Figure EV8 Source Data

Figure EV9 Source Data

Expanded View Figures

## Data Availability

All data were available in the main text or the supplementary materials. The mass spectrometry proteomics data have been deposited to the ProteomeXchange Consortium via the PRIDE partner repository (https://www.ebi.ac.uk/pride/archive/projects/PXD068007) with the dataset identifier PXD068007. The source data of this paper are collected in the following database record: biostudies:S-SCDT-10_1038-S44321-026-00479-5.
