# Supplementary material for: Selective mRNA translation determines adaptative mutability of melanoma cells to anti-BRAF/MEK combination therapy
Source: EMBO Mol Med. 2026 Jul 20;18(8):3278–317. doi: 10.1038/s44321-026-00479-5 (PMC13470486; doi:10.1038/s44321-026-00479-5)
Supplement: Supplementary file 1 [file 44321_2026_479_MOESM21_ESM.pdf]

Expanded View Figures

Figure EV1. Monitoring DNA repair upon targeted therapy.

(**A**) GSEA enrichment of the differentially expressed proreins. NES = −2.321, q value = 2.81e^{-05}. (**B**) Western blot illustrating 53BP1, XRCC4, KU70, RAD51, and LIG3 protein levels in A375 cells after 3 days of treatment with DMSO (DMSO) or BRAFi/MEKi treatment (BRAFi/MEKi). GAPDH serves as a loading control, and the relative quantification from three independent experiments is indicated. p values were calculated by unpaired, two-tailed Student's t-test (53BP1: *$p = 0.0373$, XRCC4: $p = 0.3820$, KU70: $p = 0.3948$, RAD51: *$p = 0.0133$, LIG3: **$p = 0.0012$). (**C**) RT-qPCR quantification of *53BP1* mRNA level in control cells (DMSO) or in BRAFi/MEKi A375 and M249 drug-tolerant cells. Data represent the mean ± SEM of six independent experiments. Data were normalized using the geometric mean of TBP, Actin and 18S housekeeping genes (A375: $p = 0.1445$, M249: $p = 0.9540$). (**D**) Representative images of immunofluorescence in M249 control (DMSO), drug-tolerant cells (BRAFi/MEKi) and drug-tolerant cells released from targeted therapy for 9 days (BRAFi/MEKi rel) stained for 53BP1 (green) and nuclei (DAPI, blue). (bottom) Representative images of immunofluorescence in cells stained for RAD51 (magenta) and nuclei (DAPI, blue). Scale bar, 10 µm. The quantification of the signal analyzed by percentage of cells with ≥10 foci/nucleus is reported, and data represent the mean ± SEM from four independent experiments. p values were calculated by paired, two-tailed Student's t-test (53BP1: DMSO vs BRAFi/MEKi **$p = 0.0077$, BRAFi/MEKi vs BRAFi/MEKi rel *$p = 0.0430$, RAD51: DMSO vs BRAFi/MEKi **$p = 0.0012$, BRAFi/MEKi vs BRAFi/MEKi rel $p = 0.4765$). Source data are available online for this figure.

© The Author(s)

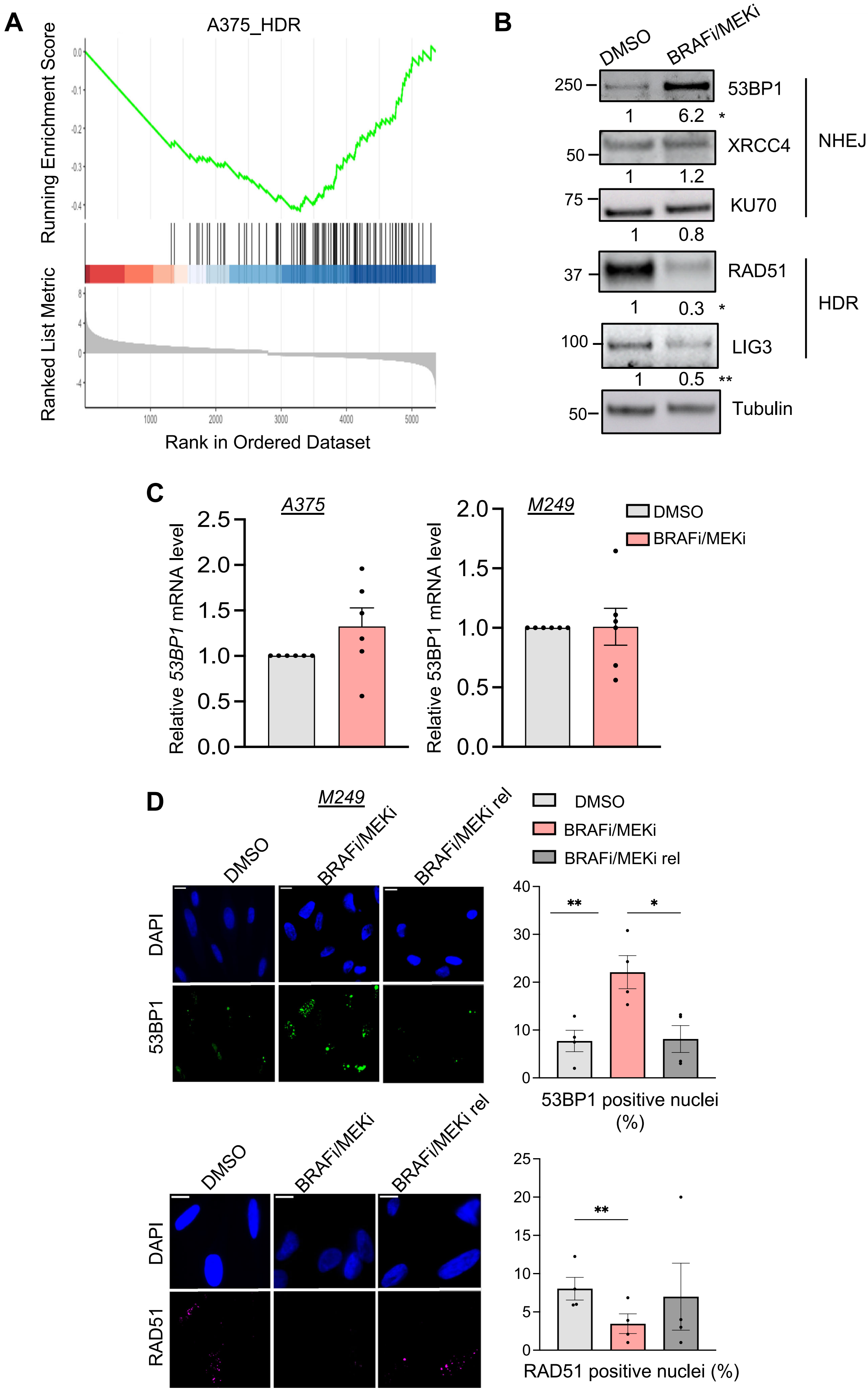
A
A375_HDR
Running Enrichment Score
Ranked List Metric
Rank in Ordered Dataset
B
DMSO
BRAFi/MEKi
250
53BP1
1
6.2
*
50
XRCC4
1
1.2
75
KU70
1
0.8
NHEJ
37
RAD51
1
0.3
*
100
LIG3
1
0.5
**
HDR
50
Tubulin
C
A375
M249
Relative 53BP1 mRNA level
DMSO
BRAFi/MEKi
D
M249
DMSO
BRAFi/MEKi
BRAFi/MEKi rel
DAPI
53BP1
53BP1 positive nuclei (%)
RAD51
RAD51 positive nuclei (%)

 © The Author(s)

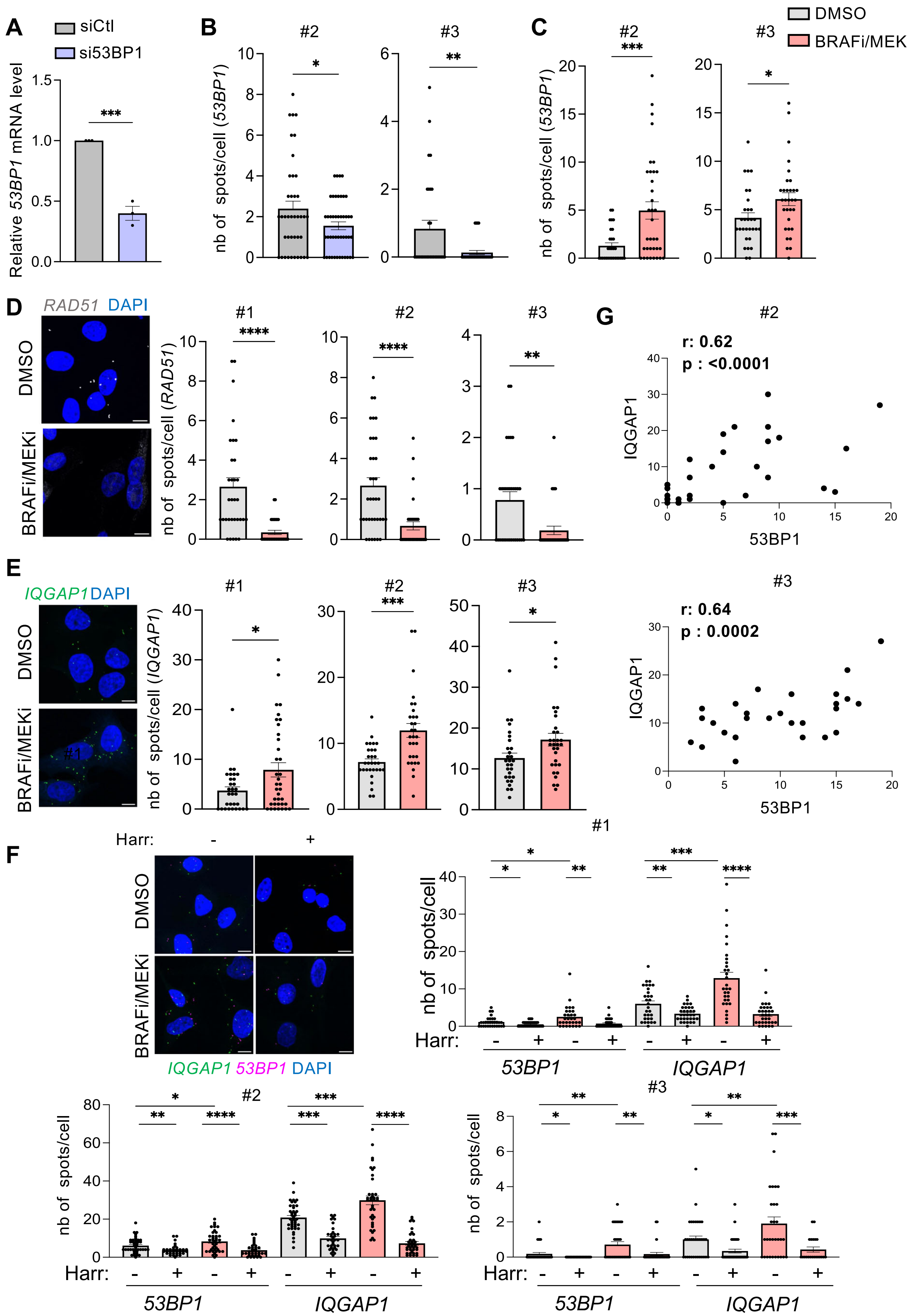
A
siCtl
si53BP1
Relative 53BP1 mRNA level
B
#2
#3
nb of spots/cell (53BP1)
C
DMSO
BRAFi/MEK
D
RAD51 DAPI
DMSO
BRAFi/MEKi
#1
nb of spots/cell (RAD51)
G
r: 0.62
p : <0.0001
IQGAP1
53BP1
E
IQGAP1 DAPI
nb of spots/cell (IQGAP1)
r: 0.64
p : 0.0002
F
Harr:
IQGAP1 53BP1 DAPI
nb of spots/cell

Figure EV2. Single-cell analysis of translating mRNAs.

(**A**) RT-qPCR quantification of *53BP1* mRNA level in A375 cells transfected with control siRNA (siCtl, 25 nM) or with siRNA targeting *53BP1* (si53BP1, 25 nM) corresponding to Fig. 2D,E. Data represent the mean ± SEM from three independent experiments (unpaired Student's *t*-test, ***$p = 0.0005$). (**B**) Quantification of translating *53BP1* mRNAs (spots) in A375 cells transfected with siCtl or si53BP1. Data shown represent the mean ± SEM of the number of spots/cells quantified in the other two independent experiments (corresponding to Fig. 2E) (unpaired Student's *t*-test,*$p = 0.036$, **$p = 0.0097$). (**C**) Quantification of translating *53BP1* mRNAs (spots) in A375 control cells (DMSO) or in cells surviving targeted therapy (BRAFi/MEKi). Data shown represent the mean ± SEM of the number of spots/cells quantified in other two independent experiments (corresponsing to Fig. 2G) (unpaired Student's *t*-test, *$p = 0.0265$, ***$p = 0.0006$). (**D**) (left): Representative confocal images of translating *RAD51* mRNAs (gray) assessed by RiboMap assay in A375 control cells (DMSO) or in cells surviving targeted therapy (BRAFi/MEKi). Nuclei are stained with DAPI (blue). Scale bar: 10 µm. (right): Quantification of translating *RAD51* mRNAs (spots). Data represent the mean ± SEM of the number of spots/cells quantified in three independent experiments (unpaired Student's *t*-test, #1, #2 ****$p \leq 0.0001$, #3: **$p = 0.0017$). (**E**) (left): Representative confocal images of translating *IQGAP1* mRNAs (green) assessed by RiboMap assay in A375 control cells (DMSO) or in cells surviving targeted therapy (BRAFi/MEKi). Nuclei are stained with DAPI (blue). Scale bar: 10 µm. (right): Quantification of translating *IQGAP1* mRNAs (spots). Data represent the mean ± SEM of the number of spots/cells quantified in three independent experiments (unpaired Student's *t*-test, #1: *$p = 0.0175$, #2: ***$p = 0.0001$, #3: *$p = 0.0285$). (**F**) (top): Representative confocal images of translating *53BP1* (magenta) and *IQGAP1* (green) mRNAs in DMSO and BRAFi/MEKi-treated cells in the presence or absence of Harringtonine (Harr) treatment (5 µM). Nuclei are stained with DAPI (blue). Scale bar: 10 µm. (bottom): Quantification of translating *53BP1* and *IQGAP1* mRNAs (spots). Data represent the mean ± SEM of the number of spots/cells quantified in three independent experiments (unpaired Student's *t*-test, *$p \leq 0.05$, **$p \leq 0.01$,***$p \leq 0.001$, ****$p \leq 0.0001$). (**G**) Scatterplot illustrating the correlation between translating *53BP1* mRNAs and *IQGAP1* mRNAs found in the other two biological replicates in A375 drug-tolerant cells (corresponding to Fig. 2I). Pearson correlation coefficient (*r*) and *p* value are reported. Source data are available online for this figure.

 © The Author(s)

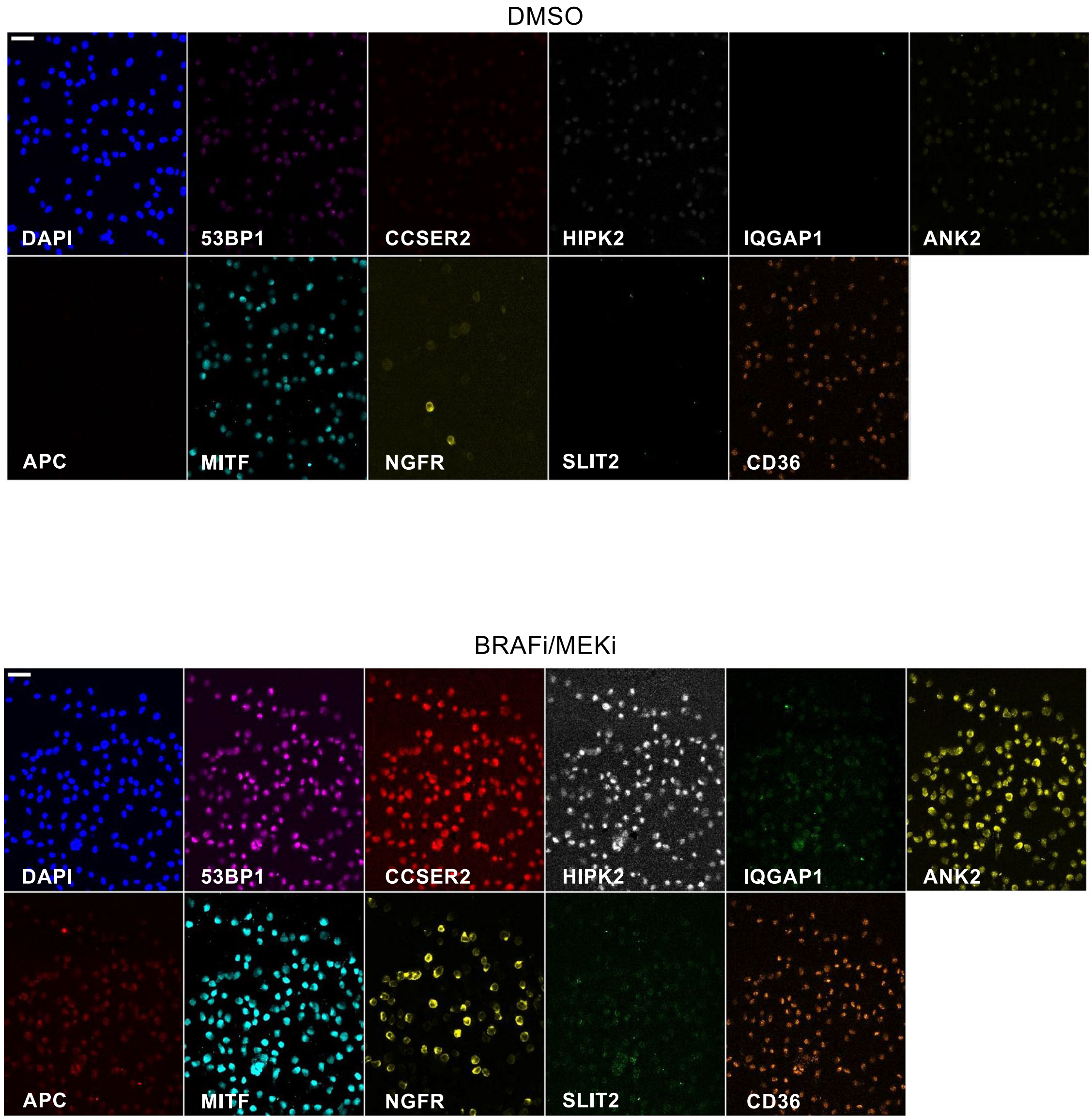

Figure EV3. Single-cell multiplexed immunostaining of translationally and transcriptionally regulated proteins upon targeted therapy.

Representative images of translationally regulated (53BP1, CCSER2, HIPK2, IQGAP1, ANK2, and APC) or transcriptionally regulated (MITF, NGFR, SLIT2, and CD36) protein markers analyzed by multiplex immunostaining (CODEX) in A375 control cells (DMSO) or in cells surviving 3 days of treatment with targeted therapy (BRAFi/MEKi). Scale bar: 50 µm. Source data are available online for this figure.

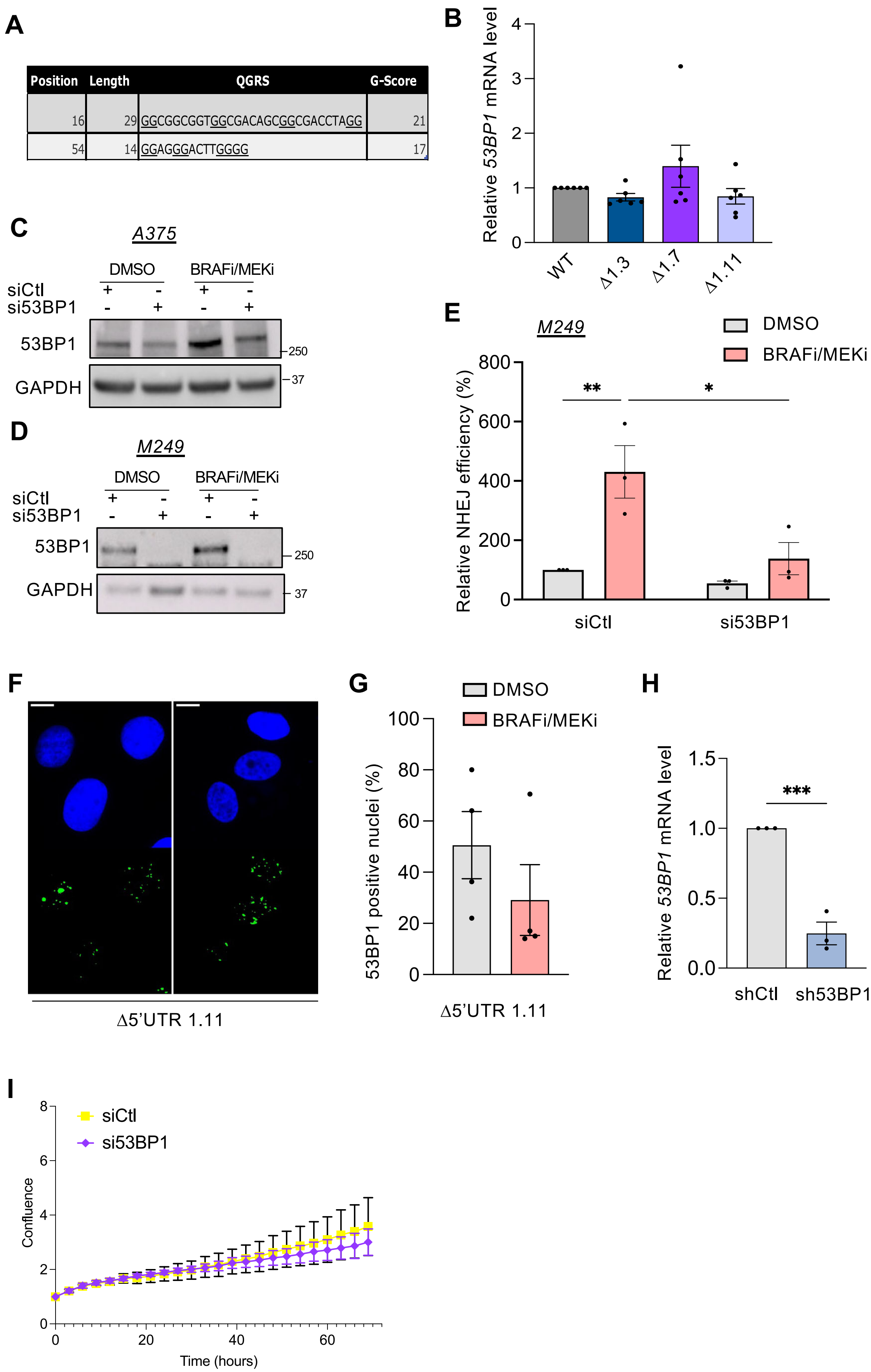
A
Position Length QGRS G-Score
16 29 GGCGGCGGTGGCGACAGCGGCGACCTAGG 21
54 14 GGAGGGACTTGGGG 17
B
Relative 53BP1 mRNA level
WT
Δ1.3
Δ1.7
Δ1.11
C
A375
DMSO
BRAFi/MEKi
siCtl
si53BP1
53BP1
GAPDH
250
37
D
M249
DMSO
BRAFi/MEKi
siCtl
si53BP1
53BP1
GAPDH
250
37
E
M249
DMSO
BRAFi/MEKi
Relative NHEJ efficiency (%)
**
*
siCtl
si53BP1
F
Δ5'UTR 1.11
G
DMSO
BRAFi/MEKi
53BP1 positive nuclei (%)
Δ5'UTR 1.11
H
Relative 53BP1 mRNA level
***
shCtl
sh53BP1
I
siCtl
si53BP1
Confluence
Time (hours)

 © The Author(s)

Figure EV4. Characterization of *53BP1* 5'UTR and 53BP1-dependent phenotypes in drug-tolerant cells.

(**A**) G-quadruplex in the 5′untranslated region of 53BP1 mRNA using QGRS Mapper. (**B**) RT-qPCR quantification of *53BP1* mRNA level in A375 WT cells or in A375 Δ 5′UTR clones. Data represent the mean ± SEM from six independent experiments. Data were normalized using the geometric mean of TBP, Actin, and 18S housekeeping genes. (**C**, **D**) Western blot illustrating 53BP1 protein levels in A375 and M249 control (DMSO) or drug-tolerant cells (BRAFi/MEKi) transfected with control siRNA (siCtl, 25 nM) or with siRNA targeting *53BP1* (si53BP1, 25 nM) corresponding to Figs. 4A and EV4E. GAPDH serves as a loading control. (**E**) Quantification of plasmid integration efficiencies of M249 control (DMSO) and BRAFi/MEKi drug-tolerant cells (BRAFi/MEKi) transfected with control siRNA (siCtl, 25 nM) or siRNA targeting *53BP1* (si53BP1, 25 nM). The mean ± SEM from three independent experiments is shown (two-way ANOVA, $^{**}p = 0.0088$,$^{*}p = 0.0173$). Data were normalized to M249 control cells transfected with siCtl, which were set to 100%. (**F**) Representative images of immunofluorescence in A375 Δ5′UTR cells (clone 1.11) treated with DMSO (control) or in A375 Δ5′UTR cells that survived BRAFi/MEKi-targeted therapy (BRAFi/MEKi), stained for 53BP1 (green) and nuclei (DAPI, blue). Scale bar, 10 µm. (**G**) Quantification of 53BP1 signal in the conditions described in F, analyzed by percentage of cells with ≥10 foci/nucleus. Data represent the mean ± SEM from four independent experiments. (**H**) RT-qPCR quantification of *53BP1* mRNA level in A375 cells stably expressing control shRNA, or shRNA targeting *53BP1*. Data represent the mean ± SEM from three independent experiments (unpaired Student's *t*-test, $^{***}p = 0.0008$). (**I**) Proliferation of A375-R cells following siRNA-mediated depletion of 53BP1, assessed by IncuCyte live-cell imaging. Source data are available online for this figure.

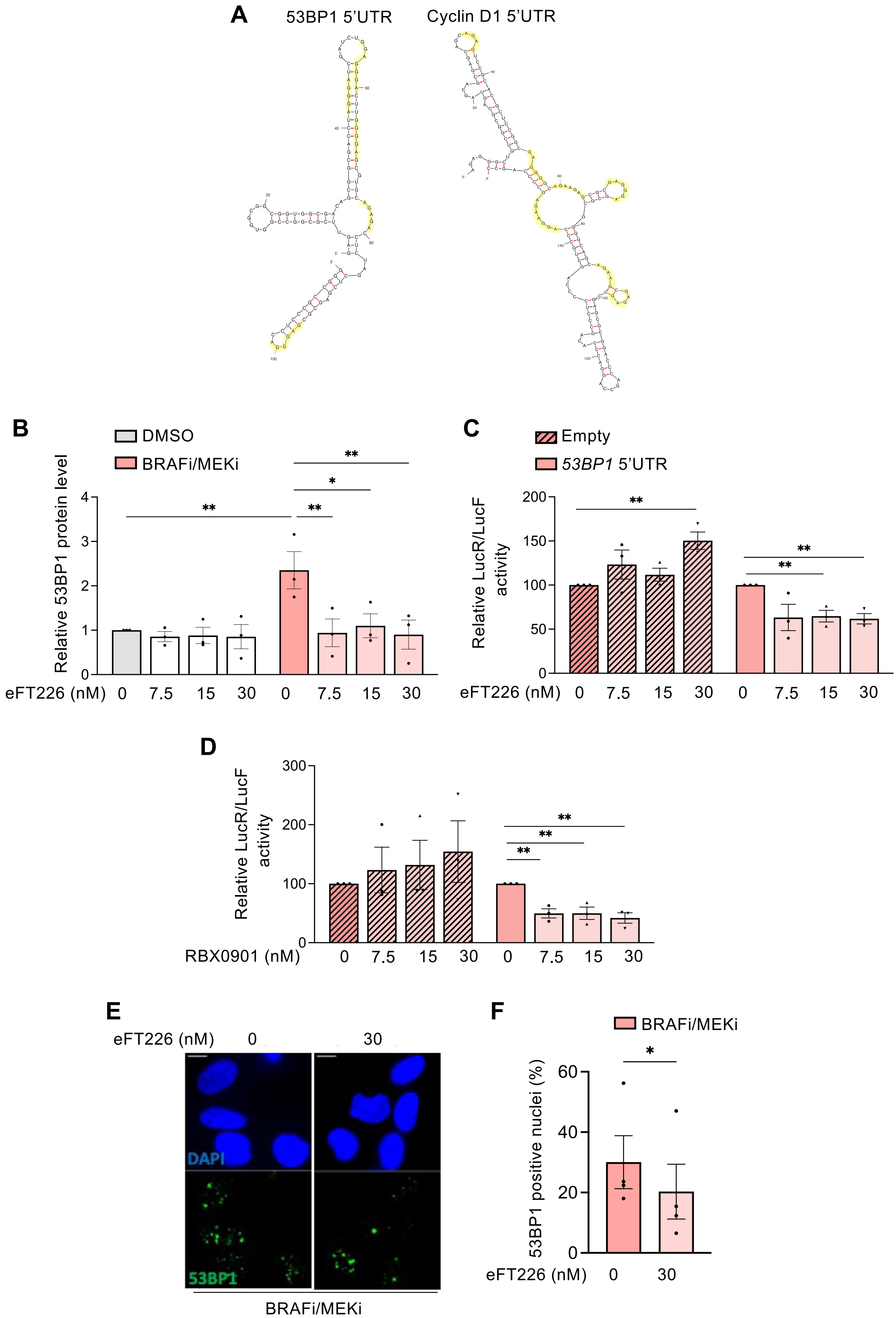
A
53BP1 5'UTR
Cyclin D1 5'UTR
B
DMSO
BRAFi/MEKi
Relative 53BP1 protein level
eFT226 (nM) 0 7.5 15 30 0 7.5 15 30
C
Empty
53BP1 5'UTR
Relative LucR/LucF activity
eFT226 (nM) 0 7.5 15 30 0 7.5 15 30
D
Relative LucR/LucF activity
RBX0901 (nM) 0 7.5 15 30 0 7.5 15 30
E
eFT226 (nM) 0 30
DAPI
53BP1
BRAFi/MEKi
F
BRAFi/MEKi
53BP1 positive nuclei (%)
eFT226 (nM) 0 30

Figure EV5. Pharmacological inhibition of 5'UTR-dependent *53BP1* translation following targeted therapy.

(**A**) Representation of the 53BP1 and Cyclin D1 5′ UTRs using mfold software. Polypurine-rich sequences are highlighted in yellow. (**B**) Relative quantification of 53BP1 protein level in M249 control (DMSO) or drug-tolerant cells (BRAFi/MEKi), treated with the indicated concentration of eFT226 for 24 h. Data represent the mean ± SEM from three independent experiments (two-way ANOVA, BRAFi/MEKi: 0 vs 7.5 ** $p = 0.0056$, 0 vs 15 *$p = 0.0135$, 0 vs 30 **$p = 0.0045$). (**C**) Quantification of the luciferase assay performed in M249 drug-tolerant cells treated with the indicated concentrations of eFT226 for 12 h. Renilla (LucR) activity was measured at the end of the eFT226 treatment, and the activity of the Firefly luciferase (LucF) was used as a control of transfection. Data represent the effect of eFT226 on the luciferase activity of the reporter containing 53BP1 5′UTR or the Empty vector (without 53BP1 5′UTR) and are normalized to the eFT226 untreated conditions. The mean ± SEM from three independent experiments is reported (unpaired Student's *t*-test, 53BP1: 0 vs 7.5 $p = 0.0692$, 0 vs 15 **$p = 0.0060$, 0 vs 30 **$p = 0.0030$). (**D**) Quantification of the luciferase assay performed in M249 drug-tolerant cells treated with the indicated concentrations of RBX0901 for 12 h. Data represent the effect of RBX0901 on the luciferase activity of the reporter containing 53BP1 5′UTR or the Empty vector (without 53BP1 5′UTR) and are normalized to the RBX0901 untreated conditions. The mean ± SEM from three independent experiments is reported (unpaired Student's *t*-test, 53BP1: 0 vs 7.5 **$p = 0.0029$, 0 vs 15 **$p = 0.0088$, 0 vs 30 **$p = 0.0027$). (**E**) Representative images of immunofluorescence in A375 drug-tolerant cells that survived BRAFi/MEKi-targeted therapy (BRAFi/MEKi), treated with or without 30 nM eFT226 for 24 h and stained for 53BP1 (green) and nuclei (DAPI, blue). Scale bar, 10 μm. (**F**) Quantification of 53BP1 signal of the conditions in E, analyzed by percentage of cells with ≥10 foci/nucleus. Data represent the mean ± SEM from four independent experiments. *p* values were calculated by paired, two-tailed Student's *t*-test (*$p = 0.0387$). Source data are available online for this figure.

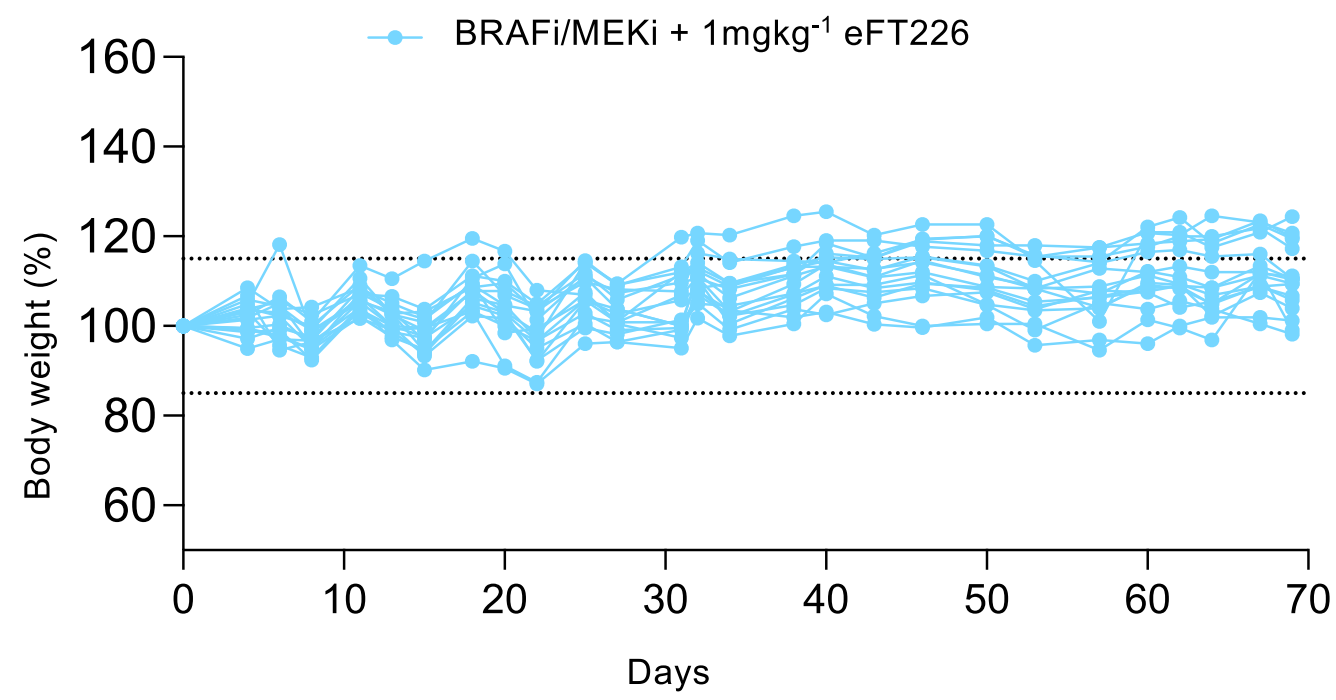

Figure EV6. Tolerability of combined BRAFi/MEKi and eFT226 treatment in mice.

Graph showing the percentage variation in body weight of mice after 70 days of treatment with PLX4720 (BRAFi) and PD0325901 (MEKi), combined with eFT226 (1 mg/kg). Source data are available online for this figure.

 © The Author(s)

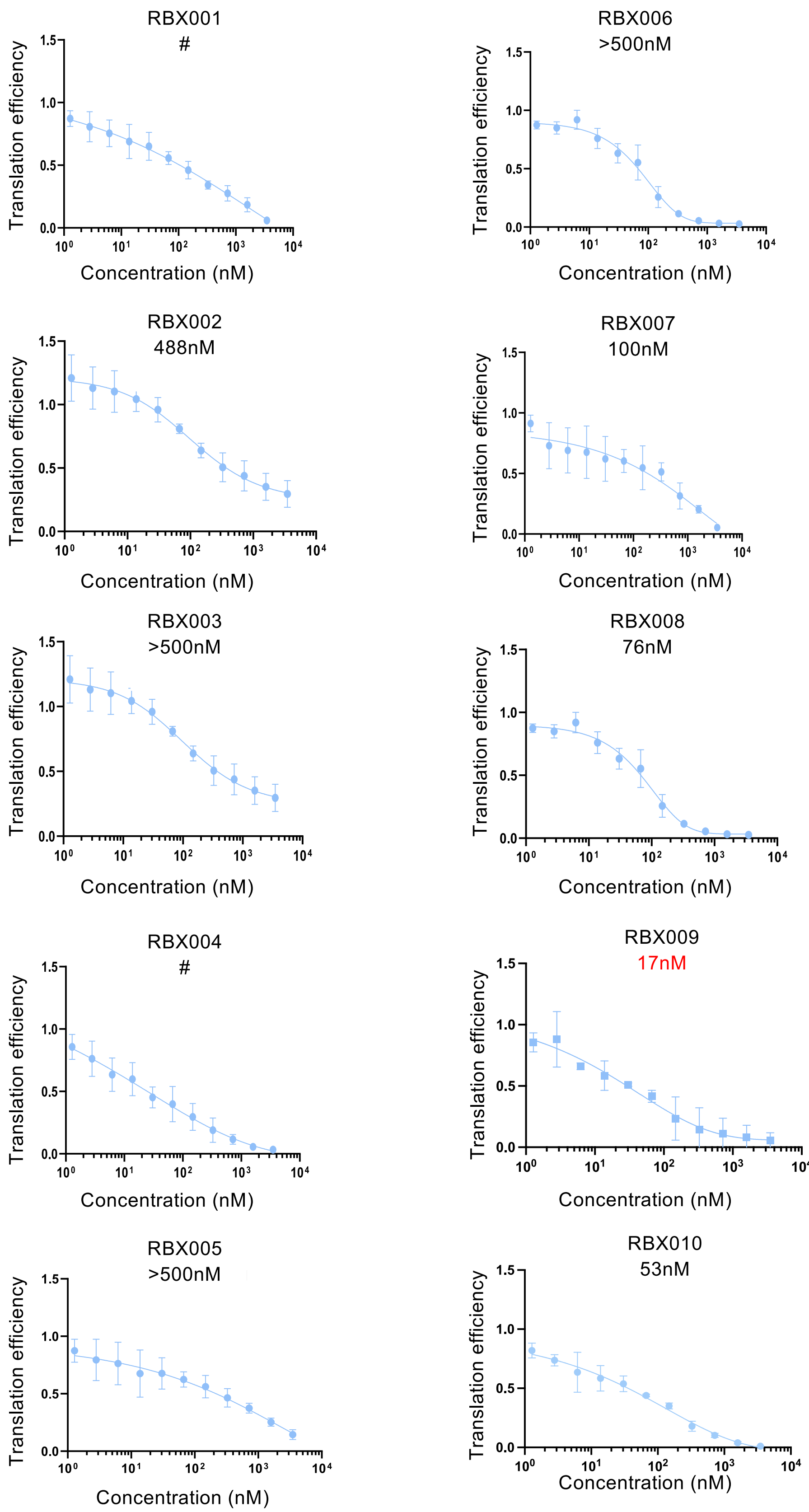

Figure EV7. In vitro screening of flavagline derivatives.

Screening of flavagline-derivative compounds was performed by assessing the in vitro translation of the $(AG)_{10}$ luciferase mRNA reporter in rabbit reticulocyte lysate treated with increasing concentrations of compounds for 1.5 h. Where applicable, the EC_{50} (in nM) is indicated for each compound. Source data are available online for this figure.

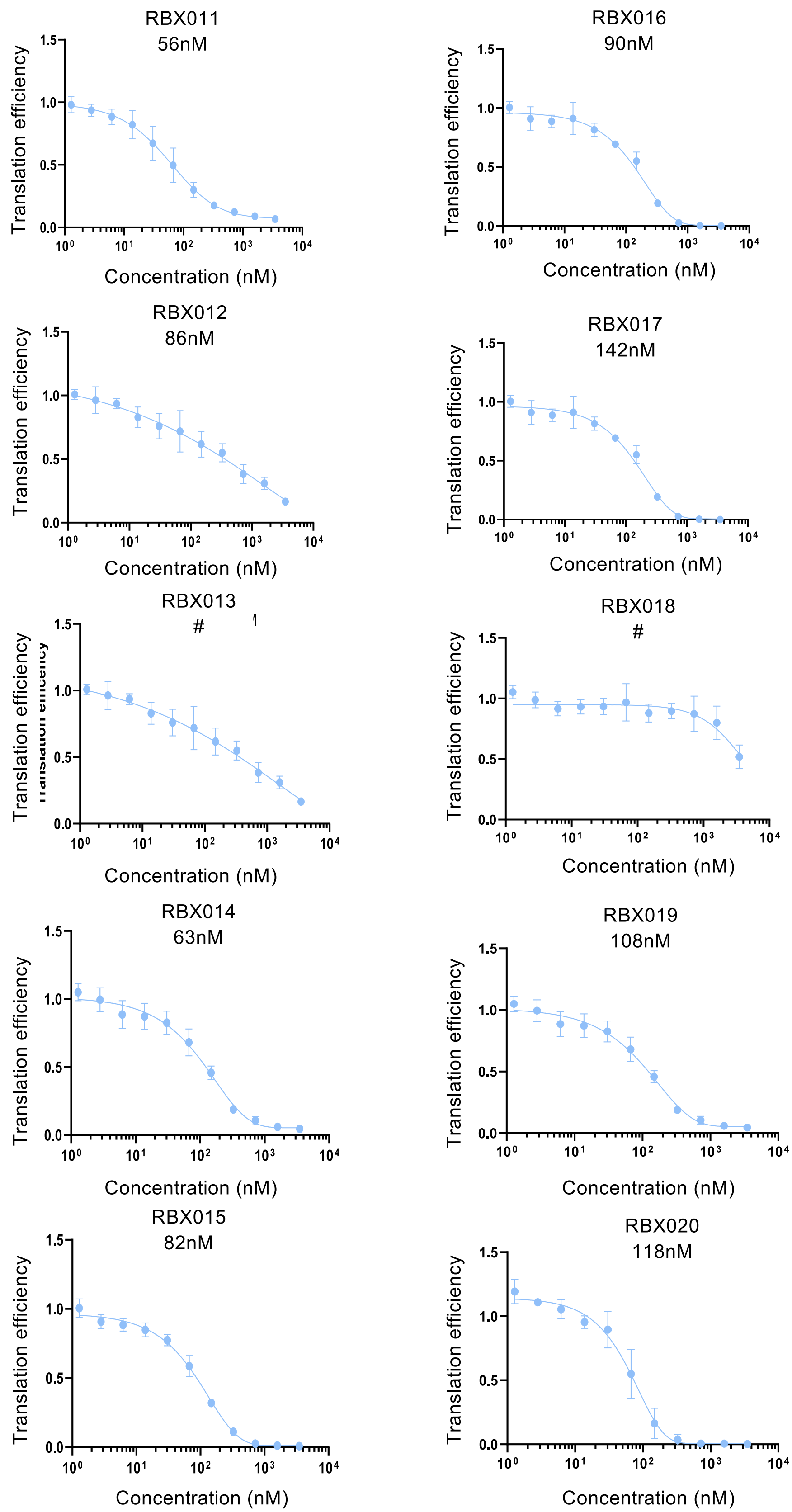

Figure EV7. Continued.

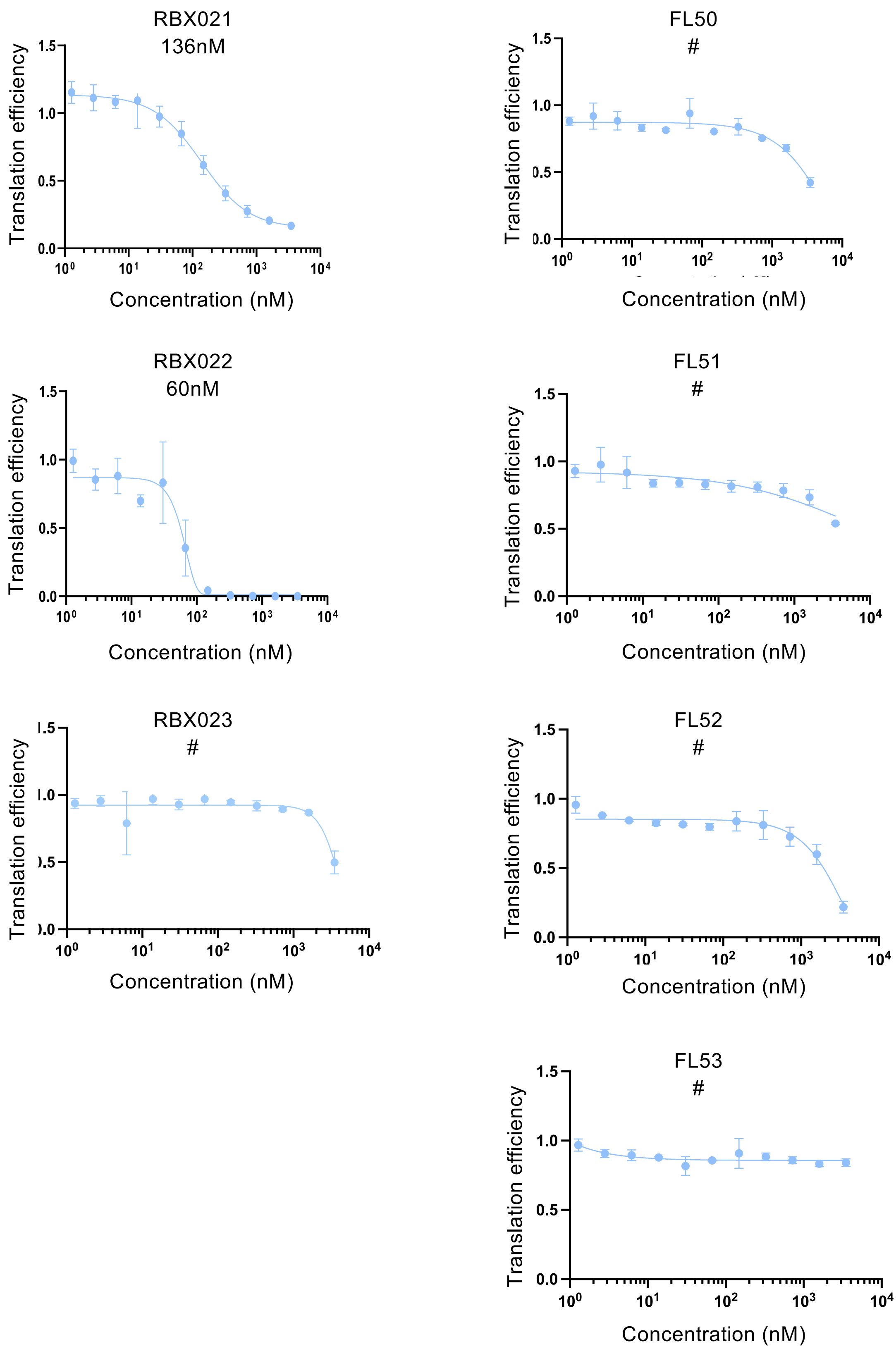

Figure EV7. Continued.

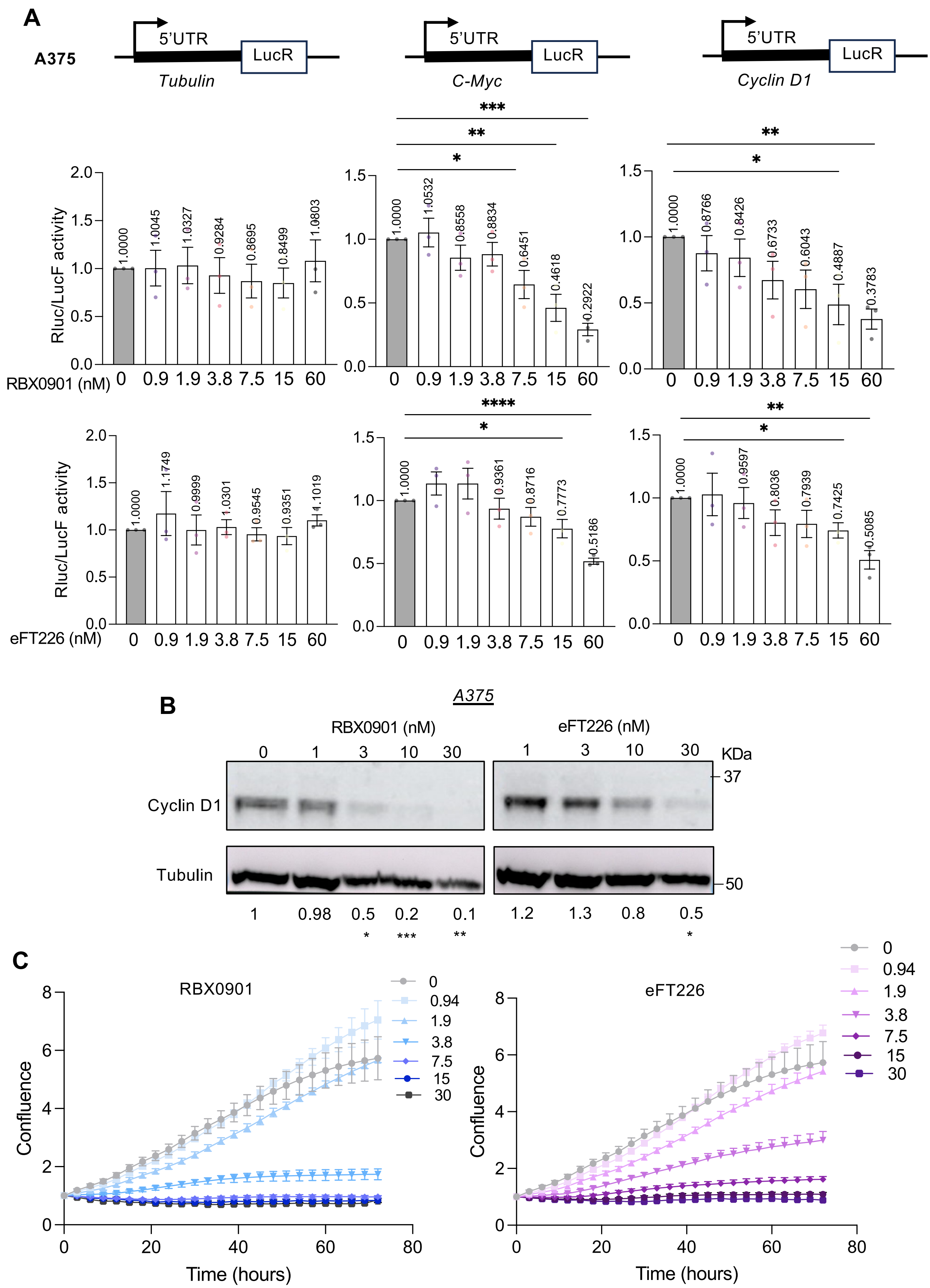
A
A375
5'UTR
LucR
Tubulin
C-Myc
Cyclin D1
Rluc/LucF activity
RBX0901 (nM)
0 0.9 1.9 3.8 7.5 15 60
1.0000 1.0045 1.0327 0.9284 0.8695 0.8499 1.0803
1.0000 1.0532 0.8558 0.8834 0.6451 0.4618 0.2922
1.0000 0.8766 0.8426 0.6733 0.6043 0.4887 0.3783
eFT226 (nM)
1.0000 1.1749 0.9999 1.0301 0.9545 0.9351 1.1019
1.0000 0.9361 0.8716 0.7773 0.5186
1.0000 0.9597 0.8036 0.7939 0.7425 0.5085
B
A375
RBX0901 (nM)
eFT226 (nM)
0 1 3 10 30
1 3 10 30
KDa
37
50
Cyclin D1
Tubulin
1 0.98 0.5 0.2 0.1 1.2 1.3 0.8 0.5
C
RBX0901
eFT226
Confluence
Time (hours)
0 0.94 1.9 3.8 7.5 15 30

Figure EV8. Comparison of eFT226 and RBX0901 effects on 5' UTR-dependent reporter translation inhibition and cell proliferation.

(**A**) (top) Schematic representation of luciferase reporters containing the 5′UTRs of *Tubulin*, *c-MYC*, and *Cyclin D1*, used to monitor the effect of eIF4A inhibitors on reporter translation *in cellulo*. (bottom) Quantification of the luciferase assay performed in A375 cells treated with the indicated concentrations of RBX0901 or eFT226 for 24 h. Renilla (LucR) activity was measured 48 h after transfection, and the activity of the Firefly luciferase (LucF) was used as a control of transfection. Data were normalized to the untreated conditions and represent the mean ± SEM from three independent experiments (unpaired Student's *t*-test, RBX0901: c-MYC: 0 vs 7.5 *$p = 0.0324$, 0 vs 15 **$p = 0.0072$, 0 vs 60 ***$p = 0.000$ 1, Cyclin D1: 0 vs 15 *$p = 0.0286$, **$p = 0.0025$ eFT226: c-MYC: 0 vs 15 *$p = 0.0383$, ****$p \leq 0.0001$, Cyclin D1: 0 vs 15 *$p = 0.0133$, **$p = 0.0012$). (**B**) Western blot illustrating Cyclin D1 protein levels in A375 cells treated with the indicated concentration of RBX0901 or eFT226 for 24 h. Tubulin serves as a loading control, and the quantification relative to untreated conditions from three independent experiments is indicated. *p* values were calculated by unpaired, two-tailed Student's *t*-test (RBX0901: 0 vs 3 *$p = 0.0498$, 0 vs 10 ***$p = 0.0001$, 0 vs 30 **$p = 0.0012$; eFT226: 0 vs 30 *$p = 0.0376$). (**C**) Cell proliferation assay performed in A375 cells treated with the indicated concentrations of RBX0901 or eFT226 for 72 h. Data represent the mean ± SEM from three independent experiments. Source data are available online for this figure.

© The Author(s)

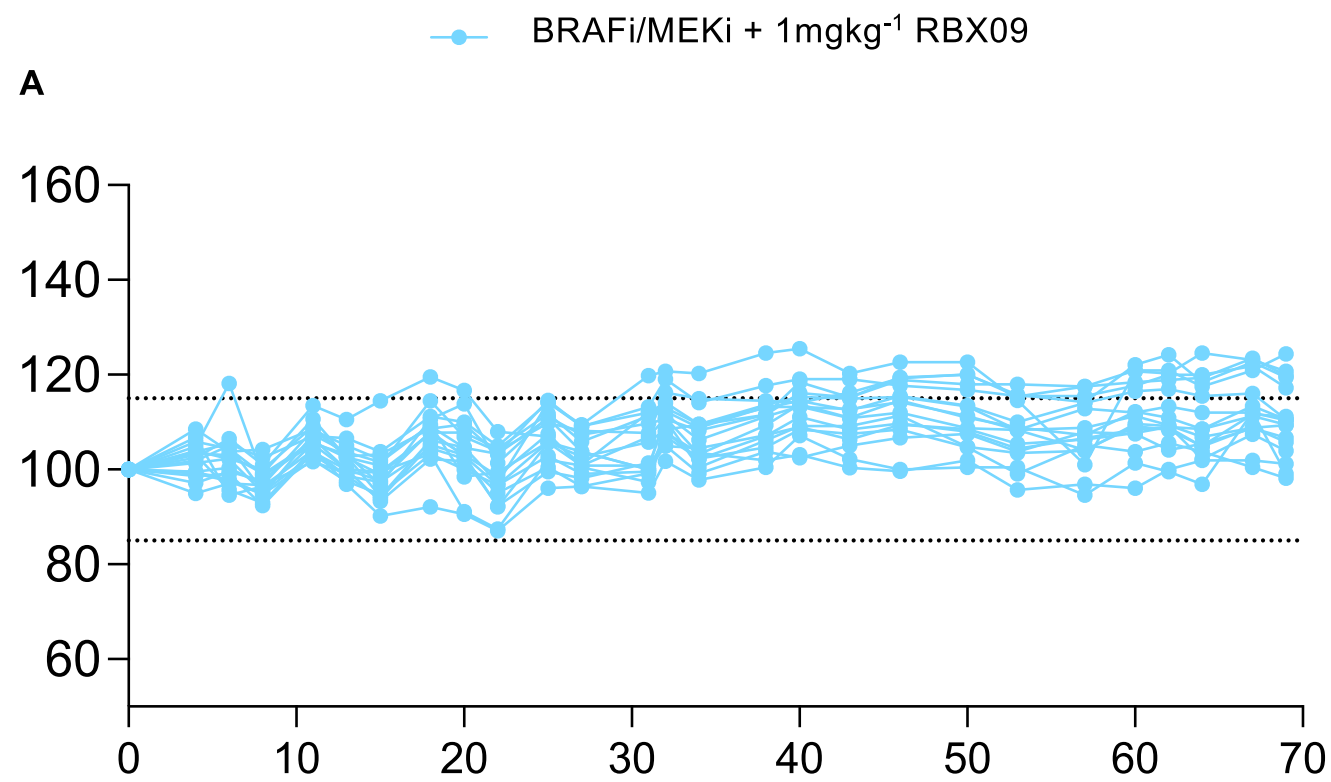

Figure EV9. Tolerability of combined BRAFi/MEKi and RBX0901 treatment in mice.

Graph showing the percentage variation in body weight of mice after 70 days of treatment with PLX4720 (BRAFi) and PD0325901 (MEKi), combined with RBX0901 (1 mg/kg). Source data are available online for this figure.

 © The Author(s)
