# Supplementary material for: Selective mRNA translation determines adaptative mutability of melanoma cells to anti-BRAF/MEK combination therapy
Source: EMBO Mol Med. 2026 Jul 20;18(8):3278–317. doi: 10.1038/s44321-026-00479-5 (PMC13470486; doi:10.1038/s44321-026-00479-5)
Supplement: Supplementary file 2 [file 44321_2026_479_MOESM4_ESM.pdf]

EMBO Molecular Medicine

Selective mRNA translation determines adaptative mutability of melanoma cells to targeted therapy

Stephan Vagner, Lucilla Fabbri, Lucie Lagadec, Eva Guérin, Hélène Lecourt, Dorothée Baille, Laetitia Besse, Cédric Messaoudi, Laurent Désaubry, Hussein Abou-Hamdan, Sévérine Roy, Bérangère Lombard, Damarys Loew, Jean-Yves Scoazec, and Caroline Robert

Corresponding authors: Stephan Vagner (Stephan.Vagner@curie.fr) , Lucilla Fabbri (Lucilla.fabbri@curie.fr)

Review Timeline:

Submission Date:	9th Oct 25
Editorial Decision:	12th Nov 25
Revision Received:	7th Apr 26
Editorial Decision:	11th May 26
Revision Received:	3rd Jun 26
Accepted:	19th Jun 26

Editor: Lise Roth

Transaction Report:

(Note: With the exception of the correction of typographical or spelling errors that could be a source of ambiguity, letters and reports are not edited. Depending on transfer agreements, referee reports obtained elsewhere may or may not be included in this compilation. Referee reports are anonymous unless the Referee chooses to sign their reports.)

12th Nov 2025

Dear Dr. Vagner,

Thank you for the submission of your manuscript to EMBO Molecular Medicine, and please accept my apologies for the delay in getting back to you as we discussed your manuscript further with the referees after receiving their reports.

As you will see from the enclosed reports, while referees #1 and #3 are overall supportive of the study pending necessary revisions and clarifications, referee #2 raises several concerns that challenge the conclusions and interpretation of the results (including arbitrary definition of persister cells and alternative scenario in which 53BP1 upregulation is primarily transcriptional). As mentioned above, we further cross-commented with the referees. Based on their feedback and after discussion with my colleagues, considering also the amount of work required for adequate revisions, we prefer to return the manuscript to you at this point with the decision that we cannot offer to publish it. However, given the potential interest of the findings, we would consider a new manuscript on the same topic if you could convincingly confirm exclusion of transcriptional effects and clarify and discuss the limitations linked to the definition of persister cells.

To be completely clear, however, I would like to stress that if you were to send a new manuscript, this would be treated as a new submission rather than a revision and would be reviewed afresh, in particular with respect to the literature and the novelty of your findings at the time of resubmission. If you decide to follow this route, please make sure you nevertheless upload a letter of response to the referees' comments.

I am sorry that I could not bring better news this time and hope that the referees' comments are helpful in your continued work in this area.

With kind regards,

Lise Roth

Lise Roth, Ph.D
Senior Editor
EMBO Molecular Medicine

***** Reviewer's comments *****

Referee #1 (Comments on Novelty/Model System for Author):

The authors use a melanoma xenograft model where mice are treated with BRAF/MEK inhibitors to induce tumor relapse. The model is adequate to test melanoma tumor persistence and resistance to treatment.

Referee #1 (Remarks for Author):

In this manuscript, Vagner, Roberts and colleagues show that 53BP1 mRNA translation is important for NHEJ and mutability of cells upon BRAFi/MEKi treatment. They further show that translation enhancement upon drug exposure is mediated by 53BP1 5' UTR and by the translation helicase eIF4A, which can be inhibited by eFT226 (Zotatifin, an FDA approved drug). The authors obtain derivatives of Zotatifin that display higher activity in translational repression of mRNAs containing polypurine 5' UTRs and that, as Zotatifin, reduce the emergence of BRAFi/MEKi resistance. Altogether, the experiments are well performed and the data support the conclusions. The study is important because it reveals a non-transcriptional mechanism (i.e. translation regulation) as a central mechanism for the acquisition of drug resistance.
The manuscript will improve by the addition of further data as follows:
1) Cell mutability eventually leads to BRAFi/MEKi resistance and tumor relapse. Presumably, upon appearance of stable resistance cells should be insensitive to 53BP1 levels, and hence to 53BP1 translational control. Can the authors show that deletion of 53BP1 5' UTR, or depletion of 53BP1 altogether, does not affect proliferation of BRAFi/MEKi drug resistant cells? Can they show that eIF4A inhibition does not affect survival of BRAFi/MEKi resistant cells?
2) The authors analyze polysome profiling data from a previous paper and select 53BP1 mRNA as their favorite target for this paper. Which other factors involved in NHEJ are translationally regulated by BRAFi/MEKi? Why did they select 53BP1 mRNA?
3) Fig 1G: Three patients are almost anecdotal. Although I understand that this might not be easy, the manuscript would gain by adding more patients.
4) Figs 2F and S2D: It is difficult to see that increase in 53BP1 or IQGAP1 translation upon BRAFi/MEKi treatment occurs only in a subset of cells. Furthermore, in Fig S2D the signal increase is not evident. Please, show a wider view of the microscopy

images to better appreciate these differences.
5) Fig 2K: What do numbers on the left of the heat plot indicate and what is being measured in the scale (mass spectrometry spectral areas?). The authors argue for 'selective translational regulation' in the main text, but there is no RIBOmap measurement of genes that are not translationally increased. Showing RIBOmap of such genes would be required to make this claim. The authors could show RIBOmap of mRNAs that are neither transcriptionally nor translationally regulated, or of mRNAs encoding HDR factors.
6) Fig 3D: Why did the authors select this region of the 5' UTR? Does it include strong secondary structure? Can the authors show a scheme of 53BP1 5' UTR features, including the polypurine motifs that make it sensitive to eIF4A inhibition? How do you presume that eIF4A regulates translation of 53BP1 mRNA?
7) Fig S4A should be moved to main Figures and Fig 3E can go to Supplementary Figures.
8) Fig 7D: Translation of the empty construct seems to increase in a dose-dependent manner. Why is this?
9) Legends of Figures S3 and S4 seem to be swapped.

Referee #2 (Remarks for Author):

Fabbri et al. investigate the molecular mechanisms underlying the survival of melanoma cells exposed to combined BRAF and MEK inhibitors (BRAF/MEKi), focusing on a subpopulation of drug-tolerant "persister" cells. The authors propose that these persisters undergo a drug-induced translational reprogramming, leading to selective upregulation of 53BP1, a key DNA repair protein. They argue this promotes error-prone non-homologous end joining (NHEJ), thereby accelerating mutagenesis and facilitating the eventual emergence of genetically resistant clones. However, several critical issues challenge the strength and interpretation of their conclusions.

Major concerns

1. The study defines persisters as the 30% of cells surviving a 3-day treatment with BRAF/MEK inhibitors. However, this definition appears arbitrary and lacks biological consistency. Survival rates are highly dependent on drug dose and duration: longer or more intense treatment could reduce survival to 5%, while shorter exposure might leave 80% alive. Without comparative molecular profiling across these different survival thresholds, it is unclear whether the "30% persisters" represent a distinct, stable cell state or simply reflect stochastic survival due to variable drug sensitivity.
Moreover, the functional relevance of this population to clinical persistence is uncertain. The link to human IHC data (Fig 1C) is tenuous-no scoring methodology is provided, and there is no evidence that the tumor samples contain true persister cells rather than heterogeneous or transiently stressed cells. The lack of a standardized, biologically grounded definition weakens the model's translational significance.
Notably, 53BP1 upregulation is not uniform across all persisters (Fig 1K), suggesting it is not a core, defining feature of the persister state but rather a variable response in a subset of cells. This heterogeneity further undermines claims of a coherent, programmed translational response.

2. A major alternative explanation is not sufficiently addressed: that 53BP1 upregulation is primarily transcriptional, driven by DNA damage rather than selective translation. BRAF/MEK inhibitors are known to induce replication stress and double-strand breaks (DSBs), activating the DNA damage response (DDR), which includes transcriptional upregulation of DDR genes such as 53BP1.
The authors claim no change in 53BP1 mRNA levels, but the qPCR data in Fig S1C show a 1.5- to 2-fold increase in 4 out of 7 conditions-changes that, while modest, may be biologically significant, especially given the limitations of qPCR in detecting subtle but consistent transcriptional shifts (PMID: 12184808). The absence of more rigorous quantification leaves open the possibility that transcriptional induction is occurring.
In this alternative model, global translation is suppressed (due to eIF4F inhibition via ERK/mTOR blockade), but certain stress-induced mRNAs-like 53BP1-are still translated. Mutating 53BP1 or inhibiting eIF4A would then impair survival not because of disrupted translational reprogramming, but because cells fail to mount an essential DDR. This would explain the observed dependency on 53BP1 for long-term resistance and relapse in vivo-without invoking a novel, selective translational program.
The distinction between translational reprogramming and stress-induced transcription is crucial. The authors, experts in translational control, may be predisposed to interpret data through this lens, but they must more rigorously exclude the more parsimonious transcriptional model.
3. The evidence for translational control of 53BP1 relies heavily on indirect and technically limited assays. AHA labeling and Ribomap are used to infer translation, but Ribomap, in particular, is not a well-validated or widely adopted method. The punctate signals interpreted as translation sites require stronger validation-for example, disappearance upon cycloheximide treatment to confirm active translation. In addition, more standard approaches, such as polysome profiling with qPCR of 53BP1 mRNA across sucrose gradient fractions should be used.

4. In Figure 3, the authors use a dual-luciferase reporter to assess the translational activity of the 53BP1 5'UTR. However, the data are normalized to an empty vector control, despite the R/F (Renilla/Firefly) ratio inherently correcting for transfection efficiency and transcriptional activity. This additional normalization may artificially amplify differences and obscure true effects. The rationale for this approach is not explained, raising concerns about data interpretation.

5. The CRISPR-mediated deletion of nucleotides 41-95 in the 53BP1 5'UTR lacks a clear rationale. No prior functional mapping (e.g., deletion series or mutagenesis) is presented to justify targeting this specific region. A more systematic approach-such as scanning the 5'UTR for regulatory elements via reporter assays-should be performed.
Furthermore, the authors claim that the deletion does not affect 53BP1 mRNA levels, yet Fig 3E shows clear mRNA reduction in two of three clones, and Fig 3G also indicates mRNA changes. This discrepancy is problematic. Given that the 5'UTR overlaps with a strong H3K27ac mark-a chromatin signature of enhancers or promoters-this region may influence transcription. Disrupting it could therefore affect mRNA levels independently of translation, confounding the interpretation of phenotypic effects.

Referee #3 (Remarks for Author):

In this manuscript, Fabbri and colleagues propose that selective mRNA translation determines adaptive mutability of drug-tolerant persister (DTP) melanoma cells to MAPK-targeted therapy (BRAF+MEK inhibitors). Short-term BRAF+MEKi treatment induces translation of mRNA encoding 53BP1, a component of the error-prone non-homologous end joint machinery (NHEJ), while reducing levels of RAD51, a component of the error-free homology-directed repair (HDR) pathway. Authors validate the increase of 53BP1 protein levels in a small cohort of paired baseline/on-treatment patient samples. Authors describe that NHEJ efficiency and mutability upon BRAF+MEKi depend on 53BP1, and that direct inhibition of the 5'-UTR-driven translation of 53BP1 mRNA is sufficient to impair NHEJ and mutability.

To translate these findings into a preclinical setting, authors focus on inhibiting the eIF4A RNA helicase, which is implicated in protein translation, with a flavagline-derivative and clinically relevant inhibitor eFT226 or a synthetic flavagline derivative. These two compounds reduce the emergence of DTPs in vitro, presumably by reducing their mutability. In vivo, co-targeting eIF4A and BRAF+MEKi delays development of resistance in A375 xenografts.

This is an important study that sheds light into DTP biology and adaptive mechanisms to MAPK-targeted therapy, as well as identifying compounds that, when co-targeted with BRAFMEKi, reduce DTP emergence and delay acquired resistance to BRAFMEKi.

There a few limitations that should be highlighted or better revised with additional models.

1.Although figs 1-4 show results in both A375 and M249 cell lines, some results in other figures, in particular regarding NHEJ efficiency and eIF4A inhibitors, were carried out only in A375 cells. Further validation in additional cell lines would strengthen these conclusions.

2.Most of the conclusions are supported by solid and well replicated data. However, there are some instances with large experimental variability in some key experiments, and authors reach to certain conclusions that may need revision/clarification. For example, authors conclude that 53BP1 mRNA does not increase upon BRAFMEKi treatment (Fig. S1C), but it looks like there is a trend towards an increase of mRNA. Off the 4 replicates, there is 1 with fold-change larger than 2, another with 1.7, another 1.4 and another 0.5. Did 53BP1 protein change to a similar extent in those 4 experiments? Fig.1E that shows 53BP1 protein levels by western blot has 3 replicates.

Similar comment for other graphs:
-Fig.3E. Analysis of 53BP1 transcript levels in 3 different clones indicated that the 5'UTR deletion did not affect promoter functions (Fig. 3E). However, and although maybe not statistically significant, there is a reduction in 53BP1 mRNA in some replicates of D1.3 and D1.11.
-Fig.3G. authors conclude that mRNA is not affected, but there is a lot of variability, and in D1.3 and D1.11 there are several replicates with a 2-fold change increase in mRNA level, in particular for D1.3.
-Fig. S4C. 53BP1+ nuclei in the D5'UTR1.11, there is a lot of variability, but still there are 3 replicates that show almost 2-fold reduction vs DMSO average. Also, here one dot of the DMSO is almost hidden by the red box of BRAFiMEKi label.

-Fig.5e. there are still important decreases (up to 50%) of lucR/lucF in Empty, not to dissimilar to some replicates in the 53BP1 5'UTR.

Additional points that should be clarified/discussed:

4. Fig.3B. what are the first 3 bars vs the last 3??

5. Fig.3F some of these blots should be improved, in particular for D1.7 and D1.11.

6. Fig. S8. "Additionally, RBX0901 tended to be more effective than eFT226 in reducing the
translation of reporters containing the 5′UTRs of c-Myc and Cyclin D1,". This conclusion as is requires a statistical analysis side

by side of the decrease of translation.

7. Fig 6A-B and 7E-F. Did authors test that the eIF4A inhibitor is acting on-target in vivo? For example, by assessing 53BP1 levels in the treated tumours. According to their in vitro data (Fig.5a), 53BP1 should increase with BRAFMEKi and decrease in the eFT226+BRAFMEKi-treated tumours.

8. Although methods say that for multiple comparisons ANOVA was used, there are a few graphs with several groups being analysed by t-test. For example, fig.1F, 4F..

As a service to authors, EMBO provides authors with the possibility to transfer a manuscript that one journal cannot offer to publish to another EMBO publication. The full manuscript and if applicable, reviewers reports are automatically sent to the receiving journal to allow for fast handling and a prompt decision on your manuscript. For more details of this service, and to transfer your manuscript to another EMBO title please click on Link Not Available

Please do not share this URL as it will give anyone who clicks it access to your account.

EMM-2025-22756-V2-Q
Comments to the reviewers in black

Referee #1 (Remarks for Author):

1) Cell mutability eventually leads to BRAFi/MEKi resistance and tumor relapse. Presumably, upon appearance of stable resistance cells should be insensitive to 53BP1 levels, and hence to 53BP1 translational control. Can the authors show that deletion of 53BP1 5' UTR, or depletion of 53BP1 altogether, does not affect proliferation of BRAFi/MEKi drug resistant cells?

We thank the reviewer for this suggestion. We assessed the impact of 53BP1 depletion in a model of acquired resistance. Specifically, A375 cells were rendered resistant to BRAFi/MEKi through prolonged exposure to escalating doses (A375-R; Fig. R1A below). siRNA-mediated depletion of 53BP1 did not affect the proliferation of A375-R cells as measured by real-time Incucyte experiments (Fig. R1B below), supporting the reviewer's hypothesis.

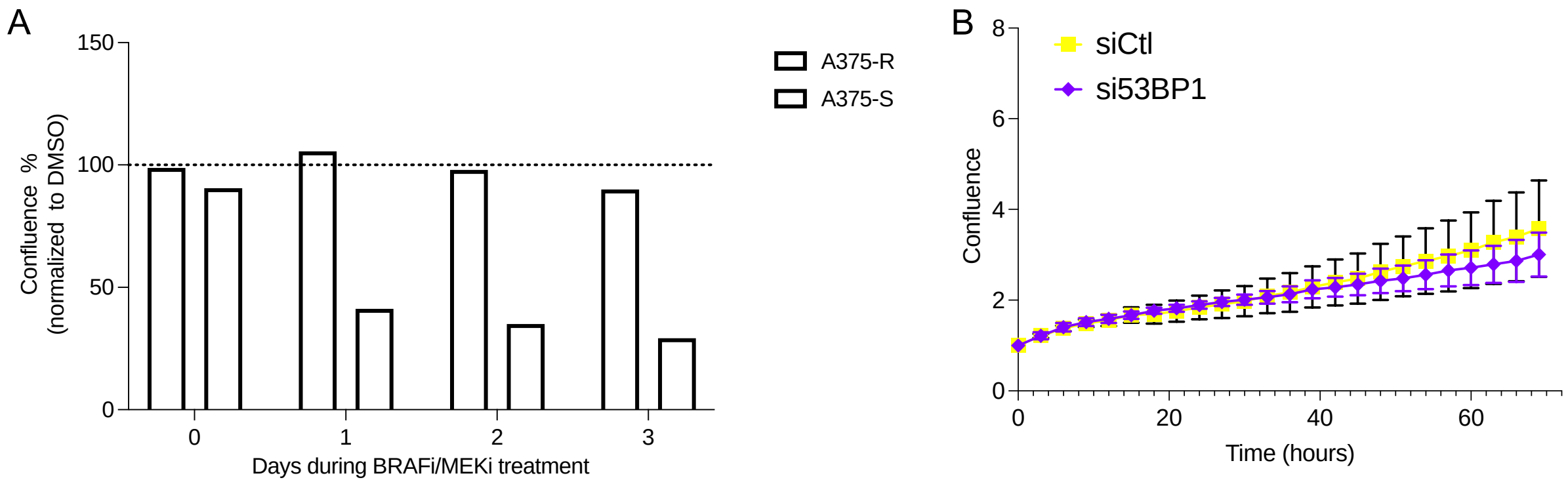

Figure R1: A. Proliferation of parental (A375-S) and BRAFi/MEKi-resistant (A375-R) A375 cells during treatment with BRAFi/MEKi (200 nM each), monitored by Incucyte live-cell imaging. Data are presented relative to DMSO-treated controls. **B.** Proliferation of A375-R cells following siRNA-mediated depletion of 53BP1, assessed by Incucyte live-cell imaging.

A broader investigation of 53BP1 function and DNA damage response rewiring in resistant populations would be of interest. However, such analyses would require substantial additional investigation and are beyond the scope of the present study. However, we would be happy to include these data as supplementary information if the reviewer and editor consider them important for the manuscript.

Can they show that eIF4A inhibition does not affect survival of BRAFi/MEKi resistant cells?

We previously reported (PMID: 25079330) that melanoma cells resistant to anti-MAPK inhibitors depend on the eIF4F complex and are therefore sensitive to its inhibition, including by flavagline derivatives targeting eIF4A. Nonetheless, we repeated the experiment by assessing the proliferation of the afore-mentioned A375-R cell model in the presence of eFT226 and found that A375-R cells remained sensitive to eFT226 treatment (Fig. R2).

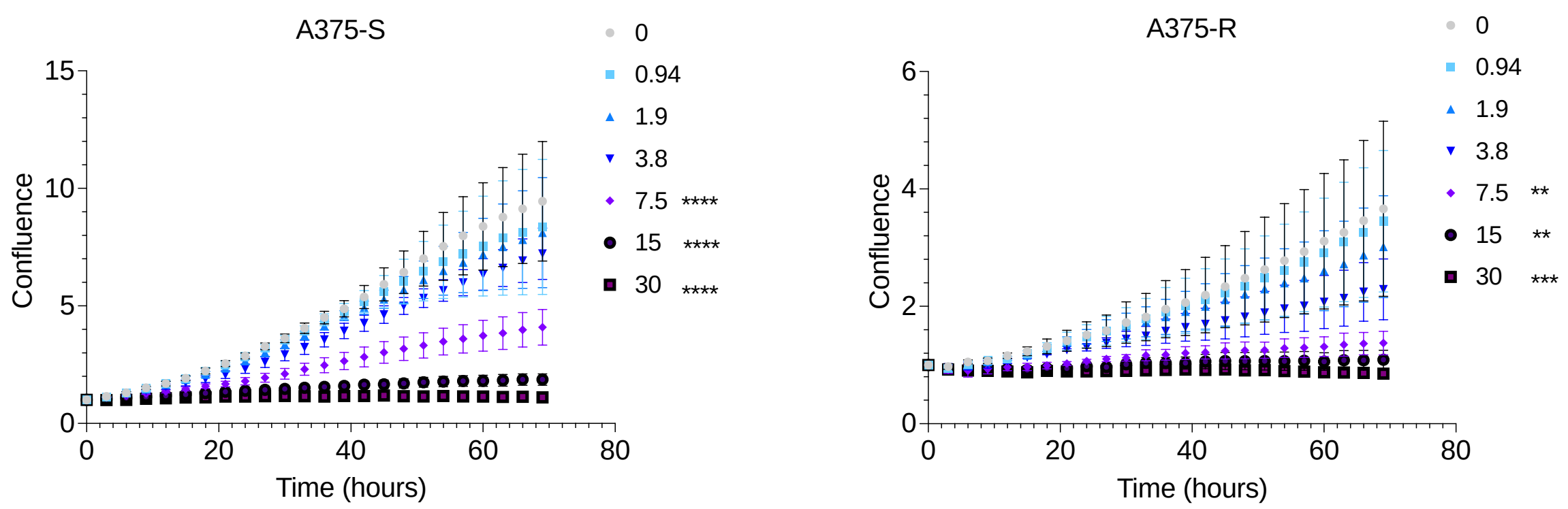

Figure R2 : Proliferation of parental (A375-S) and BRAFi/MEKi-resistant (A375-R) A375 cells during treatment with the indicated concentrations of eFT226 (nM), monitored by Incucyte live-cell imaging. Data are presented relative to T0 (start of the treatment).

These results indicate that the eIF4A-dependent translational program remains functionally relevant in cells with acquired resistance. They further suggest that, beyond 53BP1, additional eIF4A-dependent mRNAs contribute to the translational landscape sustaining proliferation in A375-R cells and are affected by eIF4A inhibition.

2) The authors analyze polysome profiling data from a previous paper and select 53BP1 mRNA as their favorite target for this paper. Which other factors involved in NHEJ are translationally regulated by BRAFi/MEKi? Why did they select 53BP1 mRNA?

Our previously published polysome profiling analysis (PMID: 31844050) revealed a broad translational dysregulation of pathways involved in DNA damage response (Fig. R3 below), a finding that is further supported by the proteomic analysis presented in this study (Fig. 1A). However, among the core NHEJ factors, the 53BP1 mRNA was the only one consistently identified as being upregulated at the translational level in response to BRAFi/MEKi treatment. We did not detect translational changes for mRNAs encoding other NHEJ components in our dataset. We therefore focused on 53BP1 because it emerged as the most robust candidate, showing both increased translational efficiency and a corresponding increase in protein levels, not detected for the NHEJ factors analysed (Fig. S1B).

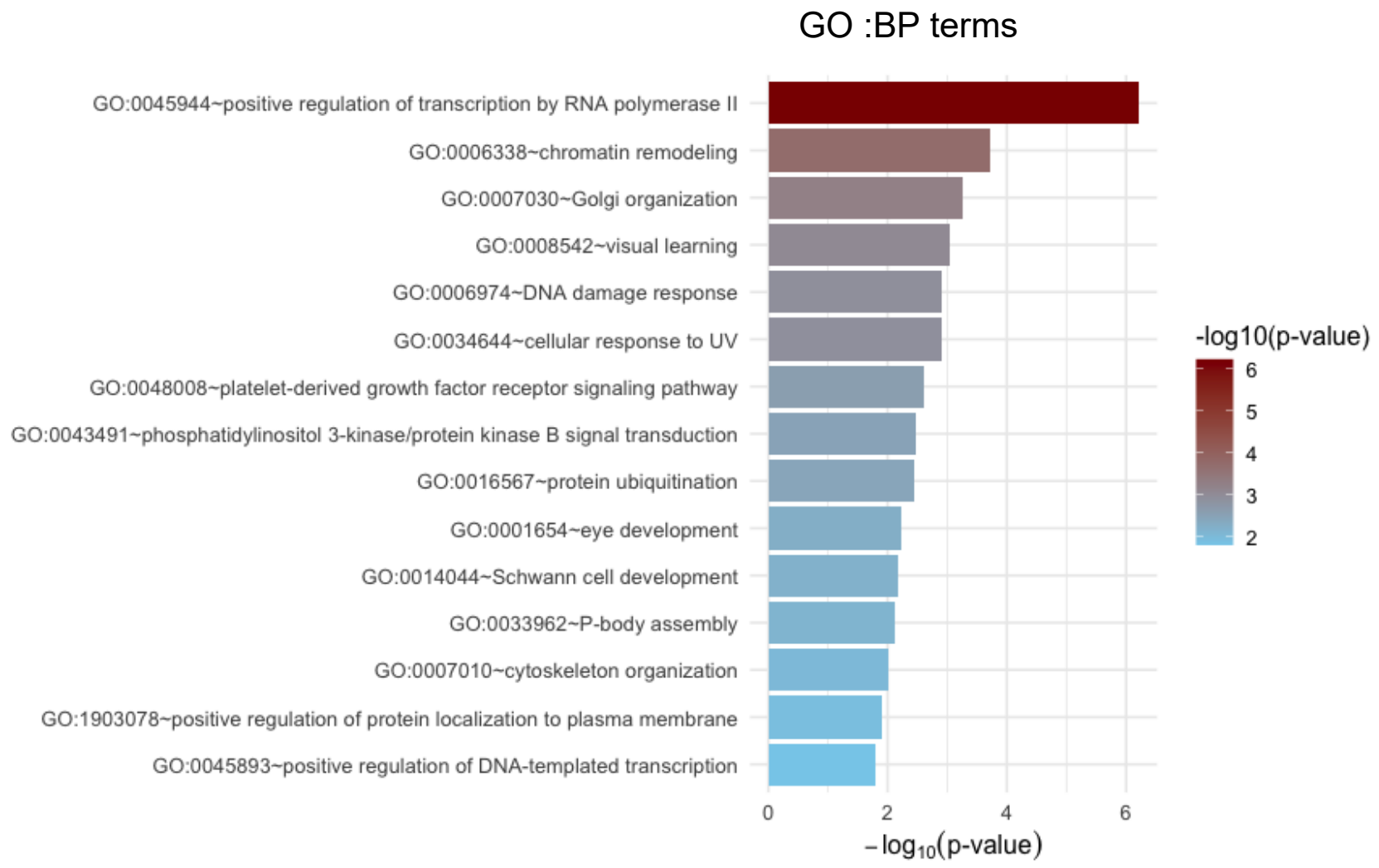

Figure R3: GO:BP terms of the translationally de-regulated mRNAs in PMID: 31844050.

We have added the following sentence in the manuscript (line 138):
"*Among the translationally upregulated mRNAs, we selected the one encoding the key NHEJ mediator 53BP1, which appeared as being the only translationally-regulated mRNA encoding a NHEJ component*"

3) Fig 1G: Three patients are almost anecdotal. Although I understand that this might not be easy, the manuscript would gain by adding more patients.

We fully agree with the reviewer's comment that increasing the number of patient samples would strengthen the study. Initially, we obtained matched baseline and on-treatment biopsies from a slightly larger cohort of patients (n = 7). However, following tumour sectioning and immunohistochemical analyses, only a subset of these samples met the quality criteria required for reliable analysis. Specifically, several biopsies were excluded due to insufficient

tumour cell content, extensive melanin pigmentation, poor tissue preservation, or the presence of necrotic areas that compromised histological interpretation. Despite our efforts, we were unable to obtain additional matched biopsies. We acknowledge this limitation and have indicated it in the revised manuscript line 156, i.e. "*While this observation is based on a limited sample set… »*

4) Figs 2F and S2D: It is difficult to see that increase in 53BP1 or IQGAP1 translation upon BRAFi/MEKi treatment occurs only in a subset of cells. Furthermore, in Fig S2D the signal increase is not evident. Please, show a wider view of the microscopy images to better appreciate these differences.

We thank the reviewer for this comment. Images in Fig. 2F and S2E have been replaced for better interpretation. Confocal images were acquired at 60x magnification with a 2.5x zoom, which provides optimal resolution for RiboMap signal detection. Complete field of view are shown.

5) Fig 2K: What do numbers on the left of the heat plot indicate and what is being measured in the scale (mass spectrometry spectral areas?).

We apologize for the error. The labelling in Fig. 2K has now been added. Specifically, the numbers on the left correspond to the cells analyzed, and the color scale represents the single-cell abundance (mean Intensity) of the reported proteins as measured by CODEX multiplexed imaging. This information is now added in the revised figure legend.

"**K**, *Heatmap generated from multiplexed imaging analysis, displaying, for each cell, single-cell mean intensity values (rows) for protein expression of selected differentially expressed markers (columns), regulated at either the translational or transcriptional level in control cells (DMSO) and drug-tolerant cells (BRAFi/MEKi)*."

The authors argue for 'selective translational regulation' in the main text, but there is no RIBOmap measurement of genes that are not translationally increased. Showing RIBOmap of such genes would be required to make this claim. The authors could show RIBOmap of mRNAs that are neither transcriptionally nor translationally regulated, or of mRNAs encoding HDR factors.

We thank the reviewer for this suggestion. We decided to assess the translation of the mRNA encoding the HDR factor RAD51 by RiboMap. We found that this mRNA showed reduced association with active ribosomes in drug-tolerant cells (Fig. S2D of the manuscript). This conclusion has been added in the text line 180 "*In contrast, the mRNA encoding the HRD factor RAD51 showed reduced association with active ribosomes (Fig. S2D)"* together with a new Fig. S2D.

6) Fig 3D: Why did the authors select this region of the 5' UTR? Does it include strong secondary structure? Can the authors show a scheme of 53BP1 5' UTR features, including the polypurine motifs that make it sensitive to eIF4A inhibition? How do you presume that eIF4A regulates translation of 53BP1 mRNA?

The 53BP1 5′UTR is 113 nt long and contains two predicted G-quadruplex (G4) structures at positions 16 and 54 (Fig. R4A below and new Fig. S4A). G4 have been described in eIF4A inhibitor-sensitive mRNAs (PMID: 25079319).
To investigate the role of the 53BP1 5′UTR in its endogenous context, we deleted, to the extent technically feasible, a region encompassing the G4 located at position 54. With respect to eIF4A-sensitive polypurine motifs, the 53BP1 5′UTR harbours several purine-rich stretches at a density comparable to that of the well-characterized eIF4A-sensitive CCND1 (NM_053056.3) 5′UTR, which is 158 nt long (Fig. R4B. below). As noted in the Discussion

(line 424), “the precise mechanism by which eIF4A regulates 53BP1 mRNA translation remains to be elucidated. Future studies aimed at identifying additional trans-acting factors that cooperate with eIF4A in driving 5′UTR-dependent translation of 53BP1 will be important to further clarify this mechanism.”

We modified the manuscript as following:

Line 219: “*The 53BP1 5’UTR contains two predicted G quadruplex (G4) secondary structures (Fig.S4A). We next endogenously deleted a genomic region within the first exon of 53BP1 corresponding to nucleotides 41-95 of the 53BP1 5' UTR in A375 cells (Δ5’UTR) (Fig. 3D). This region encompasses one of the predicted G4, thus potentially altering secondary structure within 53BP1 5'UTR*”.

Line 299: “*The 53BP1 5’UTR contains two predicted G quadruplex (G4) secondary structures potentially sensitive to eIF4A inhibition, as well as several polypurine-rich sequences at a density comparable to those found in the 5’UTR of Cyclin D1, a well-established eIF4A-dependent mRNA (Fig. S4A, S5A).”*

A

Position	Length	QGRS	G-Score
16	29	GGCGGCGGTGGCGACAGCGGCGACCTAGG	21
54	14	GGAGGGACTTGGGG	17

B

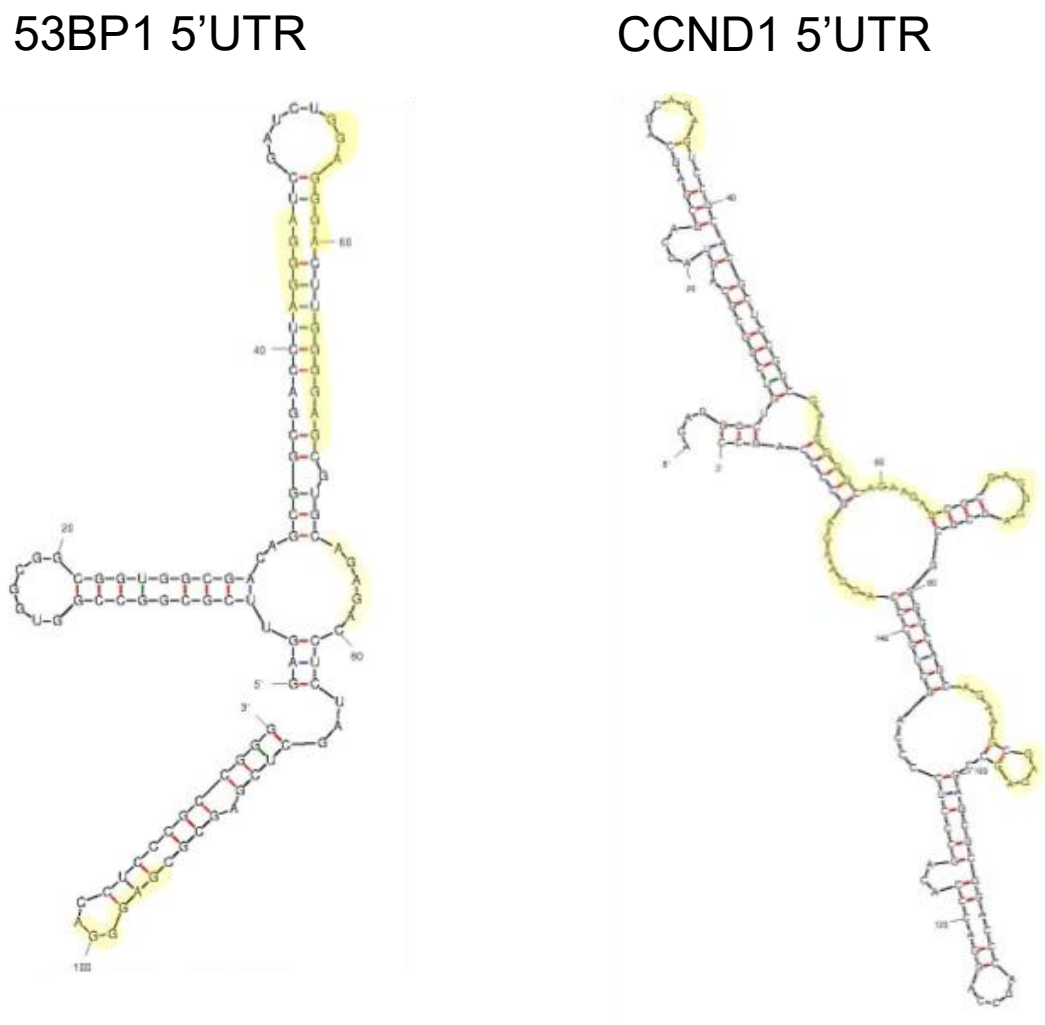

Figure R4: **A**. G quadruplex in the 5’untranslated region of 53BP1 mRNA using QGRS Mapper. **B**. Representation of the *53BP1* and *CCDN1* 5’ UTR using mfold software. Polypurine-rich sequences are highlighted in yellow.

7) Fig S4A should be moved to main Figures and Fig 3E can go to Supplementary Figures.

Thanks for the suggestion. Fig. S4A is now Fig. 3E and Fig. 3E is now the new Fig. S4B with the improvements requested by the other reviewers.

8) Fig 7D: Translation of the empty construct seems to increase in a dose-dependent manner. Why is this?

We observed a slight, non-statistically significant 1.5-fold increase in the Renilla (RLuc)/ Firefly (FLuc) ratio when cells transfected with the empty vector were treated with the eIF4A inhibitor RBX0901.

It has been reported that eIF4A inhibitors can reprogram the translational landscape beyond simple repression (PMID: 34644561, PMID: 40118049), although the mechanisms are not fully understood and may occur independently of eIF4A activity (PMID: 34644561, PMID: 40118049), particularly for eIF4A-insensitive mRNAs (PMID: 25273840). In this context, the empty reporter, which contains a minimal 5′UTR upstream of the Renilla coding sequence, is

expected to be less dependent on eIF4A helicase activity. Nonetheless, this tendency observed here is not statistically significant and does not alter the overall interpretation of the results.

9) Legends of Figures S3 and S4 seem to be swapped.

We thank the reviewer and apologize for the error. The legend of figures S3 and S4 have been corrected.

Referee #2 (Remarks for Author):

1. The study defines persisters as the 30% of cells surviving a 3-day treatment with BRAF/MEK inhibitors. However, this definition appears arbitrary and lacks biological consistency. Survival rates are highly dependent on drug dose and duration: longer or more intense treatment could reduce survival to 5%, while shorter exposure might leave 80% alive. Without comparative molecular profiling across these different survival thresholds, it is unclear whether the "30% persisters" represent a distinct, stable cell state or simply reflect stochastic survival due to variable drug sensitivity.

We fully agree that a definition based solely on cell surviving fraction is arbitrary and may lack biological consistency. Importantly, our study does not describe drug-tolerant cells based only on surviving fraction. Instead, our operational 3-day model reflects our previously published experimental framework (PMID: 31844050), which demonstrated that melanoma cells surviving 3-days of targeted therapy exhibit: (i) a non-genetically driven phenotype; (ii) the ability to tolerate otherwise lethal effects of the drugs; and (iii) a reversible and transient phenotype. We further confirmed the reversible BRAFi/MEKi tolerance again in A375 cells after 3 days of drug exposure (500 nM BRAFi/500 nM MEKi) (Fig. R5). These features are closely aligned with the current understanding of drug-tolerant cells. Similar frameworks of drug-exposure have been widely used to characterize a reversible, drug-induced molecular state (PMID: 31699882, PMID: 35232962, PMID: 34287642, PMID: 33724679 etc...) in “persister” cells.
For these reasons, we believe that our terminology and experimental approach are consistent with the literature and reflect a previously described drug-tolerant state, rather than an arbitrary definition based on survival percentage.

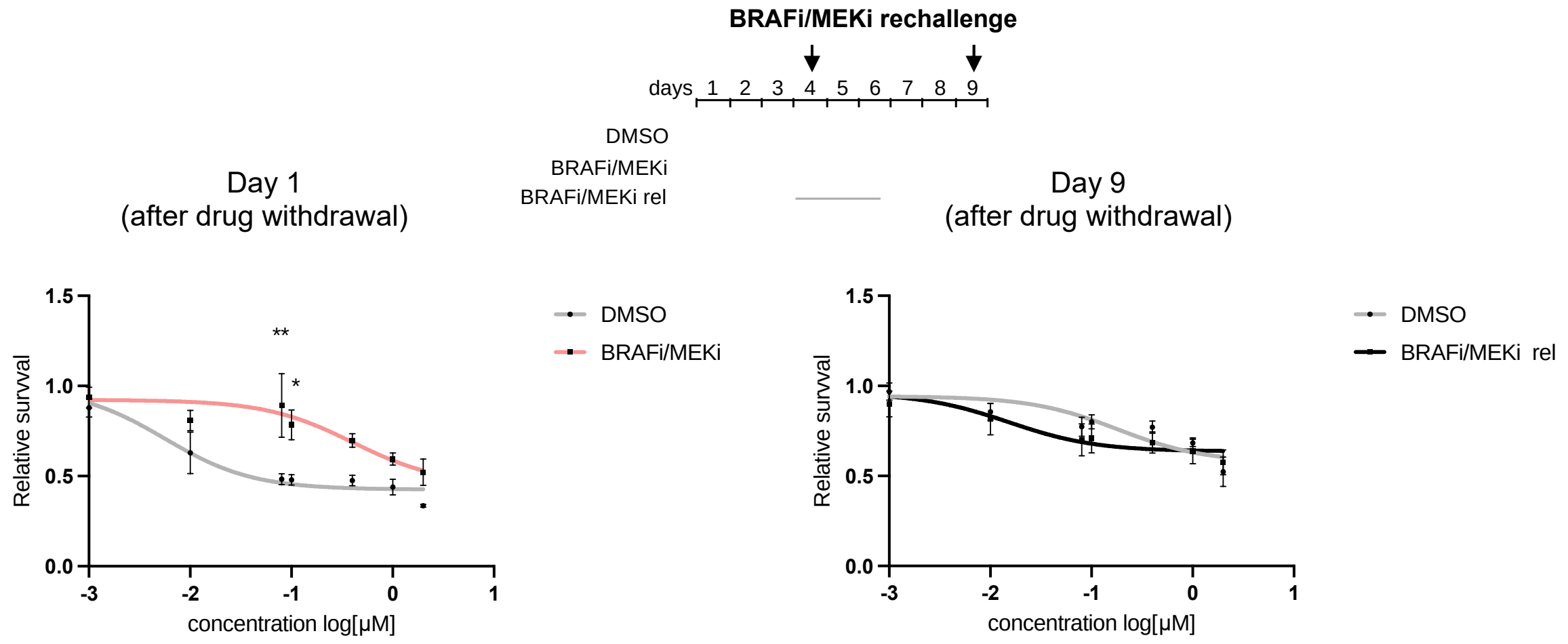

Figure R5: Dose-response curves showing reversivbe tolerance of cells that survived 3 days of BRAFi/MEKi treatment (BRAFi/MEKi) compared to control (DMSO) cells. Cells were re-challenged with increasing concentrations (log [µM]) of a BRAFi/MEKi cocktail. After 9 days of culture in drug-free medium (BRAFi/MEKi-rel), previously tolerant cells regained sensitivity comparable to control (DMSO) cells. Cell viability was assessed using the WST-1 assay.

Moreover, the functional relevance of this population to clinical persistence is uncertain. The link to human IHC data (Fig 1C) is tenuous-no scoring methodology is provided, and there is no evidence that the tumor samples contain true persister cells rather than heterogeneous or transiently stressed cells. The lack of a standardized, biologically grounded definition weakens the model's translational significance.

The IHC data, although limited due to the impossibility to extend our cohort of patients, were intended to assess 53BP1 expression in clinical specimens from patients treated with targeted therapies. Studying true residual persister cells, such as those involved in minimal residual disease in patients, is inherently challenging because they are undetectable using conventional image-based practices. Consequently, most studies investigating persister cells in human samples rely on specimens collected before treatment and during targeted therapy, typically when tumours are regressing or stable and can still be surgically resected (e.g., PMID: 32822576, PMID: 30017245, PMID: 37679568).
Moreover, biologically defining highly heterogeneous “persister” cells, in terms of biomarkers or plasticity, is an active field of investigation, and no cancer-wide consensus has yet been reached.
Finally, the scoring methodology has been added in the method section line 775:

“*Quantification of 53BP1 staining was performed by a pathologist using an H-score–based approach. Cells were classified according to staining intensity (1 = weak, 2 = moderate, 3 = strong, 4 = very strong), and the number of cells at each intensity level was recorded. A composite score was calculated as the sum of the number of cells at each intensity multiplied by the corresponding intensity, normalized to the analysed tissue area (µm²).*”

Notably, 53BP1 upregulation is not uniform across all persisters (Fig 1K), suggesting it is not a core, defining feature of the persister state but rather a variable response in a subset of cells. This heterogeneity further undermines claims of a coherent, programmed translational response.

The phenotypic and molecular heterogeneity of drug-tolerant cells, coexisting in the same cell population, is widely recognized and represents a major clinical challenge. Nevertheless, targetable molecular vulnerabilities have been identified within these heterogeneous populations, including in melanoma. In this context, although 53BP1 upregulation is not uniformly or consistently observed across all cells, our data support an overall increase in *53BP1* translation at the population level. RiboMap assay was further employed to assess translation regulation at the single-cell level.

2. A major alternative explanation is not sufficiently addressed: that 53BP1 upregulation is primarily transcriptional, driven by DNA damage rather than selective translation. BRAF/MEK inhibitors are known to induce replication stress and double-strand breaks (DSBs), activating the DNA damage response (DDR), which includes transcriptional upregulation of DDR genes such as 53BP1.
The authors claim no change in 53BP1 mRNA levels, but the qPCR data in Fig S1C show a 1.5- to 2-fold increase in 4 out of 7 conditions-changes that, while modest, may be biologically significant, especially given the limitations of qPCR in detecting subtle but consistent transcriptional shifts (PMID: 12184808). The absence of more rigorous quantification leaves open the possibility that transcriptional induction is occurring. In this alternative model, global translation is suppressed (due to eIF4F inhibition via ERK/mTOR blockade), but certain stress-induced mRNAs-like 53BP1-are still translated. Mutating 53BP1 or inhibiting eIF4A would then impair survival not because of disrupted translational reprogramming, but because cells fail to mount an essential DDR. This would explain the observed dependency on 53BP1 for long-term resistance and relapse in vivo-without invoking a novel, selective translational program.

The distinction between translational reprogramming and stress-induced transcription is crucial. The authors, experts in translational control, may be predisposed to interpret data through this lens, but they must more rigorously exclude the more parsimonious transcriptional model.

We carefully considered the transcriptional model of stress-dependent transcriptional regulation. Although the observed fold changes were not statistically significant, we agree that variability is present, which may arise from both technical and biological variances. To address this concern, we implemented four complementary strategies.
First, for the assessment of the biological variance, we increased the number of independent experimental replicates (n=6).
Second, to account for technical variance, we measured mRNA levels using multiple housekeeping genes (n=3, Fig. R6A).
Third, we applied the normalization strategy suggested by the reviewer and described in PMID: 12184808, which relies on the geometric mean of multiple housekeeping genes (Fig. R6B). Fourth, using the same set of replicates and the normalization approach based on geometric mean of multiple housekeeping genes, we compared the fold-change in *53BP1* mRNA following targeted therapy to that of a gene known to be transcriptionally regulated following
BRAFi/MEKi. Specifically, we selected NGFR (also referred to CD271 or p75) as it was previously reported to undergo transcriptional induction in response to targeted therapy in melanoma (PMID: 28069687, PMID: 30017245) (Fig. R7A-B).

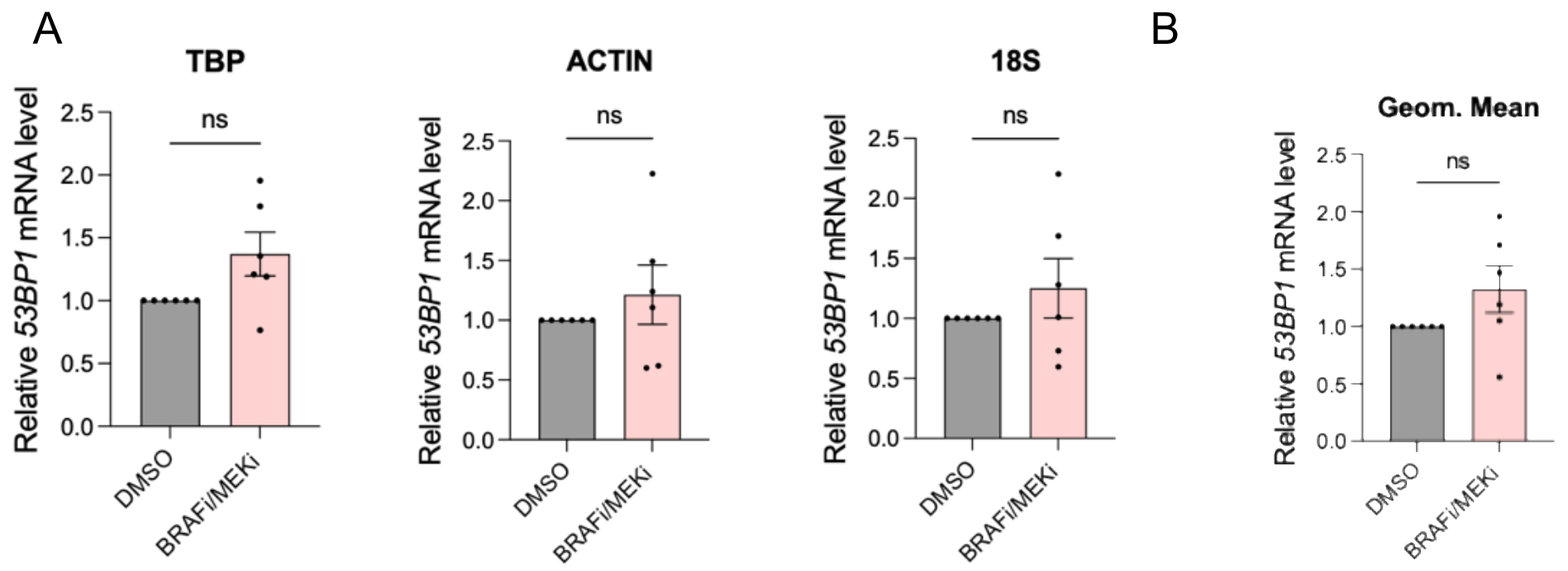

Figure R6: A, Quantification of 53BP1 mRNA level using RT-qPCR in A375 control cells (DMSO) and in cells surviving targeted therapy (BRAFi/MEKi). Data represent the mean ± SEM from 6 independent experiments. Data were normalized using the reported housekeeping genes (TBP, ACTIN, 18S). **B**, Quantification of 53BP1 mRNA level using RT-qPCR in A375 control cells (DMSO) and in cells surviving targeted therapy (BRAFi/MEKi). Data represent the mean ± SEM from 6 independent experiments. Data were normalized using the geometric mean of the housekeeping genes as in A.

As shown in Fig. R6A, increasing the number of biological replicates and using multiple housekeeping genes resulted in an average fold-change of 53BP1 mRNA levels of less than 1.5 in cells treated for 3 days with BRAFi/MEKi compared with control (DMSO) conditions. Despite the observed variability, these differences did not reach statistical significance. Applying the geometric averaging of the housekeeping genes yielded comparable results (Fig. R6B).
Finally, we analysed *NGFR* transcript levels in control A375 cells and in A375 cells treated for 3 days with BRAFi/MEKi (Fig. R7). Targeted therapy induced an approximately 1,000-fold increase in *NGFR* mRNA compared with DMSO-treated cells (Fig. R7A). When comparing the log2 fold-change values of 53BP1 and NGFR transcripts in BRAFi/MEKi-treated cells, we observed a log2 difference of 9 of NGFR compared to *53BP1* (Fig. R7B), corresponding to an approximate 500-fold difference in transcript levels.

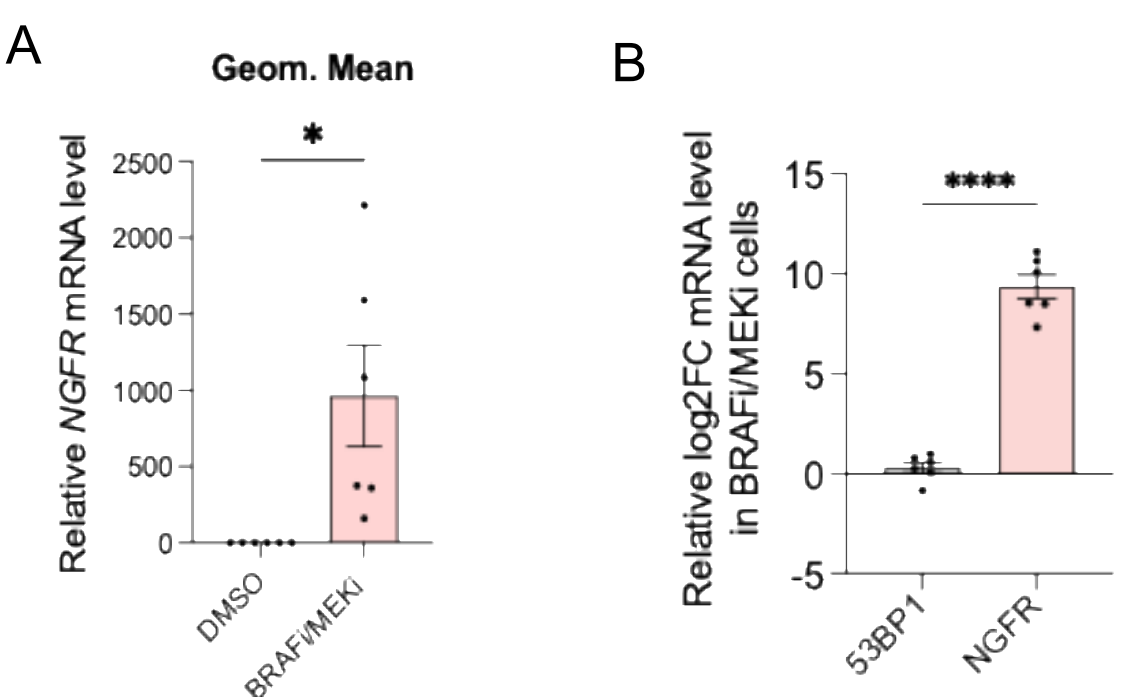

Figure R7: A, Quantification of *53BP1* mRNA level using RT-qPCR in A375 control cells (DMSO) and in cells surviving targeted therapy (BRAFi/MEKi). Data represent the mean ± SEM from 6 independent experiments. Data were normalized using the geometric mean of TBP, Actin and 18S housekeeping genes. **B**: RT-qPCR analysis of the indicated genes in A375 cells treated with BRAFi/MEKi for 3 days. Data are expressed in log2FC relative to control (DMSO) cells based on n=6 sample replicates analysed and normalized using the geometric mean of TBP, Actin and 18S housekeeping genes.

Taken together, these data demonstrate that *53BP1* mRNA levels do not increase in cells treated with BRAFi/MEKi for 3 days, thereby excluding a possible transcriptional induction.
The same approach was used to quantify *53BP1* mRNA level in M249 cells (Fig. R8), and the newly generated data have been added in the manuscript as Fig. S1C.

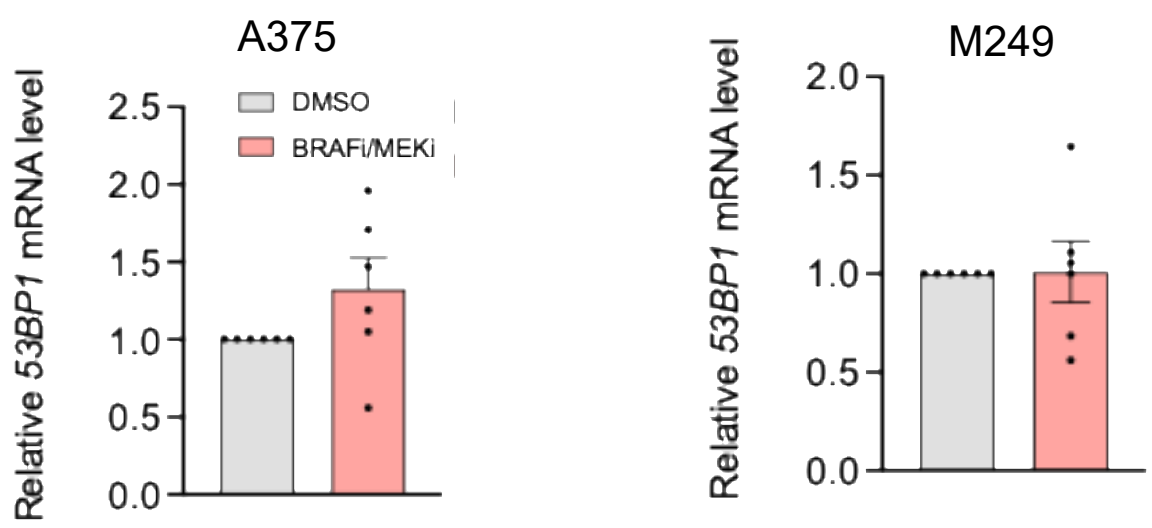

Figure R8: Quantification of *53BP1* mRNA level using RT-qPCR in control cells (DMSO) and in cells surviving targeted therapy (BRAFi/MEKi) in both A375 and M249 cells. Data represent the mean ± SEM from 6 independent experiments. Data were normalized using the geometric mean of TBP, Actin and 18S housekeeping genes.

In the Method section, we added the following text (line 617): "*For normalization, we applied the geometric mean of multiple housekeeping genes (TBP, 18S, Actin).*"

3. The evidence for translational control of 53BP1 relies heavily on indirect and technically limited assays. AHA labeling and Ribomap are used to infer translation, but Ribomap, in particular, is not a well-validated or widely adopted method. The punctate signals interpreted as translation sites require stronger validation-for example, disappearance upon cycloheximide treatment to confirm active translation. In addition, more standard approaches, such as polysome profiling with qPCR of 53BP1 mRNA across sucrose gradient fractions should be used.

We thank the reviewer for the suggestion to include an additional control for RiboMap assay. We have now assessed the ribosome association by using the translation inhibitor Harringtonine as previously described (PMID: 37384709), which cause ribosomes to accumulate at sites of translation initiation. We did not select CHX as it inhibits the elongation phase of translation, causing ribosomes to freeze along the entire coding sequence, making it unsuitable for RiboMap control experiments. Upon Harringtonine treatment, we observed a decrease in the RiboMap signal for both *53BP1* and *IQGAP1* mRNAs, in control A375 cells (DMSO) as well as in cells treated with targeted therapy treatment (BRAFi/MEKi) (Fig. R9). This experiment has been added as Fig. S2F of the manuscript.

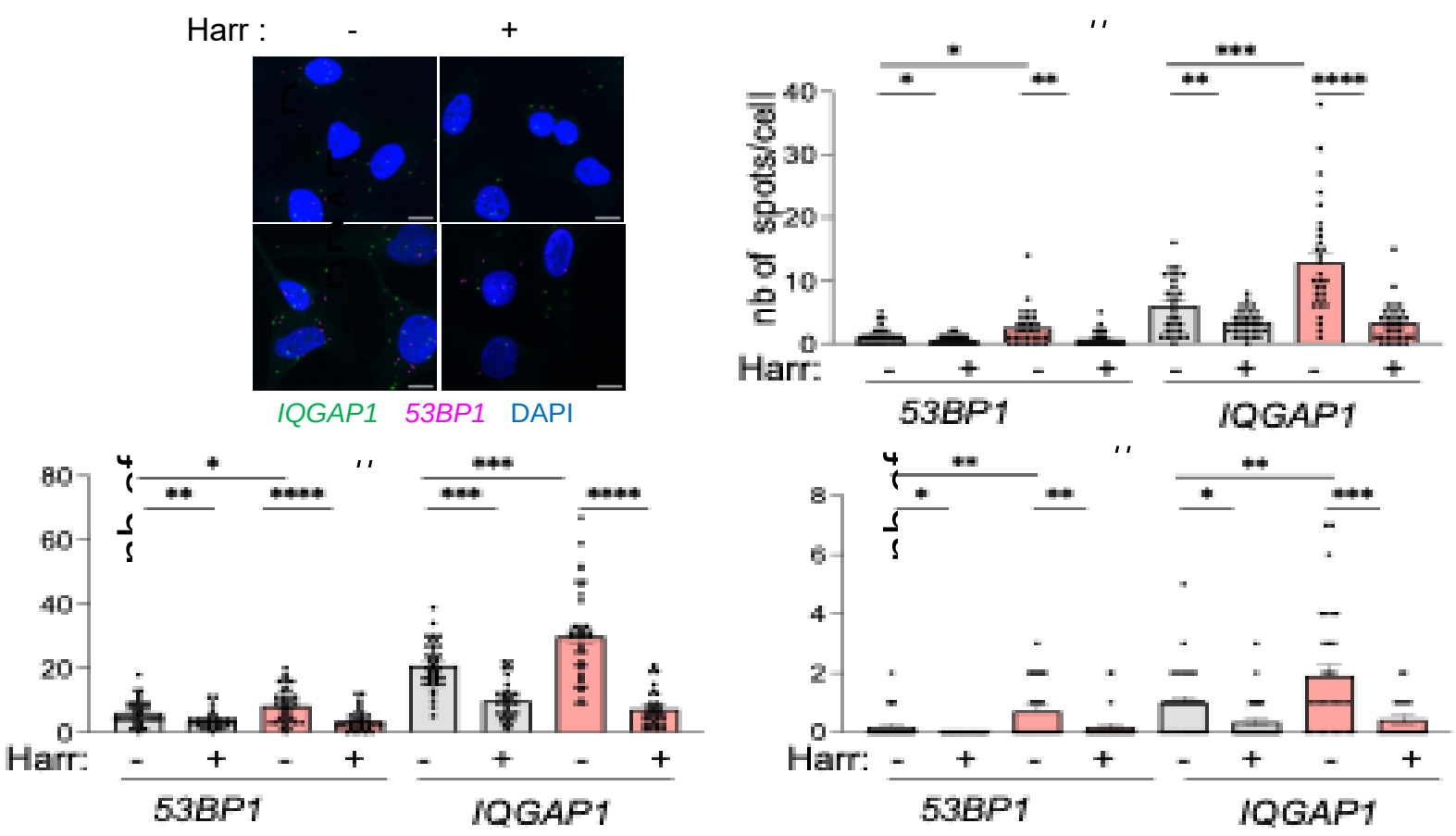

Figure R9: (top): Representative confocal images of translating 53BP1 (magenta) and IQGAP1 (green) mRNAs in DMSO and BRAFi/MEKi-treated cells in the presence or absence of Harringtonine (Harr) treatment (5 µM). Nuclei are stained with DAPI (blue). Scale bar: 10 µm. (bottom): Quantification of translating 53BP1 and IQGAP1 mRNAs (spots). Data represent the mean ± SEM of the number of spots/cells quantified in 3 independent experiments (* $p \leq 0.05$, ** $p \leq 0.01$,*** $p \leq 0.001$, **** $p \leq 0.0001$).

In the manuscript we added the following text (line 185):

"*The specificity of active translation detected by the RiboMap assay was further validated for both 53BP1 and IQGAP1 mRNAs using the translation initiation inhibitor harringtonine, which arrests ribosomes at the translation initiation sites. Harringtonine treatment led to a reduction in the number of actively translating 53BP1 and IQGAP1 mRNAs in both control (DMSO-treated) cells and cells that survived BRAFi/MEKi treatment (Fig.S2F), confirming that the RiboMap signal reflects actively translating mRNAs*."

In response to the suggestion to use more standard approaches, we note that we previously performed an extensive characterization of *53BP1* mRNA translation using polysome profiling coupled with qPCR (PMID: 31844050). Building on this established work, we therefore employed alternative, validated methods to assess the translational status of *53BP1* mRNA at both bulk and single-cell resolution.

4. In Figure 3, the authors use a dual-luciferase reporter to assess the translational activity of the 53BP1 5'UTR. However, the data are normalized to an empty vector control, despite the R/F (Renilla/Firefly) ratio inherently correcting for transfection efficiency and transcriptional activity. This additional normalization may artificially amplify differences and obscure true effects. The rationale for this approach is not explained, raising concerns about data interpretation.

We agree with the reviewer. We have now modified Fig 3B and C, as in Fig. R10, R11, presented without normalization to the empty vector. The interpretation of the results remains unchanged.

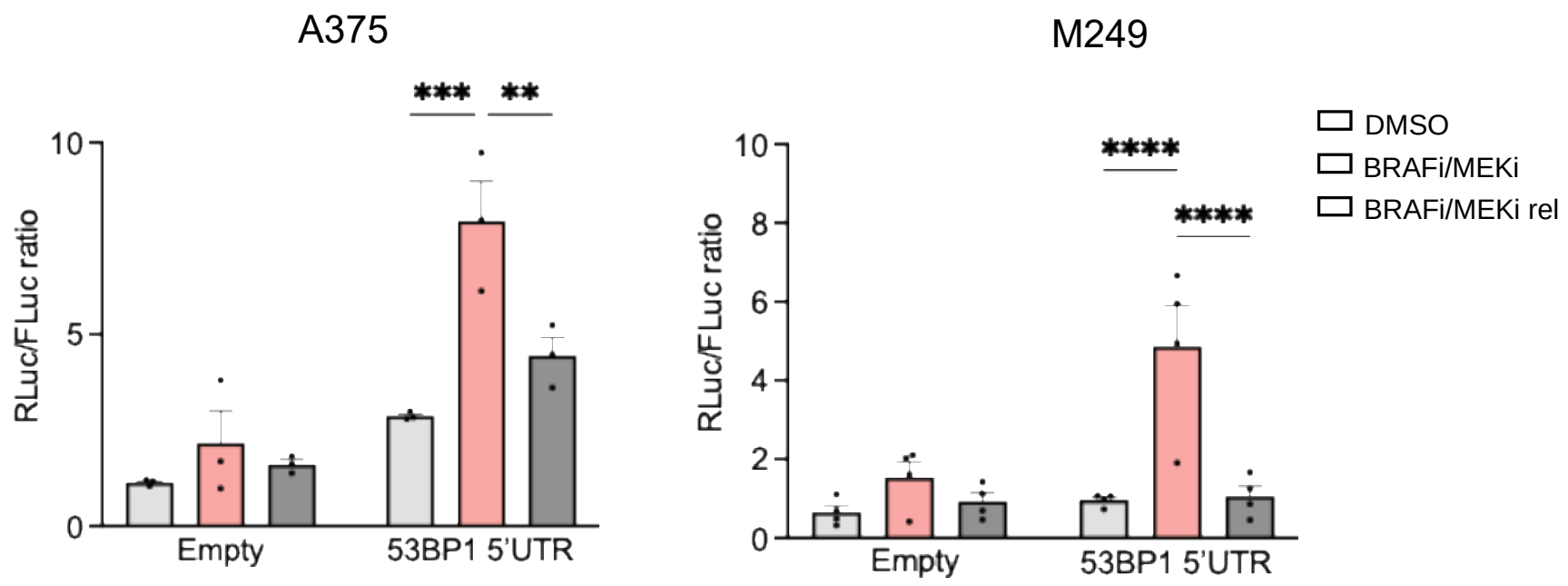

Figure R10: Quantification of the luciferase assay performed in A375 and M249 cells treated with DMSO or BRAFi/MEKi for 3 days, and in cells released from BRAFi/MEKi treatment (BRAFi/MEKi rel). Renilla (LucR) activity was measured 48h after transfection and the activity of the Firefly luciferase (LucF) was used as control of transfection. The data represent the ratio of Renilla (RLuc) to Firefly (FLuc) luciferase activities.

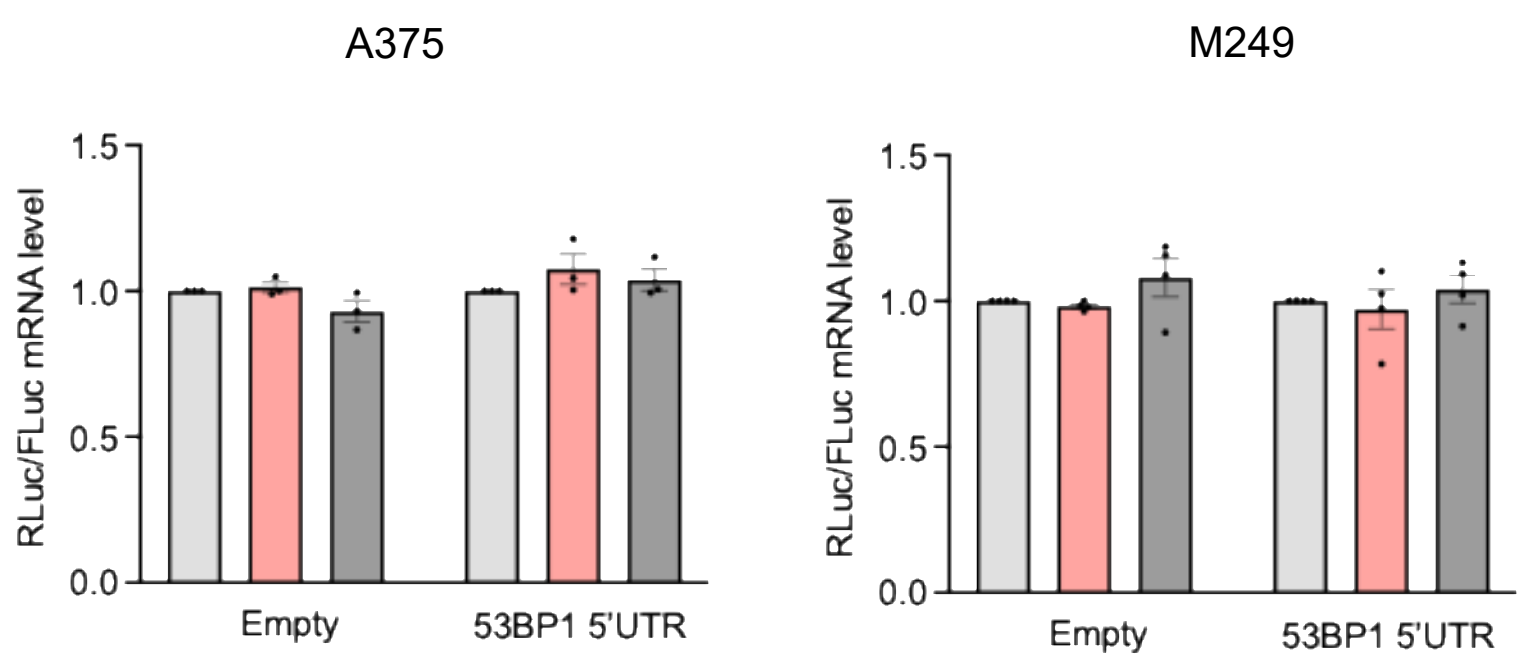

Figure R11: RT-qPCR quantification of the LucR/LucF mRNA level from the experiment in R10.

5. The CRISPR-mediated deletion of nucleotides 41-95 in the 53BP1 5'UTR lacks a clear rationale. No prior functional mapping (e.g., deletion series or mutagenesis) is presented to justify targeting this specific region. A more systematic approach-such as scanning the 5'UTR for regulatory elements via reporter assays-should be performed.

The *53BP1* 5′UTR is 113 nts-long. To investigate its role in regulating 53BP1 mRNA translation in an endogenous context, we performed a substantial deletion (nt 41–95), to the extent technically feasible, as a proof of concept. As noted above, this deleted region includes a predicted G-quadruplex (Fig. R4), which may account for the observed impairment of eIF4A-dependent translation. New Fig.S4A and S5A have been added.

Furthermore, the authors claim that the deletion does not affect 53BP1 mRNA levels, yet Fig 3E shows clear mRNA reduction in two of three clones, and Fig 3G also indicates mRNA changes. This discrepancy is problematic. Given that the 5'UTR overlaps with a strong H3K27ac mark-a chromatin signature of enhancers or promoters-this region may influence transcription. Disrupting it could therefore affect mRNA levels independently of translation, confounding the interpretation of phenotypic effects.

We applied the strategy as described in PMID: 12184808, which uses the geometric mean of multiple housekeeping genes, by assessing 6 independent biological replicates (Fig. R12). From these experiments we concluded, as above, that mRNA levels, despite showing variability, do not significantly change. This Figure is now Fig. S4B in the manuscript.

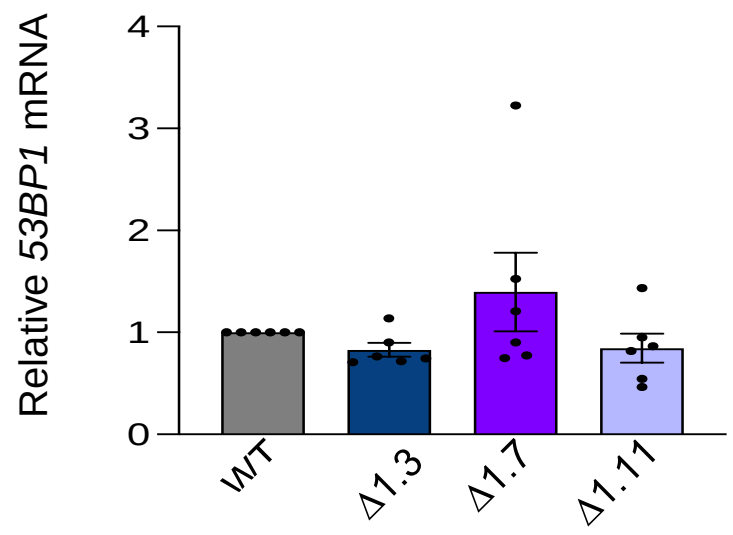

Figure R12: Quantification of *53BP1* mRNA level using RT-qPCR in A375 WT cells or in A375 Δ 5’UTR clones. Data represent the mean ± SEM from 6 independent experiments. Data represent the mean ± SEM from 6 independent experiments. Data were normalized using the geometric mean of TBP, Actin and 18S housekeeping genes.

Referee #3 (Remarks for Author):

1.Although figs 1-4 show results in both A375 and M249 cell lines, some results in other figures, in particular regarding NHEJ efficiency and eIF4A inhibitors, were carried out only in A375 cells. Further validation in additional cell lines would strengthen these conclusions.

We agree with the reviewer’s consideration, and we performed additional experiments in M249 cell line.
In particular, these include the assessment of NHEJ efficiency, added in Fig. S4E of the manuscript, and the effect of eIF4A inhibitors on 53BP1 protein levels and 53BP1 5’UTR-driven translation as assessed by luciferase assay (Fig. S5B-D).

2.Most of the conclusions are supported by solid and well replicated data. However, there

are some instances with large experimental variability in some key experiments, and authors reach to certain conclusions that may need revision/clarification. For example, authors conclude that 53BP1 mRNA does not increase upon BRAFMEKi treatment (Fig. S1C), but it looks like there is a trend towards an increase of mRNA. Off the 4 replicates, there is 1 with fold-change larger than 2, another with 1.7, another 1.4 and another 0.5. Did 53BP1 protein change to a similar extent in those 4 experiments? Fig.1E that shows 53BP1 protein levels by western blot has 3 replicates.

In response to this concern, which was also raised by another reviewer, we sought to minimize technical and biological variances in our qPCR experiments by increasing the number of biological replicates and applying the normalization strategy described in PMID: 12184808, based on the geometric mean of multiple housekeeping genes (Fig. R8). Using this approach, we confirmed that *53BP1* mRNA is not transcriptionally induced, whereas protein levels consistently increase.

Similar comment for other graphs:
-Fig.3E. Analysis of 53BP1 transcript levels in 3 different clones indicated that the 5'UTR deletion did not affect promoter functions (Fig. 3E). However, and although maybe not statistically significant, there is a reduction in 53BP1 mRNA in some replicates of D1.3 and D1.11.

We applied the same strategy as above (Fig. R12). This is now Fig. S4B.

-Fig.3G. authors conclude that mRNA is not affected, but there is a lot of variability, and in D1.3 and D1.11 there are several replicates with a 2-fold change increase in mRNA level, in particular for D1.3.

We applied the same strategy as above and, despite the variability, probably reflecting non-identical, heterogeneous responses to the applied stress, we do not observe significant changes (Fig. R13). This is Fig. 3G in the manuscript.

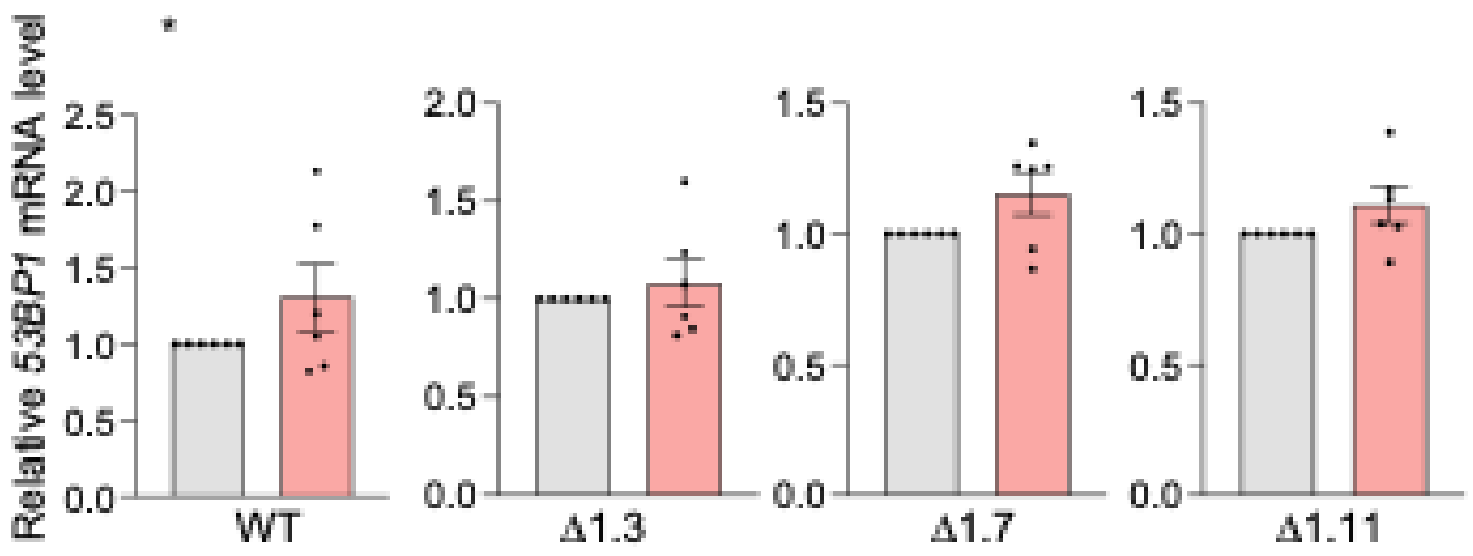

Figure R13: RT-qPCR quantification of 53BP1 mRNA level in A375 WT cells or in A375 Δ5'UTR clones. For each cell line, control cells (DMSO) or drug tolerant cells that survived 3 days of targeted therapy (BRAFi/MEKi) were analyzed for 53BP1 mRNA level. Data represent the mean ± SEM from 6 independent experiments. Data were normalized using the geometric mean of TBP, Actin and 18S housekeeping genes.

-Fig. S4C. 53BP1+ nuclei in the D5'UTR1.11, there is a lot of variability, but still there are 3 replicates that show almost 2-fold reduction vs DMSO average. Also, here one dot of the DMSO is almost hidden by the red box of BRAFiMEKi label.

As stated above, this variability is likely due to heterogeneous cellular responses to the treatment. Despite this, three independent replicates consistently show no increase in 53BP1, supporting the conclusions. We have also corrected the DMSO data point that was partially obscured by the BRAFi/MEKi label.

-Fig.5e. there are still important decreases (up to 50%) of lucR/lucF in Empty, not to dissimilar to some replicates in the 53BP1 5'UTR.

We note that, even if not statistically significant, a 40% decrease in the RLuc/FLuc ratio is observed in the empty vector condition. However, these effects do not reach the extent (85%) and significance observed with the 53BP1 5′UTR construct. In order to confirm the observed differences between the empty and 53BP1 5′UTR constructs, we have repeated the experiments (Fig. R13, red dots) and confirmed specific effects of 53BP1 5’UTR. This will be the new Fig. 5E in the manuscript.

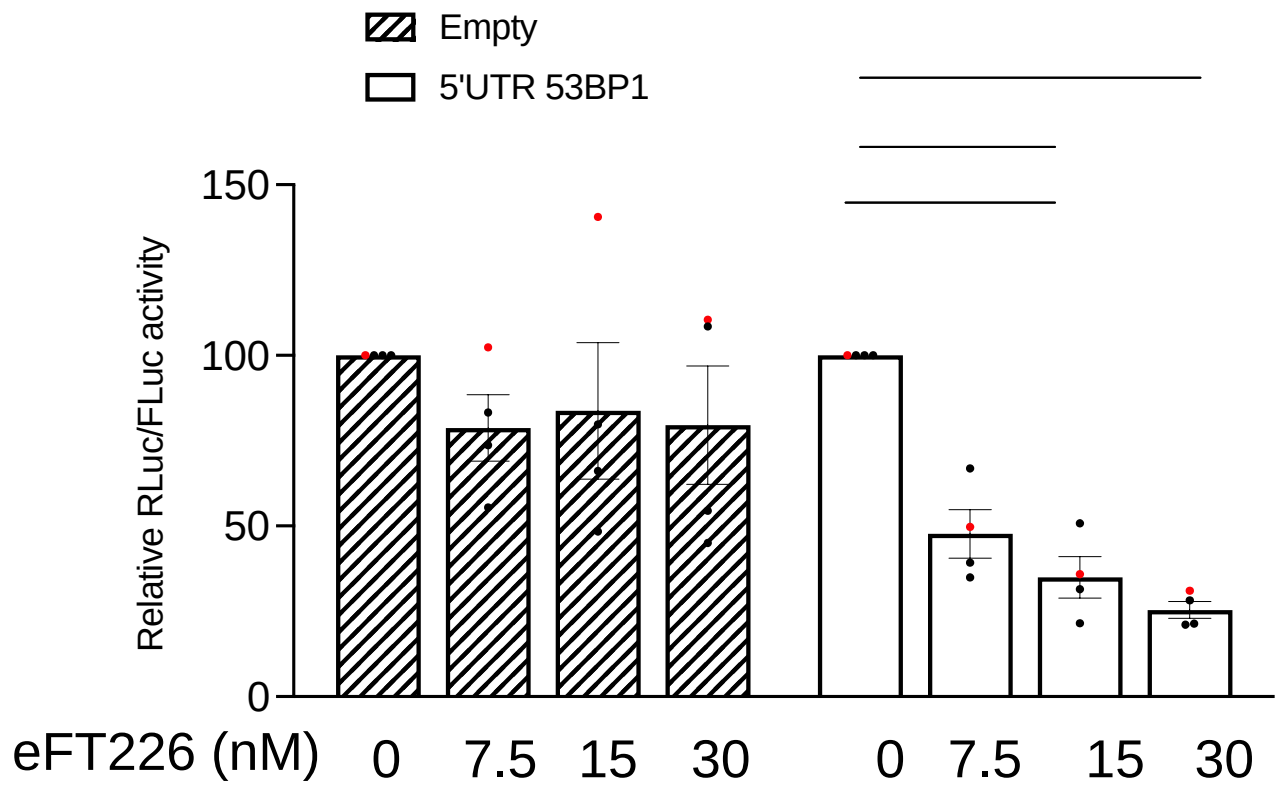

Figure R13: Quantification of the luciferase assay performed in A375 drug tolerant cells treated with the indicated concentrations of eFT226 for 12 hours. Renilla (LucR) activity was measured at the end of the eFT226 treatment, and the activity of the Firefly luciferase (LucF) was used as control of transfection. Data represent the effect of eFT226 on the luciferase activity of the reporter containing 53BP1 5’UTR or the Empty vector (without 53BP1 5’UTR) and are normalized to the eFT226 untreated conditions. The mean ± SEM from 4 independent experiments is reported (** p ≤ 0.01, *** p ≤ 0.001 **** p ≤ 0.0001).

Additional points that should be clarified/discussed:

4. Fig.3B. what are the first 3 bars vs the last 3??

We apologize for the omission. The Figure 3B has been changed according to the other reviewer suggestion (see R10) and labelling has been corrected.

5. Fig.3F some of these blots should be improved, in particular for D1.7 and D1.11.

Blots for Δ 1.7 and 1.11 have been changed in Fig 3F of the manuscript.

6. Fig. S8. "Additionally, RBX0901 tended to be more effective than eFT226 in reducing the translation of reporters containing the 5′UTRs of c-Myc and Cyclin D1,". This conclusion as is requires a statistical analysis side by side of the decrease of translation.

We have noted this and now added a new Figure S8A with the relative decrease in RLuc/FLuc activity of the c-Myc and Cyclin D1 5’UTR reporters (Fig. R15).

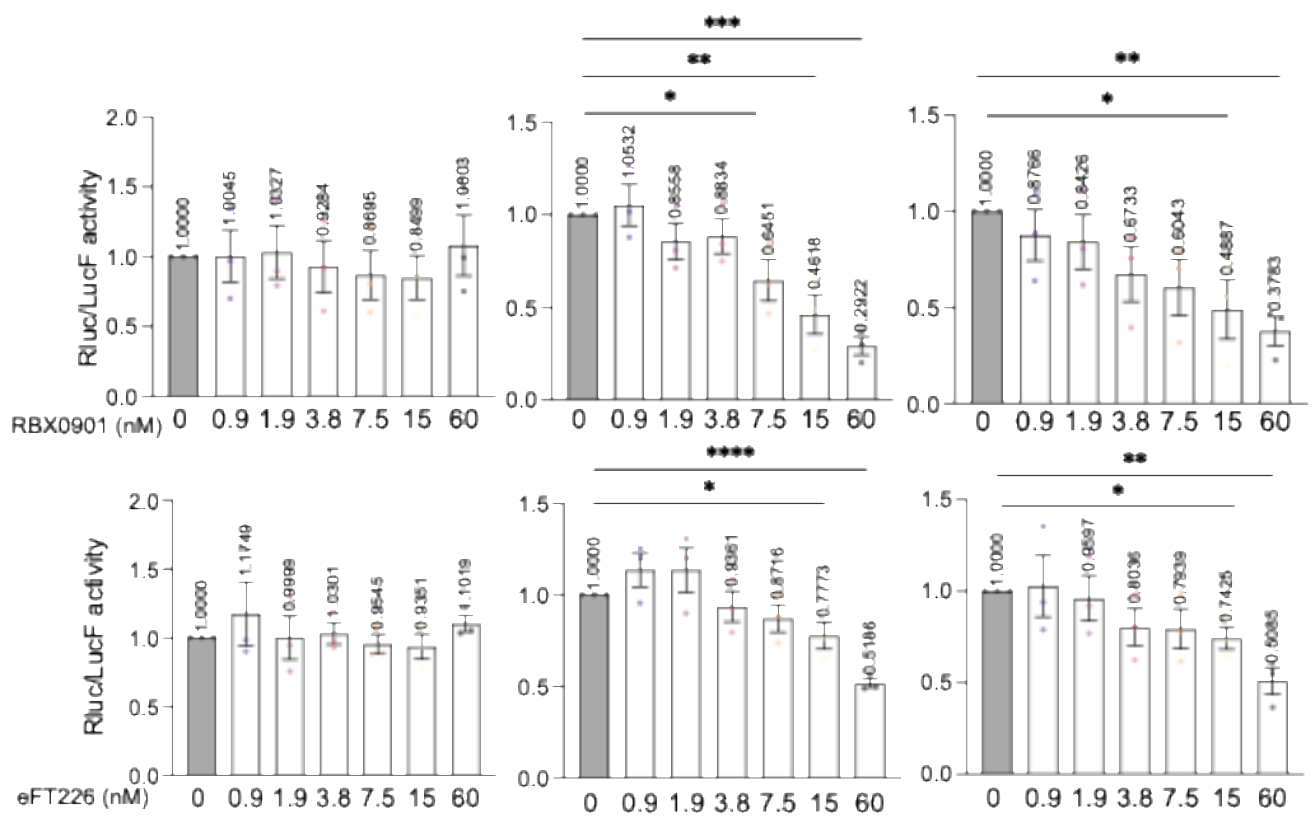

Figure R15: Quantification of the luciferase assay performed in A375 cells treated with the indicated concentrations of RBX0901 or eFT226 for 24 hours. Renilla (LucR) activity was measured 48h after transfection and the activity of the Firefly luciferase (LucF) was used as control of transfection. Data are normalized to the untreated conditions and represent the mean ± SEM from 3 independent experiments (* $p \leq 0.05$, ** $p \leq 0.01$, *** $p \leq 0.001$).

7. Fig 6A-B and 7E-F. Did authors test that the eIF4A inhibitor is acting on-target in vivo? For example, by assessing 53BP1 levels in the treated tumours. According to their in vitro data (Fig.5a), 53BP1 should increase with BRAFMEKi and decrease in the eFT226+BRAFMEKi-treated tumours.

Tumours treated with BRAF/MEK inhibitors, and even more prominently with the combination therapies, underwent rapid regression, which precluded the collection of tumour material for the analysis of 53BP1 levels.

8. Although methods say that for multiple comparisons ANOVA was used, there are a few graphs with several groups being analysed by t-test. For example, fig.1F, 4F..

We modified the method sections as following (line 834): "*Statistical analyses were performed using Prism 10 (GraphPad). Statistical significance, statistical tests, and sample sizes were determined as indicated in the figure legends. Significant is shown as p *<0.05, p **<0.01, p ***<0.001, p ****<0.0001 for all figures".* The statistical tests have now been added in the Figure legends.

11th May 2026

Dear Dr. Vagner,

Thank you for submitting your revised manuscript, and please accept my apologies for the delay in getting back to you, as one referee required additional time to provide their comments. Referees #1 and #3 have now evaluated the revised version of your study. As referee #2 was unavailable, referee #1 was also asked to evaluate your responses to their comments.
As you will see below, the referees are overall satisfied with the revisions, and I will therefore be able to accept your manuscript once the following points have been addressed:

1/ Referees' concerns:
Please address the remaining concerns from the referees.

2/ Manuscript text:
- Please remove the red font text and only keep in track changes mode any new modification.
- There is a discrepancy between Dorothée Baïlle (in the submission system) vs Dorothée Baille (in the manuscript), please adjust.
- Methods:
o Include a Reagents and Tools Table, which can be downloaded from our author guidelines and should be uploaded as a separate file.
o Add the methods from the supplementary information to the Methods in the manuscript text.
o Patient samples: Include a statement confirming that the experiments conformed to the principles set out in the WMA Declaration of Helsinki and the Department of Health and Human Services Belmont Report.
o Mice experiments: please indicate the housing and husbandry conditions.
o Statistics: Please include a statement about sample size, exclusion/inclusion criteria, blinding and randomization.
o If you used an image data base for scientific iconography (e.g., BioRender), indicate it at the end of the Methods section, and let us know if you have a license that allows for publication in an academic journal.
- Data Availability: Please move after the Methods, provide a URL for the deposited data, and note that the datasets must be public before acceptance of the manuscript.
- Author contributions: Remove from the manuscript file, and provide instead CRediT (Contributor Role Taxonomy) terms in the submission system. These replace a narrative author contribution section in the manuscript.
- Please rename "Competing interest" to "Disclosure and competing interests statement".
- References should be corrected to alphabetical order, with 10 author names listed before et al. and remove DOIs for published articles.

3/ Figures:
- Please upload the main and supplementary figures as separate figure files. Please rename the suppl. figures Figure EV1 - Figure EV9.
- Tables S1 - S3 should be renamed Table EV1 - EV3
- Figure S7 and S8 are called out before Figure S6, please correct to sequential order.
- Please address the queries from our data editors in the figure legends:
1. Please note that the exact p values are not provided in the legends of figures 1F, 2B, E, G, I; 3B, C, E, G; 4A, B, C, D, F; 5B, D, E, F, G; 6A, B; 7D, F, H, J.
2. Please indicate the statistical test used for data analysis in the legends of figures 1A, B
3. Please note that the exact p values are not provided in the legends of figures 1F, 2B, E, G, I; 3B, C, E, G; 4A, B, C, D, F; 5B, D, E, F, G; 6A, B; 7D, F, H, J.
4. Please indicate the statistical test used for data analysis in the legends of figures 1A, B

4/ Author checklist: please fill all subsections in "Experimental study design and statistics".

5/ At EMBO Press we ask authors to provide source data for the main manuscript figures. You will receive a separate email with instructions for providing source data with your revised manuscript, including how to upload and organize the files.

6/ Please provide The paper explained: EMBO Molecular Medicine articles are accompanied by a summary of the articles to emphasize the major findings in the paper and their medical implications for the non-specialist reader. Please provide a draft summary of your article highlighting
- the medical issue you are addressing,
- the results obtained and
- their clinical impact.
This may be edited to ensure that readers understand the significance and context of the research. Please refer to any of our published articles for an example.

7/ Please provide a 'Synopsis' to further enhance discoverability. Synopses include a short stand first (maximum of 300 characters, including space) as well as 2-5 one-sentences bullet points that summarizes the paper. Please write the bullet points to summarize the key NEW findings. They should be designed to be complementary to the abstract - i.e. not repeat the same text. We encourage inclusion of key acronyms and quantitative information (maximum of 30 words / bullet point). Please use the passive voice. Please attach these in a separate file or send them by email, we will incorporate them accordingly.

Please also suggest a visual abstract to illustrate your article as a PNG file 550 px wide x 300 px high.

8/ As part of the EMBO Publications transparent editorial process initiative (see our Editorial at http://embomolmed.embopress.org/content/2/9/329), EMBO Molecular Medicine will publish online a Review Process File (RPF) to accompany accepted manuscripts.
This file will be published in conjunction with your paper and will include the anonymous referee reports, your point-by-point response and all pertinent correspondence relating to the manuscript. Let us know whether you agree with the publication of the RPF and as here, if you want to remove or not any figures from it prior to publication.
Please note that the Authors checklist will be published at the end of the RPF.

I look forward to receiving your revised manuscript.

Yours sincerely,

Lise Roth

Lise Roth, PhD
Senior Editor
EMBO Molecular Medicine

***** Reviewer's comments *****

Referee #1 (Comments on Novelty/Model System for Author):

This is a high quality paper showing that 53BP1 mRNA translation enhancement promotes NHEJ and mutability of melanoma cells upon BRAFi/MEKi treatment. Mechanistically, this enhancement depends on regulatory elements in 53BP1 5'UTR, and is ameliorated by eIF4A inhibitors. The data identify a potential therapeutic target to prevent melanoma recurrence. The manuscript includes molecular, cellular and animal models, in addition to a modest number of paired human samples. The authors argue that they could not increase the latter despite their efforts.

Referee #1 (Remarks for Author):

The manuscript has improved with the new experiments. The authors show strong evidence for the involvement of 53BP1 mRNA translation in promoting NHEJ and mutability of melanoma cells upon drug treatment.
I think it would be useful for the reader that the authors include Fig R1B as supplementary material, and that that a scheme of 53BP 5'UTR with all its elements is shown in Figure 3, as originally suggested.

Referee #3 (Remarks for Author):

The authors have clarified/addressed most of the comments.
However, regarding the variability in 53BP1 mRNA levels, despite adding new replicates and the normalization approaches, there are still replicates that show important increases in 53BP1 mRNA. This limitation somehow should be stated in the manuscript.

EMM-2025-22756-V2-Q
Comments to the reviewers in black

Referee #1 (Comments on Novelty/Model System for Author):

This is a high quality paper showing that 53BP1 mRNA translation enhancement promotes NHEJ and mutability of melanoma cells upon BRAFi/MEKi treatment. Mechanistically, this enhancement depends on regulatory elements in 53BP1 5'UTR, and is ameliorated by eIF4A inhibitors. The data identify a potential therapeutic target to prevent melanoma recurrence. The manuscript includes molecular, cellular and animal models, in addition to a modest number of paired human samples. The authors argue that they could not increase the latter despite their efforts.

We thank the reviewer for the positive evaluation of our work despite the modest number of paired patient samples included.

Referee #1 (Remarks for Author):

The manuscript has improved with the new experiments. The authors show strong evidence for the involvement of 53BP1 mRNA translation in promoting NHEJ and mutability of melanoma cells upon drug treatment.
I think it would be useful for the reader that the authors include Fig R1B as supplementary material, and that that a scheme of 53BP 5'UTR with all its elements is shown in Figure 3, as originally suggested.

We thank the reviewer for these comments and suggestions. Fig. R1B is now the new Figure EV4I. In the manuscript, we added the following sentence (line 331): « Of note, stably resistant A375 cells (Shen *et al.*, 2019) are insensitive to 53BP1 depletion, as assessed by IncuCyte-based live-cell proliferation assay (Figure EV4I)."

We also modified Figure 3A by highlighting positions of G4 and polypurine motifs in 53BP1 5'UTR.

Referee #3 (Remarks for Author):

The authors have clarified/addressed most of the comments.
However, regarding the variability in 53BP1 mRNA levels, despite adding new replicates and the normalization approaches, there are still replicates that show important increases in 53BP1 mRNA. This limitation somehow should be stated in the manuscript.

We thank the reviewer for this comment. We have added the following statement in the manuscript (line 153) : « BRAFi/MEKi treatment led to a reversible increase in 53BP1 protein levels, as analysed by western blot (Figure 1E), without significant changes in *53BP1* mRNA levels despite an observed increase (less than 2-fold) in 2 out of 6 replicates in A375 cells (Figure EV1C). »

19th Jun 2026

Dear Dr. Vagner,

Thank you for submitting your revised files. I am pleased to inform you that your manuscript is accepted for publication and is now being sent to our publisher to be included in the next available issue of EMBO Molecular Medicine.

Your manuscript will be processed for publication by EMBO Press. It will be copy edited and you will receive page proofs prior to publication. Please note that you will be contacted by Springer Nature Author Services to complete licensing and payment information.

You may qualify for financial assistance for your publication charges - either via a Springer Nature fully open access agreement or an EMBO initiative. Check your eligibility: https://link.springer.com/journal/44321/how-to-publish-with-us

Should you be planning a Press Release on your article, please notify the production team via this form in order to coordinate publication and release dates: https://forms.cloud.microsoft/e/eH4A8JVP6g

If you have any questions, please do not hesitate to contact the Editorial Office. Thank you for your contribution to EMBO Molecular Medicine.

With kind regards,

Lise Roth

Lise Roth, Ph.D
Senior Editor
EMBO Molecular Medicine

>>> Please note that it is EMBO Molecular Medicine policy for the transcript of the editorial process (containing referee reports and your response letter) to be published as an online supplement to each paper. If you do NOT want this, you will need to inform the Editorial Office via email immediately. More information is available here: https://link.springer.com/partners/embo-press/editorial-policies#Peer%20review

EMBO Press Author Checklist

Corresponding Author Name: Lucilla Fabbri, Stéphan Vagner
Journal Submitted to: EMBO Molecular Medecine
Manuscript Number:EMM-2025-22756

USEFUL LINKS FOR COMPLETING THIS FORM
The EMBO Journal - Author Guidelines
EMBO Reports - Author Guidelines
Molecular Systems Biology - Author Guidelines
EMBO Molecular Medicine - Author Guidelines

Reporting Checklist for Life Science Articles (updated January
This checklist is adapted from Materials Design Analysis Reporting (MDAR) Checklist for Authors. MDAR establishes a minimum set of requirements in transparent reporting in the life sciences (see Statement of Task: 10.31222/osf.io/9sm4x). Please follow the journal's guidelines in preparing your
Please note that a copy of this checklist will be published alongside your article.

Abridged guidelines for figures
1. Data
The data shown in figures should satisfy the following conditions:
- the data were obtained and processed according to the field's best practice and are presented to reflect the results of the experiments in an accurate and unbiased manner.
- ideally, figure panels should include only measurements that are directly comparable to each other and obtained with the same assay.
- plots include clearly labeled error bars for independent experiments and sample sizes. Unless justified, error bars should not be shown for technical
- if n<5, the individual data points from each experiment should be plotted. Any statistical test employed should be justified.
- Source Data should be included to report the data underlying figures according to the guidelines set out in the authorship guidelines on Data

2. Captions
Each figure caption should contain the following information, for each panel where they are relevant:
- a specification of the experimental system investigated (eg cell line, species name).
- the assay(s) and method(s) used to carry out the reported observations and measurements.
- an explicit mention of the biological and chemical entity(ies) that are being measured.
- an explicit mention of the biological and chemical entity(ies) that are altered/varied/perturbed in a controlled manner.
- the exact sample size (n) for each experimental group/condition, given as a number, not a range;
- a description of the sample collection allowing the reader to understand whether the samples represent technical or biological replicates (including how many animals, litters, cultures, etc.).
- a statement of how many times the experiment shown was independently replicated in the laboratory.
- definitions of statistical methods and measures:
  - common tests, such as t-test (please specify whether paired vs. unpaired), simple χ2 tests, Wilcoxon and Mann-Whitney tests, can be unambiguously identified by name only, but more complex techniques should be described in the methods section;
  - are tests one-sided or two-sided?
  - are there adjustments for multiple comparisons?
  - exact statistical test results, e.g., P values = x but not P values < x;
  - definition of 'center values' as median or average;
  - definition of error bars as s.d. or s.e.m.

Please complete ALL of the questions below.
Select "Not Applicable" only when the requested information is not relevant for your study.

Materials

Newly Created Materials	Information included in the manuscript?	In which section is the information available? (Reagents and Tools Table, Materials and Methods, Figures, Data Availability Section)
New materials and reagents need to be available; do any restrictions apply?	Yes	Reagents and Tools Table

Antibodies	Information included in the manuscript?	In which section is the information available? (Reagents and Tools Table, Materials and Methods, Figures, Data Availability Section)
For **antibodies** provide the following information:<br>- Commercial antibodies: RRID (if possible) or supplier name, catalogue number and or/clone number<br>- Non-commercial: RRID or citation	Yes	Reagents and Tools Table

DNA and RNA sequences	Information included in the manuscript?	In which section is the information available? (Reagents and Tools Table, Materials and Methods, Figures, Data Availability Section)
**Short novel DNA or RNA including primers, probes**: provide the sequences.	Yes	Table EV2

Cell materials	Information included in the manuscript?	In which section is the information available? (Reagents and Tools Table, Materials and Methods, Figures, Data Availability Section)
**Cell lines:** Provide species information, strain. Provide accession number in repository **OR** supplier name, catalog number, clone number, and/**OR** RRID.	Yes	Reagents and Tolls Table
**Primary cultures:** Provide species, strain, sex of origin, genetic modification status.	Not Applicable	
Report if the cell lines were recently **authenticated** (e.g., by STR profiling) and tested for mycoplasma contamination.	Yes	Material and Methods

Experimental animals	Information included in the manuscript?	In which section is the information available? (Reagents and Tools Table, Materials and Methods, Figures, Data Availability Section)
**Laboratory animals or Model organisms:** Provide species, strain, sex, age, genetic modification status. Provide accession number in repository **OR** supplier name, catalog number, clone number, **OR** RRID.	Yes	Material and Methods and Reagents and Tools Table
**Animal observed in or captured from the field:** Provide species, sex, and age where possible.	Not Applicable	
Please detail **housing and husbandry conditions**.	Yes	Material and Methods

Plants and microbes	Information included in the manuscript?	In which section is the information available? (Reagents and Tools Table, Materials and Methods, Figures, Data Availability Section)
**Plants:** provide species and strain, ecotype and cultivar where relevant, unique accession number if available, and source (including location for collected wild specimens).	Not Applicable	
**Microbes:** provide species and strain, unique accession number if available, and source.	Not Applicable	

Human research participants	Information included in the manuscript?	In which section is the information available? (Reagents and Tools Table, Materials and Methods, Figures, Data Availability Section)
If collected and within the bounds of privacy constraints report on age, sex and gender or ethnicity for all study participants.	Yes	Material and Methods

Core facilities	Information included in the manuscript?	In which section is the information available? (Reagents and Tools Table, Materials and Methods, Figures, Data Availability Section)
If your work benefited from core facilities, was their service mentioned in the acknowledgments section?	Yes	Acknowledgements section

Design

Study protocol	Information included in the manuscript?	In which section is the information available? (Reagents and Tools Table, Materials and Methods, Figures, Data Availability Section)
If study protocol has been **pre-registered, provide DOI in the manuscript**. For clinical trials, provide the trial registration number **OR** cite DOI.	Not Applicable	
Report the **clinical trial registration number** (at ClinicalTrials.gov or equivalent), where applicable.	Not Applicable	

Laboratory protocol	Information included in the manuscript?	In which section is the information available? (Reagents and Tools Table, Materials and Methods, Figures, Data Availability Section)
Provide DOI OR other citation details if **external detailed step-by-step protocols** are available.	Not Applicable	

Experimental study design and statistics	Information included in the manuscript?	In which section is the information available? (Reagents and Tools Table, Materials and Methods, Figures, Data Availability Section)
Include a statement about **sample size** estimate even if no statistical methods were used.	Yes	Material and Methods
Were any steps taken to minimize the effects of subjective bias when allocating animals/samples to treatment (e.g. **randomization procedure**)? If yes, have they been described?	Yes	Material and Methods
Include a statement about **blinding** even if no blinding was done.	Yes	Material and Methods
Describe **inclusion/exclusion criteria** if samples or animals were excluded from the analysis. Were the criteria pre-established?<br>If sample or data points were omitted from analysis, report if this was due to attrition or intentional exclusion and provide justification.	Yes	Material and Methods
For every figure, are **statistical tests** justified as appropriate? Do the data meet the assumptions of the tests (e.g., normal distribution)? Describe any methods used to assess it. Is there an estimate of variation within each group of data? Is the variance similar between the groups that are being statistically compared?	Yes	Material and Methods and Figure Legends

Sample definition and in-laboratory replication	Information included in the manuscript?	In which section is the information available? (Reagents and Tools Table, Materials and Methods, Figures, Data Availability Section)
In the figure legends: state number of times the experiment was **replicated** in laboratory.	Yes	Material and Methods and Figure Legends
In the figure legends: define whether data describe **technical or biological replicates**.	Yes	Material and Methods and Figure Legends

Ethics

Ethics	Information included in the manuscript?	In which section is the information available? (Reagents and Tools Table, Materials and Methods, Figures, Data Availability Section)
Studies involving **human participants**: State details of **authority granting ethics approval** (IRB or equivalent committee(s), provide reference number for approval.	Yes	Material and Methods
Studies involving **human participants**: Include a statement confirming that **informed consent** was obtained from all subjects and that the experiments conformed to the principles set out in the WMA Declaration of Helsinki and the Department of Health and Human Services Belmont Report.	Yes	Material and Methods
Studies involving **human participants:** For publication of **patient photos**, include a statement confirming that consent to publish was obtained.	Not Applicable	
Studies involving experimental **animals**: State details of **authority granting ethics approval** (IRB or equivalent committee(s), provide reference number for approval. Include a statement of compliance with ethical regulations.	Not Applicable	
Studies involving **specimen and field samples:** State if relevant **permits** obtained, provide details of authority approving study; if none were required, explain why.	Not Applicable	

Dual Use Research of Concern (DURC)	Information included in the manuscript?	In which section is the information available? (Reagents and Tools Table, Materials and Methods, Figures, Data Availability Section)
Could your study fall under dual use research restrictions? Please check biosecurity documents and list of **select agents and toxins** (CDC): https://www.selectagents.gov/sat/list.htm	Not Applicable	
If you used a select agent, is the security level of the lab appropriate and reported in the manuscript?	Not Applicable	
If a study is subject to dual use research of concern regulations, is the name of the **authority granting approval and reference number** for the regulatory approval provided in the manuscript?	Not Applicable	

Reporting

The MDAR framework recommends adoption of discipline-specific guidelines, established and endorsed through community initiatives. Journals have their own policy about requiring specific guidelines and recommendations to complement MDAR.

Adherence to community standards	Information included in the manuscript?	In which section is the information available? (Reagents and Tools Table, Materials and Methods, Figures, Data Availability Section)
State if relevant guidelines or checklists (e.g., **ICMJE, MIBBI, ARRIVE, PRISMA**) have been followed or provided.	Not Applicable	
For **tumor marker prognostic studies,** we recommend that you follow the **REMARK** reporting guidelines (see link list at top right). See author guidelines, under 'Reporting Guidelines'. Please confirm you have followed these guidelines.	Not Applicable	
For **phase II and III randomized controlled trials**, please refer to the **CONSORT** flow diagram (see link list at top right) and submit the CONSORT checklist (see link list at top right) with your submission. See author guidelines, under 'Reporting Guidelines'. Please confirm you have submitted this list.	Not Applicable	

Data Availability

Data availability	Information included in the manuscript?	In which section is the information available? (Reagents and Tools Table, Materials and Methods, Figures, Data Availability Section)
Have **primary datasets** been deposited according to the journal's guidelines (see 'Data Deposition' section) and the respective accession numbers provided in the Data Availability Section?	Yes	Material and Methods
Were **human clinical and genomic datasets** deposited in a public access-controlled repository in accordance to ethical obligations to the patients and to the applicable consent agreement?	Not Applicable	
Are **computational models** that are central and integral to a study available without restrictions in a machine-readable form? Were the relevant accession numbers or links provided?	Not Applicable	
If publicly available data were reused, provide the respective **data citations in the reference list**.	Not Applicable	
